# Catalytic activity of graphene-covered non-noble metals governed by proton penetration in electrochemical hydrogen evolution reaction

**DOI:** 10.1038/s41467-020-20503-7

**Published:** 2021-01-08

**Authors:** Kailong Hu, Tatsuhiko Ohto, Yuki Nagata, Mitsuru Wakisaka, Yoshitaka Aoki, Jun-ichi Fujita, Yoshikazu Ito

**Affiliations:** 1 Institute of Applied Physics, Graduate School of Pure and Applied Sciences, University of Tsukuba, Tsukuba, 305-8573 Japan; 2 Graduate School of Engineering Science, Osaka University, 1-3 Machikaneyama, Toyonaka, 560-8531 Japan; 3 Max Planck Institute for Polymer Research, Ackermannweg 10, 55128 Mainz, Germany; 4 Graduate School of Engineering, Toyama Prefectural University, 5180 Kurokawa, Imizu, Toyama, 939-0398 Japan; 5 PRESTO, Japan Science and Technology Agency, Saitama, 332-0012 Japan; 6 Faculty of Engineering, Hokkaido University, N13W8 Kita-ku, Sapporo, 060-8628 Japan

**Keywords:** Heterogeneous catalysis, Electrocatalysis, Mechanical and structural properties and devices

## Abstract

Graphene-covering is a promising approach for achieving an acid-stable, non-noble-metal-catalysed hydrogen evolution reaction (HER). Optimization of the number of graphene-covering layers and the density of defects generated by chemical doping is crucial for achieving a balance between corrosion resistance and catalytic activity. Here, we investigate the influence of charge transfer and proton penetration through the graphene layers on the HER mechanisms of the non-noble metals Ni and Cu in an acidic electrolyte. We find that increasing the number of graphene-covering layers significantly alters the HER performances of Ni and Cu. The proton penetration explored through electrochemical experiments and simulations reveals that the HER activity of the graphene-covered catalysts is governed by the degree of proton penetration, as determined by the number of graphene-covering layers.

## Abstract (web summary)

Graphene-covering technology provides a promising approach for achieving a non-noble-metal-catalyst with corrosion protection and catalytic activity under acidic media. Here, the authors unveil that the electrochemical hydrogen evolution mechanism is governed by the proton penetration phenomenon.

## Introduction

Hydrogen (H_2_) generation is essential in an efficient and eco-friendly society because H_2_ is a clean and versatile fuel with an energy density of 140 MJ/kg^1^, which is much higher than that of fossil fuels such as gasoline, natural gas, and coal (~50 MJ/kg). Among various H_2_ generation technologies^2–4^, the electrolytic production of H_2_ from water is a promising method^3,5^ because it yields high-purity H_2_ (>95%) and emits no CO_2_. Polymer electrolyte membrane (PEM) water electrolysis is likely to be the next-generation technology for H_2_ production; it is more efficient than alkaline water electrolysis because of its H_2_ gas purity (>99.99 vol.% for PEM vs. 99.5 vol.% for alkaline), faster dynamic response, larger current density (~5 A cm^−2^ for PEM vs. ~0.45 A cm^−2^ for alkaline), and higher discharge H_2_ pressure (30–76 bar for PEM vs. 30–40 bar for alkaline)^3,6^.

One of the challenges associated with the PEM electrolysers is the degradation of the cathode catalyst under acidic conditions. Platinum (Pt) has frequently been employed as the cathode catalyst because it exhibits a high electrochemical activity and is chemically stable even in strongly acidic environments. However, the scarcity and high cost of noble-metal catalysts limit the use of PEM electrolysers. Given this limitation, it is important to develop cost-efficient, acid-stable, noble-metal-free catalysts that possess high hydrogen evolution reaction (HER) activity comparable to Pt and are chemically tolerant to acidic conditions.

The main source of HER catalyst degradation in acidic media is corrosion. This corrosion can be suppressed through passivation or covering of the catalyst surface with chemically stable graphene layers^7–10^. For example, graphene-encapsulated Ni, Fe, and CoNi alloy nanoparticles have been used in acidic electrolytes^11–13^. These encapsulated nanoparticles show sufficient tolerance against corrosion yet maintain their HER performance because the outermost graphene layers become HER-active owing to charge transfer from the underlying metal substrate^13^. However, regardless of the charge transfer effect, the performance of graphene-covered non-noble metal catalysts seems to be strongly dependent on the underlying metal substrate^11–13^. Here, a fundamental question is: how can the HER effectively occur with graphene layer covering, where the covering graphene blocks the catalytically active sites on the surfaces of the non-noble metal catalysts? Although the HER mechanism can be understood by the charge transfer^13^ and proton penetration through graphene^14,15^, this has not been evidenced by experiments. Therefore, it is crucial to determine how both the charge transfer and the proton penetration through graphene affect the HER mechanism of the graphene-covered non-noble metal catalysts.

In this study, we investigate the HER mechanisms of the proton penetration/charge transfer through the graphene-covering layers and their impact on the catalytic activity of graphene-covered non-noble metal catalysts. We find that protons as well as molecular hydrogen (H_2_) penetrate by bypassing the N-dopant-induced defect sites, which makes the catalysts covered by N-doped graphene electrochemically more active than those covered by non-doped graphene. Our results reveal that the charge transfer effect is not a dominant factor, while the ability of protons to penetrate graphene governs the HER activity of graphene-covered non-noble metal catalysts regardless of morphology and composition. This study provides new insights for the development of non-noble metal catalysts for electrochemical hydrogen production through graphene covering by balancing the contrasting properties of corrosion resistance and catalytic activity in acidic media.

## Results

### Impact of charge transfer and proton penetration on the catalytic activity of graphene-covered non-noble metal catalysts

**Fig. 1:**
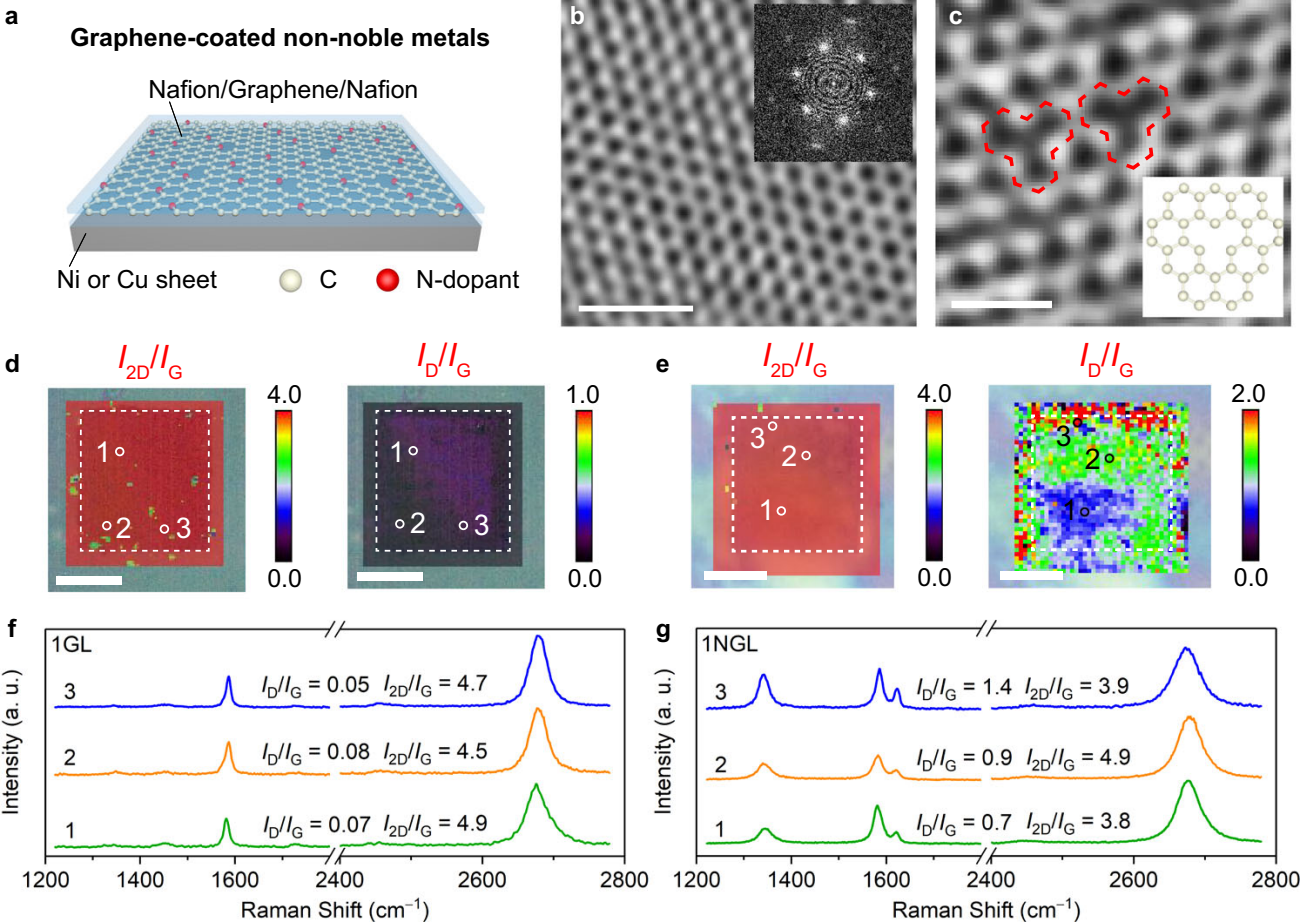
Graphene-covered non-noble metal catalysts and graphene characterization. **a** Schematic diagram of a graphene-covered Cu or Ni sheet. HRTEM images of (**b**) 1GL and (**c**) 1NGL. Inset of (**b**): the corresponding fast Fourier transform (FFT) image. Inset of (**c**): the atomic model of the structural defect, highlighted by red marks. Raman maps of (**d**) 1GL and (**e**) 1NGL performed on a window-attached Si_3_N_4_ chip. The dotted square indicates the window area. **f**, **g** Raman spectra were collected at the positions indicated on the corresponding maps. The *I*_2D_/*I*_G_ values provide evidence that monolayer graphene dominates. Scale bars: (**b**) 1 nm; (**c**) 0.5 nm; (**d**, **e**) 5 μm.

To evaluate the effects of charge transfer and proton penetration on the graphene-covered non-noble metal catalysts, we prepared a graphene-covered 50-μm-thick Cu sheet as a less-active HER system and a 50-μm-thick Ni sheet as a HER-active system with different numbers of non-doped graphene layers (GLs) and N-doped graphene layers (NGLs) (Fig. 1a and Supplementary Fig. 1). The graphene layers were prepared by a conventional chemical vapour deposition (CVD) method^16^ (see the Supplementary methods for details).

The crystallinities of graphene, various layer numbers, and degrees of structural defects were characterized by transmission electron microscopy (TEM), Raman spectroscopy, and X-ray photoelectron spectroscopy (XPS)^17^. The atomic structures of GLs and NGLs with various layer numbers were observed using high-resolution transmission electron microscopy (HRTEM). The HRTEM image of the monolayer GL (1GL) showed a honeycomb structure comprising six carbon atoms with six-fold symmetry spots assigned to monolayer graphene (Fig. 1b). The bilayer and trilayer GL (2GLs and 3GLs) possessed randomly orientated six-fold symmetry spots; i.e. 12 spots for the 2GLs and 18 spots for the 3GLs (Supplementary Fig. 2a, b)^18,19^. The HRTEM images of NGLs showed various types of topological defect (e.g. single and double vacancies) induced by N-doping (Inset of Fig. 1c, and Supplementary Fig. 2c, d)^20^. Raman images of 1GL and 1NGL on a window-attached Si_3_N_4_ chip (window size: 10 × 10 μm) indicated high *2D*-band to *G*-band (*I*_2D_/*I*_G_) intensity ratios of 3.8–4.9, suggesting that the monolayer graphene is highly crystalline^21,22^ (Fig. 1d–g, Supplementary Figs. 3–9, and Supplementary Table 1). Raman images of the GLs showed low *D*-band-to-*G*-band (*I*_D_/*I*_G_) intensity ratios of 0.05–0.08. In contrast, the NGLs exhibited relatively high *I*_D_/*I*_G_ ratios (0.7–1.4), which illustrates that N-doping induced many defects. We interrogated the chemical binding state of GLs and NGLs through XPS (Supplementary Fig. 10). The C 1*s* XPS spectra of the GLs and the NGLs confirmed the high quality of the graphene without evident oxidation. The N 1*s* XPS spectrum revealed that the N-dopants exist in pyridinic (0.18 at.%), graphitic (0.76 at.%), and oxidic structures (0.36 at.%) in the NGLs^23,24^. We observed a very tiny amount of residual Cu metal (≤0.01 at.%) (Supplementary Fig. 11).

**Fig. 2:**
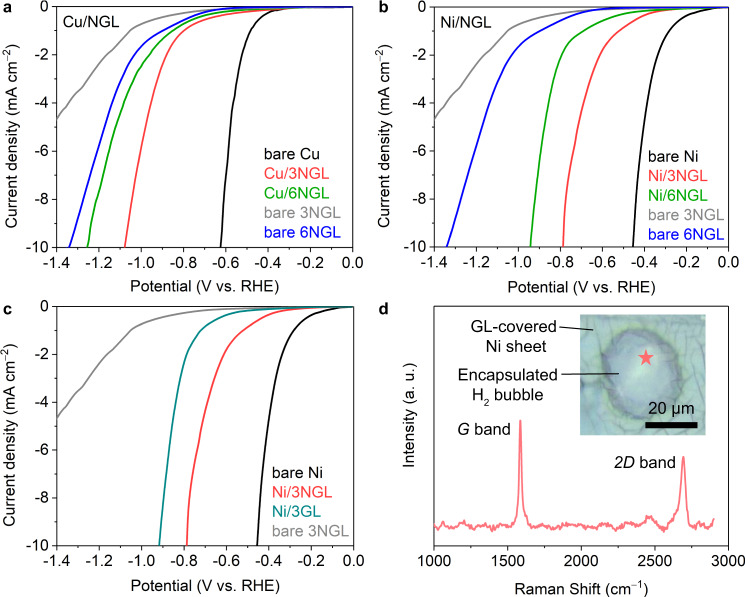
HER activities of the graphene-covered Cu and Ni sheets. HER polarization curves of the graphene-covered (**a**) Cu and (**b**, **c**) Ni sheets. **d** Raman spectra of the 3GL on a H_2_ bubble after the HER test. Inset of (**d**): the optical photo shows that a H_2_ bubble was generated between the Ni sheet and the graphene layers. The Raman measurement was obtained at the location indicated by the red star.

We subsequently transferred the graphene layers onto the Cu and Ni sheets with trilayer NGL and hexalayer NGL (abbreviated as Cu/3NGL, Cu/6NGL, Ni/3NGL, and Ni/6NGL, respectively). These graphene-covered Ni and Cu sheets were sandwiched between Nafion films. We measured the HER activities of the Cu/3–6NGL and Ni/3–6NGL samples in 0.5 M H_2_SO_4_ using a three-electrode system and compared them to those of the bare 3–6NGL samples (without metal substrates) as well as the bare Cu and Ni sheets (without graphene-covering). The HER polarization curves of the Cu/3–6NGL samples showed that the overpotential (*η*_10_) at the current density 10 mA cm^−2^, normalized by the electrode surface area, increased with an increasing number of NGLs (Fig. 2a). The *η*_10_ value of the Cu/3NGL sample is 36.0% lower than that of the bare 3NGL sample, which can be attributed to charge transfer from the underlying metal substrate to the outermost graphene layers^13^. As previously reported, the charge transfer effect becomes negligible beyond three graphene-covering layers^8,13^. Indeed, the Cu/6NGL exhibited a 6.5% lower *η*_10_ value compared to the bare 6NGL. This result suggests that the charge transfer effect determines the HER activity of Cu/NGL samples (Cu is HER-less-active).

We further measured the HER activities of the Ni/NGL samples under the same experimental conditions (Fig. 2b). If the entire HER occurs on the outermost graphene layers and the charge transfer solely determines the HER activity, the *η*_10_ values of the Cu/NGL and Ni/NGL samples should be identical regardless of the catalytic activity of the metal substrates. However, the observed *η*_10_ values of the Ni/3NGL and Ni/6NGL samples were 53.3% and 29.7% lower than those of the bare 3NGL and 6NGL samples. The reductions in the *η*_10_ values due to the Ni substrate (53.3% for the 3NGL and 29.7% for the 6 NGL) are much larger than the reductions due to the Cu substrate (36.0% for the 3NGL and 6.5% for the 6 NGL, Supplementary Table 2). This is also the case for the GL samples (Fig. 2c). The dramatic decrease in the *η*_10_ values for the Ni/NGL samples cannot be solely explained by the effects of charge transfer. These differences in catalytic activity between the HER-less-active Cu and the HER-highly-active Ni suggest that the HER occurs not only on the graphene surface but also on the underlying metal surface.

To confirm that HER occurs on the covered metal surface, we applied an electrode potential to the Ni/3GL sample with a slow scan rate (0.01 mV/s) and observed the generation of H_2_ bubbles at the interface between the Ni sheet and the graphene layers (Fig. 2d). Raman spectra measured at the spot marked by the red star verified that the generation of the bubbles occurred at the interface and that there were no obvious cracks in the graphene layers (inset of Fig. 2d). Overall, our results strongly suggest that the protons penetrate through the graphene layers and reach the underlying metal substrate, triggering HER.

### Electrochemical proton-penetration experiments

**Fig. 3:**
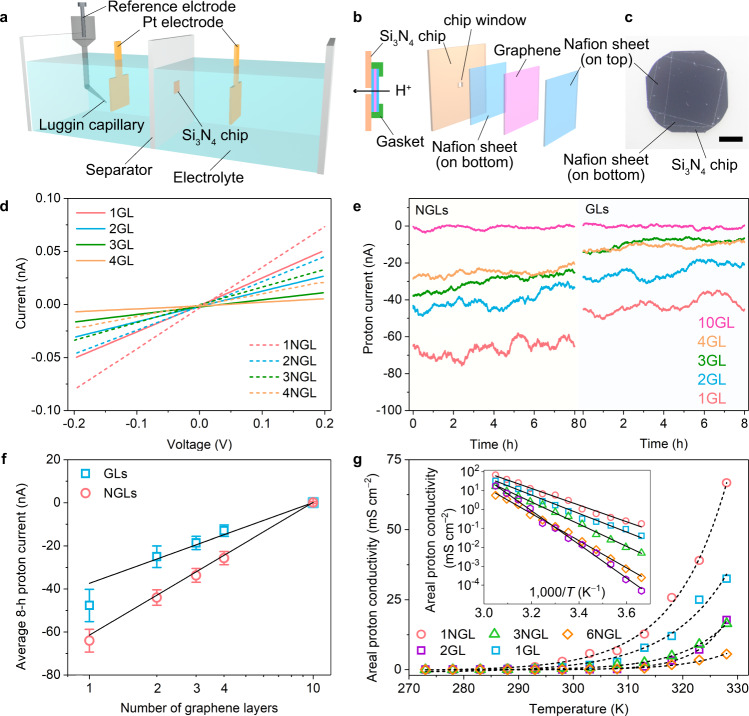
H-type cell fabrication and electrochemical data for proton penetration through graphene layers. **a** Schematic of the H-type cell. **b** Placement of a stacked Nafion/graphene/Nafion sandwich membrane over the window area of a Si_3_N_4_ chip. Nafion acts as a protective sheet for graphene layers with high proton conductivity, chemical/electrochemical stability, and extremely low electron conductivity^43^. All gaps and points of connection were sealed by an acid-stable, ion-impenetrable gasket to ensure that protons could only pass through the graphene on the window area. **c** Optical photographic image of the Si_3_N_4_ chip with an attached Nafion/graphene/Nafion sandwich. **d** *I*–*V* characteristics of the proton penetration through non- and N-doped graphene membranes with one to four layers. The solid and dashed curves represent the proton currents observed through GLs and NGLs, respectively. **e** Proton currents collected by CA at a cathode potential of −20 mV vs. RHE through the GLs and the NGLs. **f** Average proton currents collected during 8 h of CA testing. Error bars show the fluctuations in the measured signals. **g** Temperature dependences of proton conductivity for 1GL, 2GL, 1NGL, 3NGL, and 6NGL. Inset of (**g**): log *σ* as a function of *T*^−1^. The solid and dashed curves represent the fits to the experimental results.

Above, we confirmed that the proton penetrates through the graphene layers. To study the mechanism of the proton penetration, GLs and NGLs were employed as separating membranes in an H-type cell (Fig. 3a). Monolayer GL and NGL were stacked layer-by-layer and covered with Nafion (proton conductivity: ~90 mS cm^−1^ at 24 °C^25^) on both sides. The Nafion/graphene layers/Nafion sandwich membrane was placed on the same Si_3_N_4_ chip used for the Raman measurements to isolate the anode and cathode chambers of the H-type cell (Fig. 3a–c).

We subsequently examined the proton penetration pathway through graphene by measuring the open circuit potential (*E*_OC_) between the two chambers that were physically separated by graphene in the H-type cell (Supplementary Fig. 12). The measured *E*_OC_ value remained constant at 71 mV for 6 h at a pH of 1.74 in the anode chamber and a pH of 0.50 in the cathode chamber. The experimental value of 71 mV is close to the theoretical *E*_OC_ value of 73 mV calculated by the Nernst equation (see Supplementary methods), which confirms that a proton penetration pathway exists and that the electrolyte does not leak. We explored the degrees of the proton penetration through the GLs and NGLs from the current–voltage (*I*–*V*) characteristics in a two-electrode system. The proton current through the graphene layers was proportional to the bias voltage in the +200 to −200 mV range (Fig. 3d and Supplementary Fig. 13). The measured proton currents varied linearly with the bias voltage and their slopes decreased gradually with an increasing number of graphene layers, suggestive of an increasing resistance to proton penetration. The proton conductivities through 1GL and 1NGL were 0.27 and 0.44 mS cm^−2^, respectively (Supplementary Figs. 14 and 15, and Supplementary Table 3), which were much smaller than the proton conductivity through a Nafion separator membrane (43.0 mS cm^−2^). This manifests that the proton-penetration resistance arises predominantly from the graphene layers rather than the Nafion. The electrochemical resistance of proton penetration through the graphene layers was further investigated by electrochemical impedance spectroscopy. The results also confirmed the correlation of the proton-penetration resistance and the layer number of graphene (details in Supplementary discussion 1, Supplementary Fig. 16, and Supplementary Table 4).

Time dependence of the proton penetration at a constant electrode potential in a three-electrode system was investigated by chronoamperometry (CA). The proton currents, which are defined as the limited current of HER by proton diffusion, through the NGLs were observed to be 1.5 times greater than those through the GLs (Fig. 3e, f, Supplementary Figs. 17–19, and Supplementary Table 5). Eventually, we observed almost zero current for the 10-layer graphene. This penetration behaviour was further investigated by isotope experiments (Supplementary Figs. 20 and 21, and Supplementary Tables 6 and 7). The proton current through 1GL was 1.4–1.7 times larger than the deuteron current. The difference in the current between proton and deuteron suggests the isotope effect is present (Supplementary Fig. 20).

We experimentally examined the energy barriers for proton penetration through graphene membranes by measuring the proton conductivity as a function of the temperature (Fig. 3g)^14^. An Arrhenius-type-fit of the data provides the energy barriers of 0.95 ± 0.03 and 0.87 ± 0.03 eV for 1GL and 1NGL, respectively. In addition, the energy barriers for GLs are almost proportional to the number of GLs (2GL: 1.76 ± 0.04 eV) while the energy barriers for NGLs are not simply proportional to the number of NGLs (3NGL: 1.17 ± 0.02 eV, 6 NGL: 1.42 ± 0.02 eV). These results indicate that the nitrogen dopant-induced defects on the graphene lattice accelerate the proton penetration^26^. Moreover, the *I*–*V* curves of 6NGL demonstrated a non-linear behaviour at a wide voltage range from −3.5 to +3.0 V (Supplementary Fig. 22). The “apparent” activation energy at a high voltage range from −3.5 to −3.0 V was estimated as 0.43 ± 0.02 eV, which is much lower than the 1.42 ± 0.02 eV energy barrier estimated at a low voltage range from −0.2 to 0.0 V. This manifests that the activation energy can be drastically lowered due to the applied voltage; the protons overcome the activation energy utilizing an applied electric field. In fact, the simulation shows that the high applied voltage primarily affects the electric potential near the graphene interface, with a thickness of sub-nanometre (Supplementary Fig. 23). Take these results together, we conclude that protons can penetrate through the graphene layers under an applied voltage, and this penetration is enhanced by N-doping and by reducing the number of graphene layers.

### Universal relationship between the proton current and HER activity

**Fig. 4:**
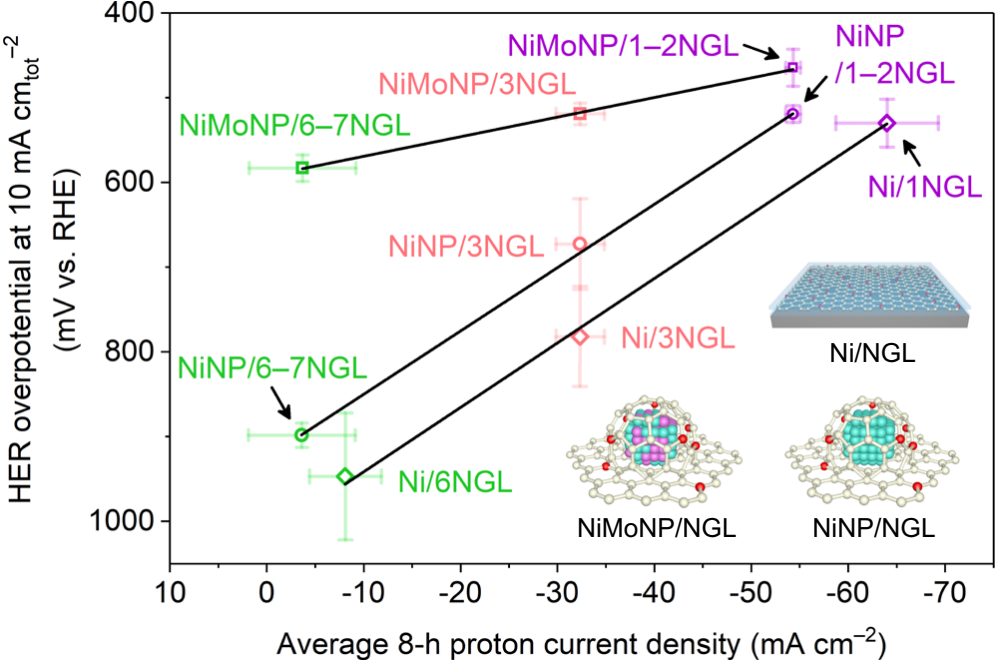
Universal correlation between the HER activity and the proton penetration through the graphene layers. Graphene-covered Ni sheet, NiNP, and NiMoNP with various numbers of graphene layers were employed. Colour code: carbon (white), nitrogen (red), nickel atom (cyan), nickel sheet (grey) and molybdenum (purple). Error bars show the fluctuations in the measured signals. The current density was normalized by the window area for the *X*-axis and by the total surface area of the catalysts for the *Y*-axis. Note: a 10 nA proton current is equivalent to a 10 mA cm^−2^ proton current density.

We correlated the proton penetration with the HER activities of the graphene-covered Ni sheet, Ni nanoparticle (NiNP), and NiMo alloy nanoparticle (NiMoNP) catalysts (Supplementary method, Supplementary discussion 2–6, Supplementary Figs. 24–35, and Supplementary Table 8). We plotted the average proton current density (mA cm^−2^) from 8 h of CA testing as a function of the overpotential (*η*_10-tot_) at the current density of 10 mA cm_tot_^−2^, normalized by the total surface area (i.e. the Brunauer-Emmett-Teller (BET) surface area of the catalyst), and the current density at −600 mV vs. RHE for fair catalytic ability comparisons (Fig. 4 and Supplementary Fig. 32). Surprisingly, excellent correlations between the proton penetration through the graphene layers and the catalytic activity were observed. These results reveal that the catalytic activity of the graphene-covered non-noble metal catalysts is governed by proton penetration through the graphene layers, regardless of substrate morphology (sheet or nanoparticle) or composition (metal or alloy). Moreover, similar but shifted slopes were observed for the Ni sheet and NiNP samples, while the NiMoNP sample exhibited a shallower slope. This can be attributed to the number of active sites for the same substrate composition but with different morphology (Ni sheet vs. NiNP), and to the catalytic activity of the same substrate morphology but with different compositions (NiNP vs. NiMoNP). Thus, the HER overpotential of the graphene-covered non-noble metal catalysts reflects the HER activity of the underlying metal catalysts.

### Unveiling proton-penetration pathways using density functional theory

**Fig. 5:**
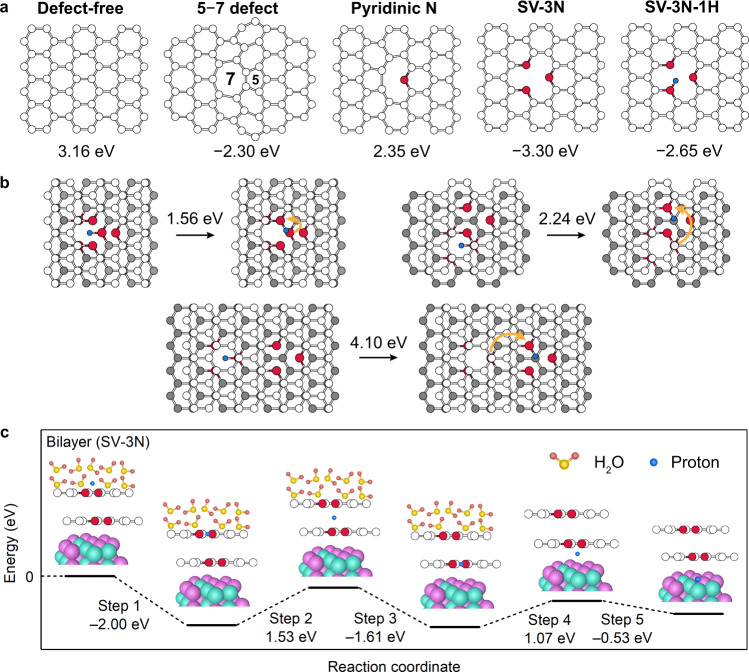
DFT calculations. **a** Atomic models of various types of graphene lattice. The numbers under the graphene lattices are the calculated energy barriers for proton penetration through the lattices. A positive or negative value indicates that the shape of the potential energy surface involves a peak or a trough (details in Supplementary Fig. 36). **b** Energy barriers for proton hopping between bilayer SV-3N graphene lattices (the first layer is white and the second layer is grey). Orange arrows indicate the hopping direction. **c** Energy diagram for proton penetration through a bilayer SV-3N graphene lattice. The proton was adsorbed on a NiMo nanoparticle after penetrating through the graphene-covering bilayer. Colour code: carbon (white), nitrogen (red), proton (blue), nickel (cyan), molybdenum (purple), oxygen (light yellow), and hydrogen (dark yellow).

Questions that arise here include: how do NGLs enhance proton penetration compared to GLs, and how does a proton penetrate through multiple graphene layers? To address these questions, we computed the energy barriers for proton penetration through a defect-free 1GL lattice, a 1GL lattice with a 5–7 defect, and several types of NGL lattices using the nudged elastic band (NEB) method. Our calculations reveal energy barriers of 3.16 eV and 2.30 eV for a 1GL lattice with no defects and a 1GL lattice with a 5–7 defect, respectively (Fig. 5a, Supplementary Figs. 36–38, and Supplementary Tables 9 and 10). The barrier is reduced because the proton can more easily penetrate through the seven-membered carbon rings in the defect-containing graphene than through the six-membered carbon rings in the defect-free graphene.

Pyridinic N-dopants also lowers the energy barrier for proton penetration. The pyridinic NGL lattice requires an energy barrier of 2.35 eV for proton penetration through its six-membered carbon rings, which is 0.81 eV lower than that of the defect-free GL lattice (Fig. 5a) and is ascribable to the electronegativity of the N-dopant (Supplementary Fig. 39). Proton penetration through NGL lattices containing a single vacancy (SV) and three N-dopants (SV-3N) requires more energy than required for penetration through a defect-free GL lattice, because of stronger proton/N binding (i.e. the protons tend to be trapped by N-dopants). However, the adsorption of H atoms at the N-dopants reduces the barrier of proton penetration by contributing to proton repulsion. For example, the SV-3N lattice with one adsorbed H atom (SV-3N-1H) exhibits a smaller energy barrier (2.65 eV) compared to that for the SV-3N lattice (3.30 eV). The energy barrier of the SV-3N lattice decreases from 3.30 eV to 1.93 eV in the presence of H_2_O molecules (Supplementary Fig. 38). Our results suggest that 5–7 topological defects and pyridinic N-dopants reduce the energy barrier for proton-penetration, compared with those of the defect-free graphene.

To determine how a proton penetrates through multiple graphene layers, we calculated the energy barriers for proton-hopping between the bilayer SV-3N graphene lattices (Fig. 5b). As discussed above, a proton can be trapped by a pyridinic N-dopant. Such a trapped/adsorbed proton can hop to another pyridinic N-dopant in another graphene layer. The energy barrier for proton hopping depends highly on the relative positions of the pyridinic N-dopants in the two layers (1.56–4.10 eV). The energy barrier can be as low as 1.56 eV when the two N-dopants are close to each other; this barrier is smaller than that for pyridinic N-doped monolayer graphene (Fig. 5a). Our results show that a proton can penetrate through multiple graphene layers by hopping between N-dopants.

The pathway for proton penetration through multiple graphene layers to the non-noble metal surface in the presence of H_2_O molecules is summarized in Fig. 5c and Supplementary Fig. 40. A proton is adsorbed on an N-dopant of the topmost layer (Step 1), and then the proton intercalates into the bilayer lattice (Step 2). As in Step 1, the proton is adsorbed onto the inner layer (Step 3). The proton finally reaches the NiMo surface (Steps 4 and 5; Supplementary Fig. 41) where the protons undergo H_2_ evolution. According to this sequence of events, adsorption on the NiMo substrate is energetically preferable to complete desorption following penetration (i.e. returning to the initial state requires an energy barrier of 2.00 eV).

### Unveiling the ejection mechanism of the generated hydrogen molecules

We explored the ejection mechanism of generated H_2_ molecules through graphene lattices (Supplementary Figs. 42–46). The reported studies^9–11^ suggested that generated H_2_ bubbles cannot penetrate through a defect-free GL^27^. Indeed, we observed that the generated hydrogen bubbles were grown in the confined region sandwiched by the Ni sheet and the defect-less GLs at the low potential range from 0.0 to −0.2 V (vs. RHE) (Supplementary Fig. 45b). Moreover, because of the growth of the H_2_ bubble, the crystallinity of the bubble encapsulating graphene was lowered (Supplementary Fig. 46b). Meanwhile, the defects and nanopores allowed the H_2_ molecule to move from the Ni surface to the outside of the graphene layers. These experimental results were further confirmed by the simulations. Our simulation data show that an H_2_ molecule must overcome an energy barrier of 4.6–5.4 eV to pass through defect-free graphene or graphene having a small topological defect (e.g. 5–7 or 5–8–5 defect), but it must only overcome an energy barrier of <1.0 eV when graphene has nanopores (diameter: 2.5–5.8 Å) (Supplementary Table 11 and Supplementary discussion 7). Thus, the nanopores on graphene layers promote the ejection of generated H_2_ molecules. Note that the charge-neutral H_2_ penetrates more easily through defects/nanopores than the excess number of protons and larger-sized hydrated cations^28^ at the cathodes. In other words, the hydrated ions are blocked from penetrating the graphene because additional energy is required for rearranging the solvation structure of ions and surrounding water molecules.

Nitrogen dopants and structural defects play an important role in H_2_ ejection. In the case of graphene/Ni sheet case, H_2_ bubbles were generated on the NGL-covered Ni sheet without the formation of cracks in N-doped graphene layers, which was opposite to the case of the GL-covered Ni sheet (Supplementary discussion 8 and Supplementary Figs. 44–46). These differences between the NGL and GL can be attributed to the efficient ejection of H_2_ through dopant-/defect-rich regions in the NGL. In the case of graphene-covered metal/alloy nanoparticle morphology, a high defect density, e.g. 7-, 8- or larger-membered carbon rings, on the highly curved graphene lattice is geometrically required for constructing the curved morphology to fit the graphene lattice with the nanoparticle surface^29,30^, which contributes to the H_2_ molecule ejection.

## Discussion

**Fig. 6:**
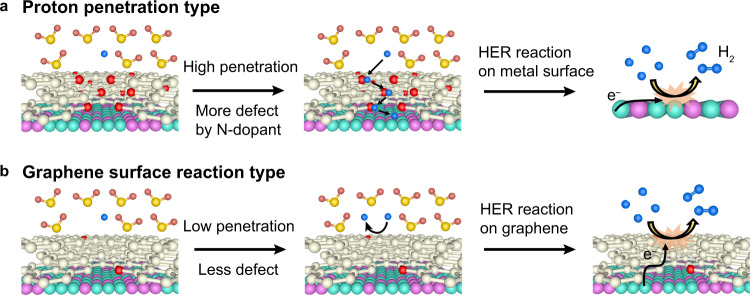
Schematic HER mechanisms for graphene-covered non-noble metal catalysts. **a** Proton-penetration-type mechanism. Protons penetrate through the graphene covering layers, and H_2_ evolution occurs on the surface of the underlying non-noble metal substrate. **b** Graphene-surface-reaction-type mechanism. The H_2_ evolution occurs on the outermost graphene layer by utilizing charge transfer from the underlying non-noble metal substrate. Colour code: carbon (white), nitrogen (red), proton (blue), nickel (cyan), molybdenum (purple), oxygen (light yellow), and hydrogen (dark yellow).

We explored the impact of proton penetration on catalytic activity by combining HER catalytic-activity experiments of graphene-covered non-noble metal catalysts, proton-penetration characterizations, and DFT-calculated energy barriers for the penetration. The linear relationship between the required HER overpotential in the graphene-covered non-noble metal catalysts and the proton-penetration current through the graphene layers (Fig. 4) strongly suggests that catalytic activity is dominated by proton penetration through the graphene layers. Our DFT calculations revealed that NGLs exhibit enhanced proton penetration compared with GLs as a result of graphene-lattice deformation through N-doping, as well as the electronegativity of the N-atom dopant (Supplementary Fig. 39), which is in agreement with the experimental results (Fig. 1c). The overall process, which can be universally applied to various graphene-covered non-noble metal catalysts, is summarized in Fig. 6a.

It has been reported that the catalytic HER reaction arises from the charge transfer effect occurring on the outermost graphene layer (Fig. 6b)^31,32^. This raises the question of whether the charge transfer effect or the proton-penetration process is dominant for the current HER reaction. To address this, we investigated the HER activities of the graphene-covered Ni and Cu sheets. If the catalytic reaction that occurs on the graphene surface can compete with the underlying metal’s catalytic process achieved by proton penetration, the required overpotentials in the Ni and Cu systems are comparable. However, a much larger overpotential in the Cu system is required in comparison to the Ni system (Fig. 2a, b), which reveals that the process depicted in Fig. 6b is a minor contributor due to the lower catalytic activity of graphene than that of the underlying metal, thus the process shown in Fig. 6a dominates for graphene-covered non-noble metal catalysts.

Graphene-covering technology is also important for ensuring the catalyst lifetimes of non-noble metal catalysts. The monolayer graphene-covered NiMoNP sample dissolves during 1000 CV cycles (Supplementary Fig. 30), whereas the catalyst with multilayers of graphene endures 1000 CV cycles (Supplementary Fig. 31). These results confirm that protons reach the underlying metal surface and dissolve the non-noble metal catalysts. Moreover, corrosion resistance (in the form of high energy barriers) gradually increases and the degree of proton penetration decreases with increasing numbers of graphene layers; hence, HER activity is sacrificed for an improved catalyst lifetime (i.e. to protect non-noble metals in the acidic medium) resulting from the low quantity of penetrating protons.

In conclusion, our study revealed the fundamental HER mechanism of graphene-covered non-noble metal catalysts in acidic electrolytes; our results demonstrated that layer number, N-doping, and structural defects in the covering graphene govern the degree of proton penetration and, consequently, the catalytic HER activity. These results indicate that engineering the thickness, doping level, and defect density of the graphene would allow for optimization of the HER activity of non-noble metal catalysts, while simultaneously suppressing their corrosion. Techniques that optimize these factors in the covering graphene would be crucial in improving the usability of non-noble metal catalysts not only for the HER in acidic media, but also for various circumstances that require the corrosion-proofing of non-noble metals.

## Methods

### Synthesis of monolayer graphene for graphene-covered samples and separating membranes

Monolayer GL and NGL were grown on a Cu foil (99.8%, 25 µm thickness, Alfa Aesar) via a conventional CVD method using CH_4_ (99.99%) and pyridine (Aldrich, 99.8%, anhydrous) as carbon and nitrogen sources^16,24^. The Cu foil was loaded on a corundum boat and inserted into the centre of a quartz tube (*φ*30 × *φ*27 × 1000 mm) in a furnace. The tube was heated at 1000 °C for 1 h under an atmosphere of H_2_ (100 sccm, 99.99%) and Ar (200 sccm, 99.99%). Then, GL was grown with an additional flow of CH_4_ (20 sccm, 99.995%) for 30 min, while NGL was grown with an additional flow of CH_4_ (20 sccm) and pyridine (0.5 m bar). The as-synthesized graphene on Cu foils after Cu dissolution in 0.25 M Fe(NO_3_)_3_ solution was manually stacked to prepare graphene-covered Cu and Ni sheets and graphene separating membranes (details in Supplementary method and Supplementary Figs. 1, 3).

### Characterization of samples

Morphology and microstructure of as-synthesized graphene were characterized using a scanning electron microscope (SEM, HITACHI S-4300), transmission electron microscopes (TEM, JEOL JEM-2100 F and JEM-ARM200F), and equipped energy dispersive spectroscopy (EDS, SDD Type, Detection surface area 30 mm^2^, Solid angle 0.26 sr). Raman spectra were performed using a Renishaw InVia Reflex with an incident wavelength of 532 nm. X-ray diffraction (XRD) was carried out using a D2 PHASER (Cu Kα1 radiation; λ = 1.5406 Å, BRUKER). Surface chemical states were studied by X-ray photoelectron spectroscopy (XPS, AXIS ultra DLD, Shimazu) with an Al Ka and X-ray monochromator.

### Electrochemical measurements

An electrochemical workstation (Biologic, VSP-300) was used for all electrochemical measurements. Catalytic activities of graphene-covered Cu/Ni sheets and Ni/NiMo nanoparticles samples were performed in a three-electrode system. As-prepared graphene-covered catalysts, a graphite rod, an Ag/AgCl electrode, and Ar-saturated 0.5 M H_2_SO_4_ served as the working electrode, counter electrode, reference electrode, and electrolyte, respectively. The potential was calculated with respect to RHE using the equation: *E*(RHE) = *E*(Ag/AgCl) + 0.0591 × pH + 0.197. The pH value of the electrolyte was recorded as 0.5–0.55. The Ag/AgCl reference electrode was calibrated using a Pt wire as the working electrode. The polarization curves were obtained using a sweep rate of 5.0 mV s^−1^. The electrode potential was automatically *iR*-compensated with the ohmic resistance.

### Proton penetration measurements

The *I*–*V* characteristics and electrochemical impedance spectra were performed in a two-electrode system. The schematic of the homemade H-type cell for electrochemical measurements of proton penetration was shown in Fig. 3a. A glass separator with a window-attached Si_3_N_4_ chip (window size: 10 × 10 μm) fixed in the centre was used to isolate the anode and cathode chambers filled with 0.5 M H_2_SO_4_. The distance between the electrode and separator was 1.0 cm. The size of the Pt plate electrode was 1.0 × 1.0 cm. Electrochemical impedance spectra were recorded with the frequency ranging from 10^6^ to 100 Hz. The CA measurements were carried out in a three-electrode system, in which an Ag/AgCl electrode equipped with a salt bridge was used as the reference electrode. Temperature dependence of proton conductivity experiments for graphene layers were performed over a temperature range of 273–328 K to avoid water freezing and membrane damage due to thermal expansion.

### DFT calculation

The DFT calculations were performed via the CP2K program^33^. The Becke-Lee-Yang-Parr (BLYP)^34,35^ exchange-correlation functional was used. We employed double-zeta valence plus polarization (DZVP) basis sets. The core electrons were described by the Goedecker-Teter-Hutter pseudopotential^36^. The real-space density cut-off was set to 320 Ry. The van der Waals correction was included via Grimme’s D3 method^37^. The simulation cell lengths in the x-, y-, and z-directions were 25.56, 24.595, and 50 Å^38,39^, respectively. The C–C bond lengths of pristine graphene are 1.42 Å. The 5–7 defect was introduced according to the reported literature^40^. The nudged elastic band method^41^ was used with three intermediate states. The calculated barrier heights are independent of calculation methods and consistent with previous reports (Supplementary Tables 9–11). The atomic charges were calculated using the iterative Hirshfeld scheme (Hirshfeld-I)^42^.

## Supplementary information

Supplementary Information

Peer Review File

## Data Availability

The data that support the findings of this study are available from the corresponding authors upon reasonable request.
