# Supplementary material for: Catalytic activity of graphene-covered non-noble metals governed by proton penetration in electrochemical hydrogen evolution reaction
Source: Nat Commun. 2021 Jan 8;12:203. doi: 10.1038/s41467-020-20503-7 (PMC7794435; doi:10.1038/s41467-020-20503-7)
Supplement: Supplementary file 1 — Supplementary Information [file 41467_2020_20503_MOESM1_ESM.pdf]

Supplementary Information

Catalytic Activity of Graphene-Covered Non-Noble Metals Governed by Proton Penetration in Electrochemical Hydrogen Evolution Reaction

Kailong Hu[1], *Tatsuhiko Ohto*[2,*], *Yuki Nagata*[3], *Mitsuru Wakisaka*[4,6], *Yoshitaka Aoki*[5,6], *Jun-ichi Fujita*[1], *Yoshikazu Ito*[1,6,*]

[1]Institute of Applied Physics, Graduate School of Pure and Applied Sciences, University of Tsukuba, Tsukuba 305-8573, Japan.
[2]Graduate School of Engineering Science, Osaka University, 1-3 Machikaneyama, Toyonaka 560-8531, Japan.
[3]Max Planck Institute for Polymer Research, Ackermannweg 10, 55128 Mainz, Germany.
[4]Graduate School of Engineering, Toyama Prefectural University, 5180 Kurokawa, Imizu, Toyama 939-0398, Japan.
[5]Faculty of Engineering, Hokkaido University, N13W8 Kita-ku, Sapporo, 060-8628 Japan.
[6]PRESTO, Japan Science and Technology Agency, Saitama 332-0012, Japan.

E-mail: ito.yoshikazu.ga@u.tsukuba.ac.jp, ohto@molectronics.jp

Supplementary methods

Fabrication of graphene-covered Cu and Ni sheets. After the monolayer graphene growth on Cu foils by a standard chemical vapor deposition (CVD) process, O_2 plasma treatment was operated to remove the graphene on one side of Cu foil, and then Nafion sheet was coated on the other side through a spin coater. The Cu foils were dissolved by 0.25 M $Fe(NO_3)_3$ solution at 24 °C for 12 h, and then the ultrapure water (resistivity: 18.2 MΩ) was used to replace the $Fe(NO_3)_3$ solution to remove ions several times completely. After transferring a graphene sheet onto a Nafion-attached Cu or Ni sheet, the Nafion/monolayer graphene/Nafion on the Cu or Ni sheet was achieved. Finally, the as-prepared sheet was heated at 130 °C for 2 h and coated by an insulator liquid gasket (ThreeBond 1211) to prevent the contact with acidic electrolyte (Supplementary Fig. 1). Typical size of exposed reaction area was 2.0 × 2.0 mm. For preparing the Cu or Ni sheet with multilayer graphene covering, the process was as same as the steps shown in Supplementary Fig. 3.

Fabrication of a Si_3N_4 chip supported Nafion/graphene/Nafion membrane. After the monolayer graphene growth on Cu foils, O_2 plasma treatment was operated to remove the graphene on one side of Cu foils, and then Nafion sheet was coated on the other side with graphene through a spin coater. The Cu foils were dissolved in 0.25 M $Fe(NO_3)_3$ solution at 24 °C for 12 h, and then the ultrapure water (resistivity: 18.2 MΩ) was used to replace the $Fe(NO_3)_3$ solution to wash ions several times. After transferring a graphene sheet to a Si_3N_4 chip with an attached Nafion sheet on window area, the Nafion/monolayer graphene/Nafion membrane on a Si_3N_4 chip was achieved. Finally, the chip was heated at 130 °C for 2 h before using.

For the bilayer graphene membrane, one additional step was needed: transfer a Nafion protective monolayer graphene onto a Cu foil with monolayer graphene on one side (step 6 in Supplementary Fig. 3). The chip was heated at 130 °C for 2 h after graphene transfer every time. For preparing the membrane with graphene more than bilayer, the process from step 4 to 7 was simply repeated.

Synthesis of graphene-covered Ni nanoparticles. The 3D porous graphene substrate

was achieved by etching the NiMo substrate of graphene-covered NiMo alloy using 2.0 M HNO_3 solution mixed with isopropanol (volume ratio of HNO_3 to IPA was 4:1) at 80 °C. The deposition of NiO nanoparticles on 3D porous graphene was followed a reported literature with slight modifications[1]. 20 mL ultrapure water (Millipore, resistivity: 18.2 MΩ) contained 3D porous graphene, 20 mL ethanol, 4.5 g $Ni(NO_3)_2 \cdot 6H_2O$ (FUJIFILM Wako Pure Chemical Corp., 98%), and 0.7 g urea (FUJIFILM Wako Pure Chemical Corp., 99%) were added into a 50 mL Teflon-lined stainless steel autoclave, and then treated at 120 °C for 24 h in a muffle furnace. After cooling down to 24 °C, the as-prepared products were washed by ultrapure water and ethanol several times, and further dried at 120 °C for 12 h. 10.0 mg of 3D porous graphene supported NiO nanoparticles was loaded on a corundum boat and inserted into the center of a quartz tube (φ30 × φ27 × 1000 mm) in a furnace. Through annealing at 300 °C for 20 minutes under an atmosphere of H_2 (99.9999%, 100 sccm) and Ar (99.999%, 200 sccm), the deposited NiO nanoparticles were reduced to Ni nanoparticles. After the reduction, the furnace temperature increased to 700 °C for the N-doped graphene growth on Ni nanoparticles by a CVD method under a mixed atmosphere of H_2 (100 sccm)/Ar (200 sccm)/pyridine (1.0 mbar, Ardrich, 99.8%, anhydrous). The layer number of encapsulating graphene was adjusted through controlling the CVD time. For example, deposition times were 1.0, 4.0, and 10.0 s for 1−2, 3, and 6−7 graphene layers, respectively. The average layer numbers were 1.9, 3.2, and 6.1 for the above three deposition times. After the CVD process, the furnace was cooled to 24 °C by using a fan and the resulting samples were stored for characterizations and measurements.

Synthesis of graphene-covered NiMo alloy nanoparticles. The 3D porous graphene substrate was achieved by etching the NiMo substrate of graphene-covered NiMo alloy using 2.0 M HNO_3 solution mixed with isopropanol (volume ratio of HNO_3 to IPA is 4:1) at 80 °C. The 3D porous graphene was firstly immersed in 0.15 M $[Ni(NH_3)_6]MoO_4$ solution, which was synthesized by mixing 0.3 M $NiMoO_4$ solution with $NH_3 \cdot H_2O$ (FUJIFILM Wako Pure Chemical Corporation, 28% in H_2O) for 12 h, and then washed by 2-proponal several times. The resulting 3D porous graphene with the Ni and Mo oxidized compounds was dried under vacuum at 40 °C for 5 h to remove NH_3. After that, the compounds were deposited on the 3D porous graphene substrate. The prepared sample

was loaded on a corundum boat and inserted into the center of a quartz tube ($\varphi 30 \times \varphi 27 \times 1000$ mm) in a furnace. Through annealing at 950 °C for 10 minutes under an atmosphere of H_2 (99.9999%, 100 sccm) and Ar (99.999%, 200 sccm), the deposited compounds were reduced to NiMo alloy nanoparticles. Then, the furnace temperature decreased to 700 °C for the subsequent graphene growth on NiMo nanoparticles by a CVD method. The non-doped graphene and N-doped graphene were grown at 700 °C under a mixed atmosphere of H_2 (100 sccm)/Ar (200 sccm)/benzene (1.0 mbar, Ardrich, 99.8%, anhydrous) and H_2 (100 sccm)/Ar (200 sccm)/pyridine (1.0 mbar, Ardrich, 99.8%, anhydrous), respectively. The number of graphene covering layers was adjusted through controlling the CVD time. For example, deposition times were 1.0, 4.0, and 10.0 s for 1−2, 3, and 6−7 N-doped graphene layers, respectively. The average layer numbers were 1.7, 2.9, and 6.4 for the above three deposition times. After the CVD process, the furnace was cooled to 24 °C by using a fan and the resulting samples were stored for characterizations and measurements.

Synthesis of non-doped graphene (GL)-covered and N-doped graphene (NGL)-covered Ni sheets for generated H_2 bubbles observation. The Ni sheets were loaded on a corundum plate and inserted into the center of a quartz tube ($\varphi 30 \times \varphi 27 \times 1000$ mm) in a furnace. Through pre-annealing at 1000 °C for 30 minutes under an atmosphere of H_2 (99.9999%, 100 sccm) and Ar (99.999%, 200 sccm), the covering GL on Ni sheet was synthesized through introducing CH_4 flow (20 sccm, 99.995%) for 30 minutes at 1000 °C, while NGL was synthesized with an additional flow of pyridine (0.5 m bar) at 800 °C. Finally, the as-prepared Ni sheets were fully coated by an insulator liquid gasket (ThreeBond 1211) to prevent the contact with acidic electrolyte. The size of exposed reaction area was 1.0 × 1.0 cm.

Operation check of the H-type cell through the Nernst equation

According to the Nernst equation,

$$E_{\mathrm{H^+/H_2}} = E^{\mathrm{o}} - \frac{RT}{nF}\ln\left(\frac{P_{\mathrm{H_2}}}{[\mathrm{H^+}]^2}\right),$$

where E was the cell potential, E^{o} was the standard cell potential, R was the universal gas constant, T was the temperature in kelvins, n was the number of transferred electrons in the half reaction, F was the Faraday constant, $P_{\mathrm{H_2}}$ was the H_2 pressure, and $[\mathrm{H}^+]$ was the concentration of H^+ ions when the activity coefficient was 1.

When $P_{\mathrm{H_2}}$= 1 atm, and T = 298 K,

$$E_{\mathrm{H^+/H_2}} = 0 - \frac{8.314\ \mathrm{J\ K^{-1}} \times 298\ \mathrm{K}}{2 \times 96485\ \mathrm{J\ V^{-1}}} \ln\left(\frac{1}{[\mathrm{H}^+]^2}\right) = 0.059 \times \log[\mathrm{H}^+].$$

Combined with $\mathrm{pH} = -\log[\mathrm{H}^+]$,

$$E_{\mathrm{H^+/H_2}} = (-0.059 \times \mathrm{pH})\ \mathrm{V}.$$

Based on the experimentally measured pH values of two chambers (1.74 and 0.5) in Supplementary Fig. 12a, the potential difference should be,

$$E = |-0.059 \times (1.74 - 0.5)| = 0.073\ \mathrm{V} = 73\ \mathrm{mV}.$$

Deuterium ion (D^+) penetration experiments. The configuration of the experimental cell and the setting of graphene membrane were shown in Supplementary Fig. 20. 0.5 M H_2SO_4 (FUJIFILM Wako Pure Chemical Corporation) and 0.5 M D_2SO_4 (SIGMA-ALDRICH, 96−98 wt% in D_2O, 99.5 at% for D) electrolytes were employed in two separated chambers. The electrolyte in cathode side was collected for detection of deuterium ions transferred from anode side after an 8 h CA measurement at a cathode potential of −20 mV vs. RHE. The deuterium ions were detected by a gas chromatography–mass spectrometry (GC-MS, JMS-Q1500GC, JEOL). The experimental conditions were 70 eV ionization voltage, 2 μA ionization current with the scan rage of m/z 10−150 and 1000 V detector gain. 10 μL of liquid sample was directly spread inside the ionization chamber and gradually increased the temperature.

Electrochemical measurements for graphene-covered Ni nanoparticles and NiMo alloy nanoparticles. HER polarization curves and CA measurements were performed in

a three-electrode system. As-synthesized graphene-covered Ni nanoparticles (NiNP) and NiMo alloy nanoparticles (NiMoNP) samples deposited on a glassy carbon rotating disk electrode (RDE, diameter: 5 mm), a graphite rod, an Ag/AgCl electrode, and Ar-saturated 0.5 M H_2SO_4 solution served as the working electrode, counter electrode, reference electrode, and electrolyte, respectively. The potential was calculated with respect to RHE using the equation: E(RHE) = E(Ag/AgCl) + 0.0591 × pH + 0.197. The pH value of electrolyte (0.5−0.55) was recorded and the Ag/AgCl reference electrode was calibrated before tests. The polarization curves were obtained using a sweep rate of 5.0 mV s^{-1}. The sample loading amount was 5.0 mg. The RDE rotation speed was 1600 rpm to remove generated hydrogen bubbles. The electrode potential was automatically *iR*-compensated with the ohmic resistance.

DFT calculations. We also performed DFT calculations to estimate the energy barrier for hopping of proton from graphene to the NiMo surface by using the VASP[2] code. We used the projected augmented wave (PAW) method[3] and the Perdew-Burke-Ernzerhof (PBE) functional[4]. The plane wave energy cutoff was set to 400 Ry. The dispersion correction was included using the Grimme's D3 (BJ) method[5, 6].

The NiMo surface covered by a graphene with SV-3N defect was prepared based on our previous paper[7]. Since the structure of the 1:1 NiMo could not be well characterized in the experiment, we assumed that the NiMo system forms the δ-phase NiMo: $Ni_{24}(Ni_4Mo_{16})Mo_{12}$[8, 9], and its (100) face forms the surface of the NiMo system. The lattice constant of the NiMo(100) surface was known to be 8.852 × 9.108 Å, while the lattice constant of the orthorhombic 2 × 4 cell of graphene, 8.52 × 9.838 Å. To compensate such a lattice mismatch of the graphene layer and the NiMo(100) surface, we used the surface lattice constant of 8.852 × 9.563 Å. Note that this does not change the structures of both the NiMo(100) surface and the graphene layer. With this lattice constant, we put N-doped graphene layers on the NiMo(100) surface. A 20 Å vacuum region was inserted to avoid the artificial interaction between slabs. The Brillouin zone was sampled with the Monkhorst-Pack 4 × 4 × 1 k-grid. The snapshots of the proton transfer are shown in Supplementary Fig. 41.

We also calculated the energy barriers for proton penetration in the presence of

aqueous environment using the CP2K program. The simulation cell lengths in the *x*-, *y*-, and *z*-directions were 25.56, 24.595, and 70 Å, which means that the *z*-length was increased by 20 Å to include water molecules. We added 300 water molecules on both sides of a defect-free graphene and a graphene with an SV-3N defect. After the 1 ns of equilibration using classical force fields[10], we further performed 5 ps of equilibration using DFT molecular dynamics simulation. The temperature was kept to be 320 K using the canonical sampling velocity rescaling method. DZVP/TZV2P basis was used for graphene and water, respectively. The auxiliary plane-wave cutoff was set to 320 Ry. Subsequently, we added H^+ to a water molecule near the interface and optimized the whole structure. Based on the optimized structures, the nudged elastic band method was applied to calculate the penetration barrier. The proton penetration barrier for the pristine graphene does not affected by the explicit water (from 3.16 to 2.97 eV) and the barrier height is consistent with a similar report[11]. On the other hand, the proton penetration barrier for the graphene with an SV-3N defect decreased from 3.30 to 1.93 eV, because the H_3O^+ carries the proton close to the defect. Those snapshots are shown in Supplementary Fig. 38. We note that the proton penetration barriers are still higher than estimated values from experiments, which would be attributed to the atomic defects or bias potential[11].

We further calculated the proton penetration barrier from H_3O^+ in water to bilayer graphene with SV-3N defects for step 1−3, because the similar reduction of barrier was expected. We added 400 water molecules to one side of bilayer graphene with SV-3N defects (Supplementary Fig. 40) and followed the same procedure described above. The energy barrier reduced from 4.63 to 2.00 eV. Finally, the activation energy of the interlayer proton transfer was calculated using the same system. The calculated value was 1.56 eV for water/graphene → graphene and 1.61 eV for graphene → water/graphene. Since the activation barrier without water was 1.53 eV (Supplementary Fig. 40), the effect of water molecules on the interlayer proton transfer is limited. Note, however, that the calculated proton penetration barrier in the presence of water molecules is a sample from various configurations of explicit water molecules.

Finally, we employed the above water/graphene/water structure and after equilibration and calculated the electrostatic potential under the electric field using the

SIESTA code[12]. From the difference in the electrostatic potentials with and without electric field, the x-y averaged (z-direction is perpendicular to the graphene layer) electric field. Supplementary Fig. 23 shows that the voltage drop is dominated at the interface between water layer and graphene surface.

Supplementary discussions

(1) Electrochemical impedance spectroscopy

We used electrochemical impedance spectroscopy to understand the resistance of proton penetration in details. Nyquist plots of the graphene membranes exhibited a semi-circular arc at the frequency range of 10^4 Hz < ω <10^6 Hz. The signal in this region is attributed to proton penetration through the graphene layers (Supplementary Fig. 16b). A clear spike appeared at frequencies ω < 2000 Hz, indicating that positively charged protons were cyclically accumulated and depleted near the Pt electrode surfaces (i.e. ion-blocking) in response to the applied potentials. The Nyquist plot of the Nafion membrane exhibited only a semi-circular arc in the same region of frequencies (Supplementary Fig. 16d).

The impedance spectra were analysed by the equivalent circuit models displayed in the insets of Supplementary Figs. 16b and 16d. In the equivalent circuit of the graphene membrane cell (inset of Supplementary Fig. 16b), R_s represents the resistance of the electrolyte and the two electrodes, R_1 and constant phase element 1 (CPE_1) represent the resistance and capacitance, respectively, associated with proton penetration through the graphene membranes, and the capacitive component corresponding to polarization at the Pt/electrolyte interfaces (CPE_2) represents the capacitance at the interface between the electrolyte and Pt electrodes. In the equivalent circuit of the Nafion membrane cell (inset of Supplementary Fig. 16d), R_s, R_1, and CPE_1 represent similar circuit components, but CPE_2 was absent. This feature indicates that the accumulation/depletion of positive charges (i.e. protons) at the electrode/electrolyte interfaces is rather low in the cell with only the Nafion membrane. It means that counter anions can penetrate through a Nafion in contrast to a graphene where only protons can penetrate but the anion penetration is blocked. The R_1 values of graphene membranes increased with the number of graphene layers (Supplementary Table 4). The R_1 of the Nafion membrane is 20 times less than that of the graphene membranes, which confirms that the resistance to proton penetration originates from the graphene layers.

(2) Intercalation of the proton into graphene interlayers

The intercalation of proton into graphene interlayers can further reduce the energy barrier of proton penetration through multilayer graphene. The non-linear (i.e. logarithmic) relationship between the average proton current and the layer number of graphene (Fig. 3f) demonstrates that the interaction between adjacent graphene layers reduces the energy barrier of penetration. In contrast, an alternating Nafion/graphene laminated structure provided a linear relationship between proton current and graphene layer number, due to individual adsorption and desorption steps of proton penetration through each isolated graphene layer (Supplementary Fig. 18b). The differences in configuration provided a much lower energy barrier for the stacked graphene membrane than for the alternating laminated membrane, which is good agreement with DFT calculations (Supplementary Fig. 40b). Thus, protons adsorbed at the defect sites on the outermost graphene layer can reach the NiMo surface by the intercalation into the multilayer graphene.

(3) Characterizations and electrochemical measurements of the graphene-covered NiNP

The graphene-covered NiNP samples were characterized by scanning electron microscopy (SEM), transmission electron microscopy (TEM), X-ray diffractometry (XRD), Raman spectroscopy, and X-ray photoelectron spectroscopy (XPS). SEM and TEM images indicated that the NiNP were deposited on the graphene surface (Supplementary Figs. 24a and 24b). The layer number of covering NGL were controlled by tuning the CVD deposition time (Supplementary Figs. 24c−24e). The NiNP covered by average 1−2, 3, and 6−7 layers NGL (abbreviated as NiNP/1−2NGL, NiNP/3NGL, and NiNP/6−7NGL) (Supplementary Figs. 24f−24h). XRD patterns of the typical NiNP/NGL samples revealed the metallic Ni (JCPDS No. 65-0380) without carbides and oxides (Supplementary Fig. 25a). XPS Ni 2*p* spectrum indicated the metallic Ni state without any carbides (Supplementary Fig. 25c). Raman spectra were characteristic of high-quality N-doped graphene (I_{2D}/I_G ratio = 0.67−1.31). In addition, the I_D/I_G ratios of NiNP/NGL samples did not largely change (0.73 to 0.83).

The HER performances of NiNP/1−2NGL, NiNP/3NGL, NiNP/6−7NGL, and bare NiNP (i.e., without graphene encapsulation) were examined in Ar-saturated 0.5 M H_2SO_4 in a three-electrode system as compared with commercial 10 wt% Pt/C catalysts.

At an initial cycle, the bare NiNP reached to a current density of 10 mA cm^{-2} normalized by the electrode surface area at an overpotential (η_{10}) of 46 mV (vs. RHE), while η_{10} values of 77, 101, and 138 mV were observed for NiNP/1−2NGL, NiNP/3NGL, and NiNP/6−7NGL (Supplementary Fig. 25d). This result indicated that the HER activity was governed by the layer number of graphene on the NiNP samples, consistent with the experimental results of graphene-covered NiMoNP samples (see next section).

(4) Characterizations and electrochemical measurements of the graphene-covered NiMoNP

The graphene-covered NiMoNP samples were characterised by TEM, Energy-dispersive X-ray spectroscopy (EDS), XRD, XPS, and Raman spectroscopy. TEM image indicated that the NiMoNP were deposited on the graphene surface (Supplementary Fig. 26a). EDS revealed that Ni and Mo atoms homogeneously dispersed on the nanoparticle areas (Supplementary Fig. 26b). The layer number of covering NGL were controlled by tuning the CVD deposition time (Supplementary Figs. 26c−26e). The NiMoNP covered by average 1−2, 3, and 6−7 layers NGL (abbreviated as NiMoNP/1−2NGL, NiMoNP/3NGL, and NiMoNP/6−7NGL) (Supplementary Figs. 26f−26h). XRD pattern revealed that the dominated components were NiMo (JCPDS No. 48-1745) and Ni_4Mo (JCPDS No. 65-5480) without carbides and oxides (Supplementary Fig. 27a). XPS Ni 2*p* and Mo 3*d* spectra confirmed that the NiMoNP samples surface mainly comprise metallic Ni and Mo (Supplementary Figs. 27c and 27d). Raman spectra were characteristic of high-quality N-doped graphene (I_{2D}/I_G ratio = 0.57−1.13). Moreover, the I_D/I_G ratio of NiMoNP samples did not largely change (0.78 to 0.87).

The HER performances of the NiMoNP/1−2NGL, NiMoNP/3NGL, NiMoNP/6−7NGL, and bare NiMo (i.e., without graphene encapsulation) were examined in Ar-saturated 0.5 M H_2SO_4 in a three-electrode system as compared with the commercial 10 wt% Pt/C catalysts. HER polarization curves and Tafel slopes (Supplementary Figs. 28a and 28b), the bare NiMo sample was initially more HER active than NiMoNP/1−2NGL, NiMoNP/3NGL, and NiMoNP/6−7NGL, and showed a comparable HER activity with the commercially available Pt/C catalyst. During the initial stages of uses, the bare NiMo sample delivered a current density of 10 mA cm^{-2} normalized by the electrode surface area at an overpotential (η_{10}) of 27 mV relative to the

reversible hydrogen electrode (RHE), while η_{10} values of 64–110 mV were observed for the graphene-covered NiMoNP samples (Supplementary Table 8), which means that graphene covering reduces catalytic activity. However, after 1000 cyclic voltammetry (CV) cycles, the η_{10} required for the bare NiMo sample was 800% higher than the initial η_{10} value at the 1st CV cycle, while the required η_{10} values for the NiMoNP samples with 1–2NGL, 3NGL, and 6–7NGL were 70.3, 13.7, and 0.9% higher than the initial ones at the 1st CV cycle (Supplementary Fig. 28c), respectively, which demonstrates that graphene covering dramatically suppresses the degradation of NiMoNP's catalytic activity.

The chemical corrosion of each sample after cycling was examined by inductively coupled plasma optical emission spectrometry (ICP–OES). Severe metal dissolution was observed for the NiMoNP/1–2NGL sample (i.e., 48.3 at% for Ni and 16.9 at% for Mo), whereas the NiMoNP/3NGL and NiMoNP/6–7NGL samples showed lower dissolution rates of 4.2/1.6 and 1.5/0.3 at% for Ni and Mo, respectively (Supplementary Fig. 28d). In addition, the NiMoNP samples with thin graphene layers exhibited high catalytic activities, whereas thick graphene layers resulted in the low dissolution rates (i.e., high corrosion resistance). The NiMoNP/3NGL sample exhibited an excellent balance between corrosion resistance and HER activity. Long-term durability was examined by chronoamperometry (CA) at −150 mV vs. RHE for 25 h in 0.5 M H_2SO_4 (Supplementary Fig. 28e). The NiMoNP/1−2NGL sample retained only 51.1% of its initial current density, whereas the current of the NiMoNP/6−7NGL sample maintained >99% of its initial value. The NiMoNP/3NGL sample displayed a current density of ~44 mA cm^{-2} (92.5% of its initial value), which continued for 25 h with a low rate of leaching of catalysts.

(5) Mo_2C formation on the graphene-covered NiMoNP

Mo_2C is known to be as a predominant component at a high carbonization temperature under a carbon gas atmosphere. The contribution of the HER-active Mo_2C species should influence the proton penetration and the HER activity on the graphene-covered NiMoNP samples. Therefore, the annealing temperature dependence of Mo_2C formation during the CVD process were investigated. In the XRD patterns (Supplementary Fig. 33), the Mo_2C readily formed and the NiMo became a minor component at the carbonization temperature of 800−950 °C. However, the metallic NiMo

were preserved at 700 °C. The XPS spectra of Ni 2*p* and Mo 3*d* of the sample prepared at 700 °C showed major signals of Ni^0 (852.9 eV) and Mo^0 (228.1 and 231.2 eV) with small contributions of Ni and Mo oxides and without any Mo_2C peaks (Supplementary Figs. 27c and 27d). Thus, the metallic state of NiMo alloy is predominant for at the carbonization temperature of 700 °C, which was employed in the main text for NiMo/NGL samples.

(6) Influences of defects on graphene lattice toward the HER activities of the graphene-covered NiMoNP

The NiMoNP/6–7NGL sample with a higher defect density of graphene was synthesized at a lower carbonization temperature of 500 °C, which was used to investigate the relationship between the defect density and the HER activity. The higher defect density of graphene ($I_D/I_G = 1.44$) in the 500 °C-prepared NiMoNP sample was confirmed by Raman spectra as compared to the counterpart of the 700 °C-prepared NiMoNP sample ($I_D/I_G = 0.87$) (Supplementary Fig. 34a). The HER activity of the 500 °C-prepared NiMoNP sample showed an improved initial HER activity (Supplementary Fig. 34b) in Ar-saturated 0.5 M H_2SO_4 in a three-electrode system, owing to the high density of structural defects. However, the poor stability of the 500 °C-prepared NiMoNP sample was also observed. Furthermore, a HER-activity comparison between NiMoNP/1–2NGL and NiMoNP/1–2GL (i.e., covered by 1–2 layers non-doped graphene) was also carried out in Ar-saturated 0.5 M H_2SO_4 in a three-electrode system. Compared with the NiMoNP/1–2NGL sample ($I_D/I_G = 0.78$), the NiMoNP/1–2GL sample showed a very small I_D/I_G value of 0.09 (Supplementary Fig. 34c). After 1000 CV cycles testing, the NiMoNP/1–2GL sample exhibited a better stability than the NiMoNP/1–2NGL sample, owing to the lower number of structural defects on graphene covering layers (Supplementary Fig. 34d). Taken together, these results mean that the higher defect density of graphene enhances both proton penetration and dissolution of NiMo through defect-rich regions on graphene, reducing the catalyst lifetime. Thus, the balance of defect density plays an important role in HER performances (activity and catalyst lifetime).

Complete encapsulation on the metal catalyst has been examined by following a similar acid-treatment method in reference[13]. We measured and compared the HER polarization curves of NiMoNP/6-7NGL samples before and after soaking in 0.5 M H_2SO_4 electrolyte for 100 h at 20 °C. Two types of NiMoNP/6-7NGL samples were

prepared for comparison: (1) as-prepared NiMoNP/6-7NGL sample and (2) NiMoNP/6-7NGL sample treated by the oxygen plasma (30 s, under a pressure of 0.1 MPa) that can intentionally destroy encapsulating graphene layers. TEM images showed the complete graphene layers encapsulating NiMoNPs for the as-prepared sample (Supplementary Figs. 35a and 35b). On the other hand, the plasma-treated sample exhibited an irradiated morphology with incomplete encapsulation by graphene (Supplementary Figs. 35g and 35h). Raman spectrum showed the *D* band intensity drastically increased after plasma treatment (I_D/I_G ratio from 0.87 to 1.28) (Supplementary Fig. 35c), which exhibited the largely increased defect density. Then, we measured the HER activity of these two samples with the same experimental conditions used in main text. The as-prepared sample showed the very similar HER activity before and after 100 h soaking in acid (Supplementary Fig. 35d), and no obvious degradation was observed (Supplementary Figs. 35e and 35f), which further confirmed the complete encapsulation by graphene. In a sharp contrast, the plasma-treated sample exhibited an enhanced initial activity, due to the exposure of NiMoNP's surface to acidic electrolytes, while the HER current significantly decreased after 100 h soaking in 0.5 M H_2SO_4 electrolyte because of the incomplete graphene encapsulation.

(7) DFT simulations of the penetration of generated molecular hydrogen through graphene lattices

As another route, to penetrate through an SV-3N lattice (Supplementary Fig. 43), the H_2 firstly decomposed into two H atoms, adsorbed on the N-dopant sites, penetrated through the defects one by one, and recombined after penetration. This two-step sequence of decomposition and recombination significantly reduced the overall energy barrier of H_2 penetration to 1.15 eV.

Moreover, we evaluated the energy barrier and the order from high energy barrier to low energy barrier of hydrogen molecule penetration as follow.

Perfect graphene lattice, 5-7 and 5-8-5 defects >> split and recombination through N-doped graphene lattice > nanopores in Supplementary Table 11 (no split).
Therefore, it is a dominant pathway that hydrogen molecules penetrate without the split and recombination.

(8) Ejection of generated molecular hydrogen through graphene

The ejection of H_2 generated on the Ni surface through graphene layers was investigated by measuring CV with different scan speeds. The NGL-/GL-covered Ni sheets (Supplementary Figs. 44a and 44b), a graphitic rod, and 0.5M H_2SO_4 solution were used as the working electrode, counter electrode, and electrolyte, respectively. Firstly, CV was performed for the NGL-covered Ni sheet under two scan speeds (slow one: 0.01 mV s^{-1} and fast one: 1.0 mV s^{-1}). Through manual operation of suction by a pipette, we confirmed that the generated H_2 bubbles (Supplementary Fig. 44c) were attached on the NGL under both scan speeds of 1.0 and 0.01 mV s^{-1}, and we could not find the bubbles in-between NGL and Ni surface after tossing all removable bubbles (Supplementary Fig. 45a). We also observed that the Raman mapping of *G* band intensity and I_D/I_G intensity ratio before and after reaction did not show any significant change of the NGL (Supplementary Fig. 46a). However, it is in stark contrast with the GL-covered case, where one can clearly see the bubble encapsulation was observed at the low potential range from 0.0 V to −0.2 V (vs. RHE) with a slow scan speed (0.01 mV/s) (Supplementary Fig. 45b). The slow CV scan speed (0.01 mV s^{-1}) resulted in a slow H_2 generation speed, thus the H_2 bubbles encapsulated by defect-less GL could be observed. In another case, the fast CV scan speed (1.0 mV s^{-1}) results in a fast H_2 generation speed, which was much faster than the H_2 ejection speed through GL. Finally, the GL that encapsulates H_2 bubble burst, which confirmed by Raman mapping (Supplementary Fig. 46b). These differences between defect-rich NGL- and defect-less GL-covered cases can be attributed to the balance between the ejection of molecular hydrogen through graphene layers dominated by defects/nanopores and the generation speed of molecular hydrogen dominated by experimental parameters (i.e., scan speed and potential range). Indeed, the defect-rich regions in NGL contribute to the efficient ejection of molecular hydrogen due to the smaller energy barrier (Supplementary Table 11).

Recently, the H_2 permeation through a graphene layer can be explained by the ripples and defects inducing a local curvature as catalytically active sites[14]. Our calculation data suggest that the defects/nanopores induced by N dopants reduced the activation energy barrier of H_2 penetration, achieving a lower value than that for GL (Supplementary Table 11 and Supplementary Fig. 43). Thus, one can conclude that H_2

prefer to be ejected through the nanopores/defects in NGL and then the gathered H_2 molecules form a big bubble.

Supplementary Figures

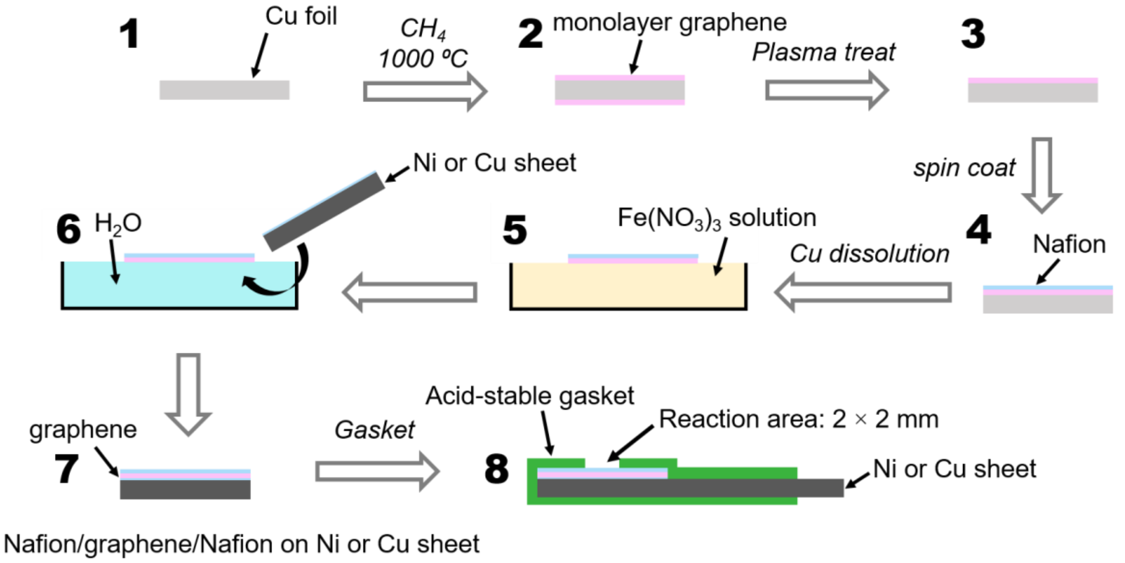

Supplementary Figure 1. Preparation of the transferred CVD graphene-covered Cu and Ni sheets. For preparing the Cu or Ni sheet with multilayer graphene covering, the process was as same as the steps shown in Supplementary Fig. 3.

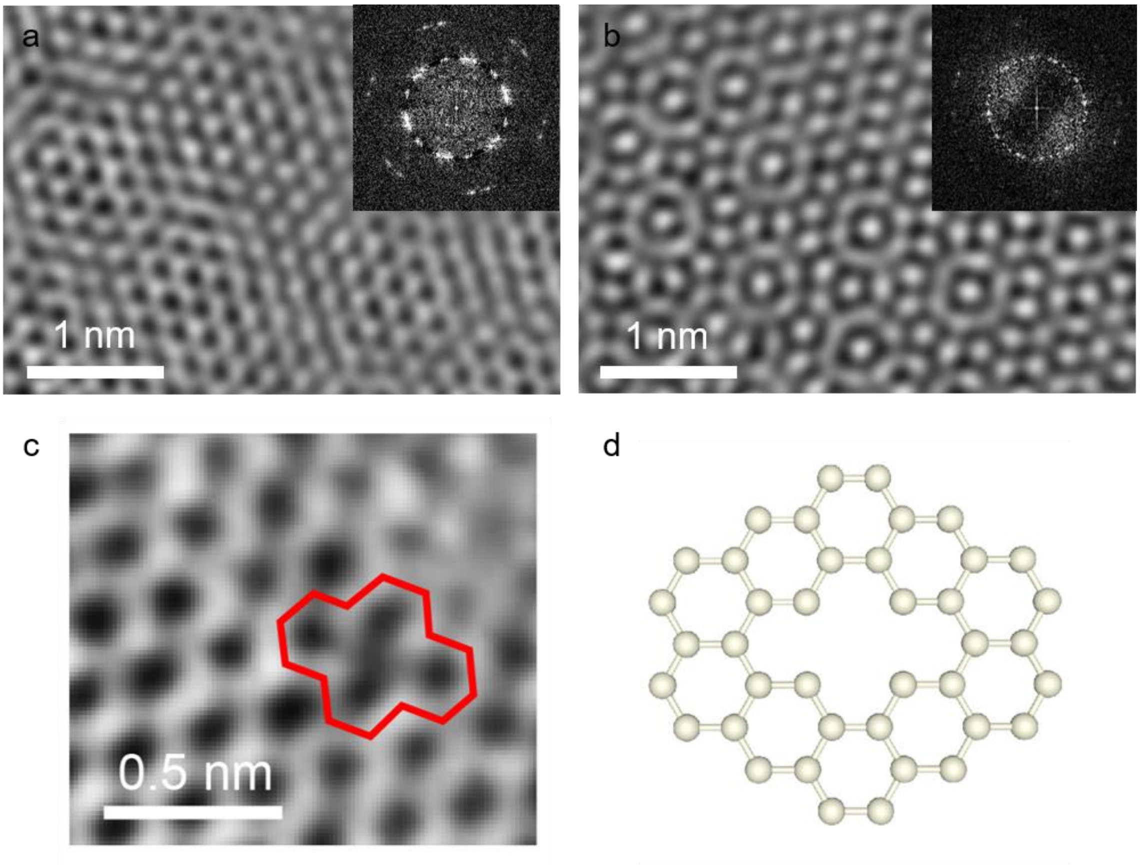

Supplementary Figure 2. Atomic graphene-layer characterization. HRTEM images of (a) bilayer and (b) trilayer non-doped graphene and the corresponding Fast Fourier Transform (FFT) images. (c) Double vacancy observed in N-doped graphene lattices and (d) the corresponding atomic model.

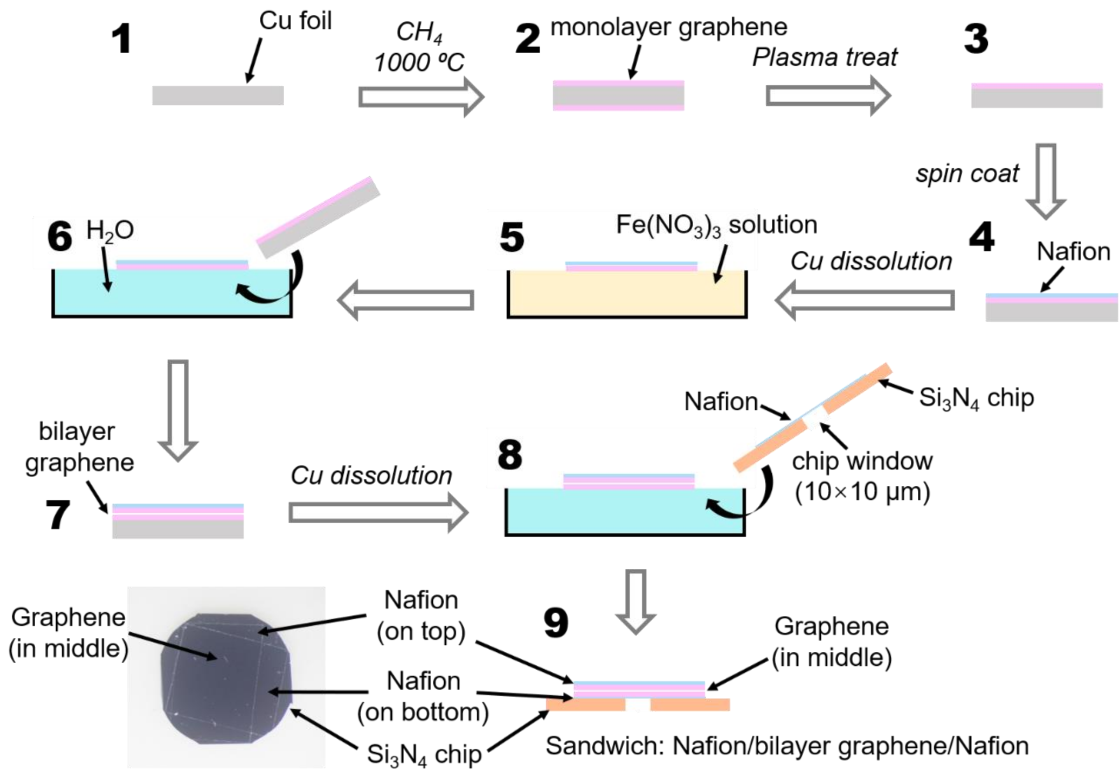

Supplementary Figure 3. Fabrication of a Si_3N_4 chip supported Nafion/bilayer graphene/Nafion membrane. The Nafion/graphene/Nafion membrane on Si_3N_4 chip was used for Raman and proton penetration measurements. For preparing the membrane with multilayer graphene, the process from step 4 to 7 was simply repeated.

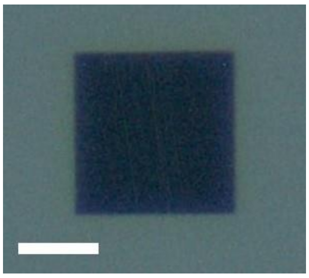

Supplementary Figure 4. An optical photo of the window area in a pristine Si_3N_4 chip. Scale bar: 5 µm.

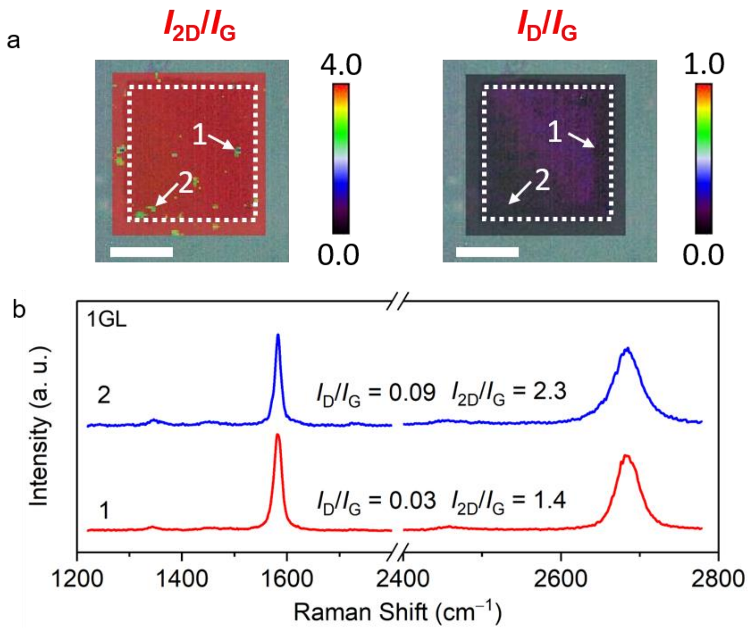

Supplementary Figure 5. Raman characterization of 1GL. (a) Raman map on the window area of the Si_3N_4 chip. The dot square indicated the window area. Scale bar: 5 μm. (b) The corresponding Raman spectra collected at spot 1 (blue area in I_{2D}/I_G image) and spot 2 (yellow area in I_{2D}/I_G image) in the map image. The I_{2D}/I_G values confirmed that 2−3 layers graphene were mainly grown in "yellow" and "blue" areas. The rates of different color areas were summarized in Supplementary Table 1. The monolayer graphene area ("red" area in I_{2D}/I_G image) exceeded 98% in the chip window region.

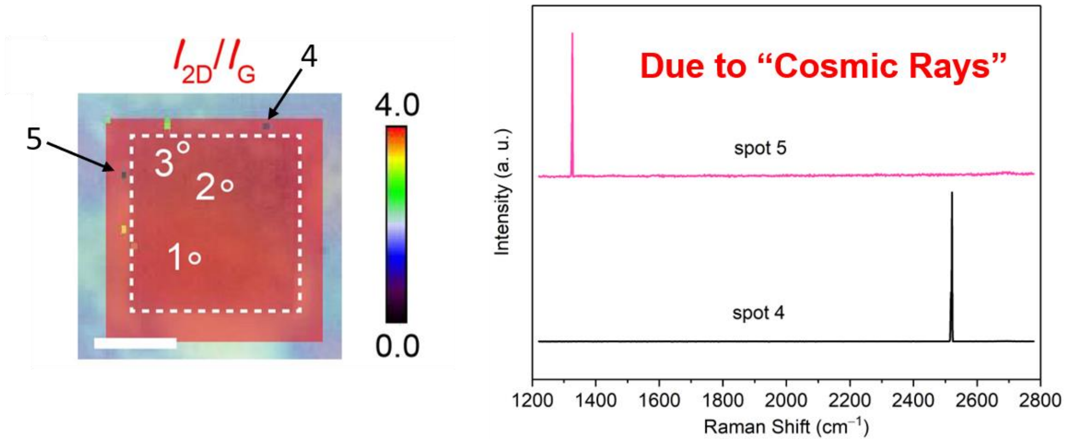

Supplementary Figure 6. Raman characterization of the 1NGL. The corresponding Raman spectra of two black spots were observed in Raman map shown in Fig. 1e. The dot square indicated the window area. Scale bar: 5 μm. These sharp spikes in the spectra were not related to the samples and were generated by cosmic rays hitting the detector, producing spurious features. This event is very common and unavoidable during Raman measurements.

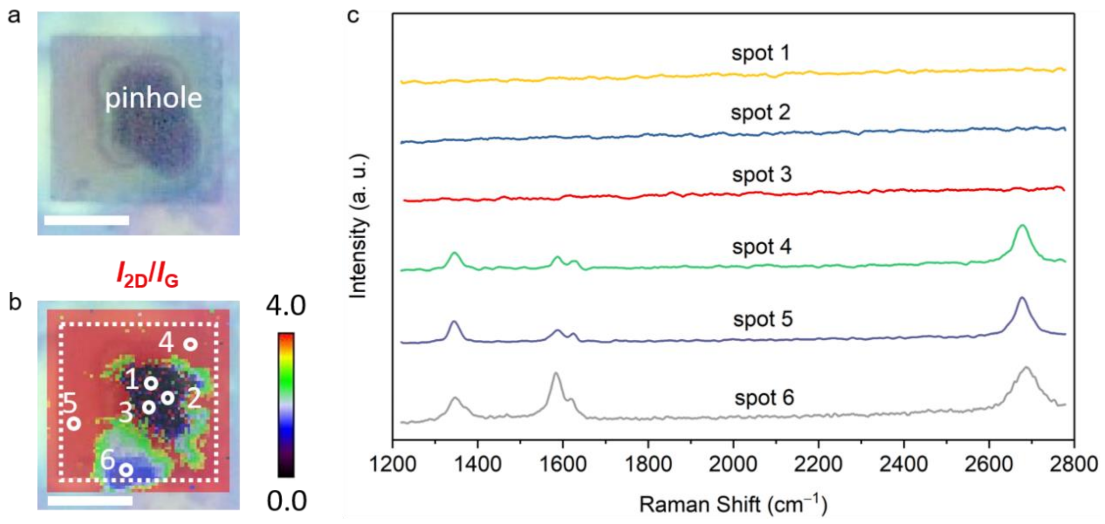

Supplementary Figure 7. Raman characterization of the 1NGL with a pinhole. (a) Optical photo of the 1NGL with a pinhole in the center. The pinhole was made by using high energy laser. (b) The corresponding Raman mapping image. The dot square indicated the window area. Scale bar: (a, b) 5 µm. (c) Raman spectra from six spots pointed in the Raman mapping image. Raman spectra on the pinhole area (1−3 spots) showed no graphene characteristic, while specific Raman spectra of N-doped graphene were clearly observed on the other area (4−6 spots). Importantly, the characteristics of pinholes or breaks were not generated during both the fabrication processes and Raman testing.

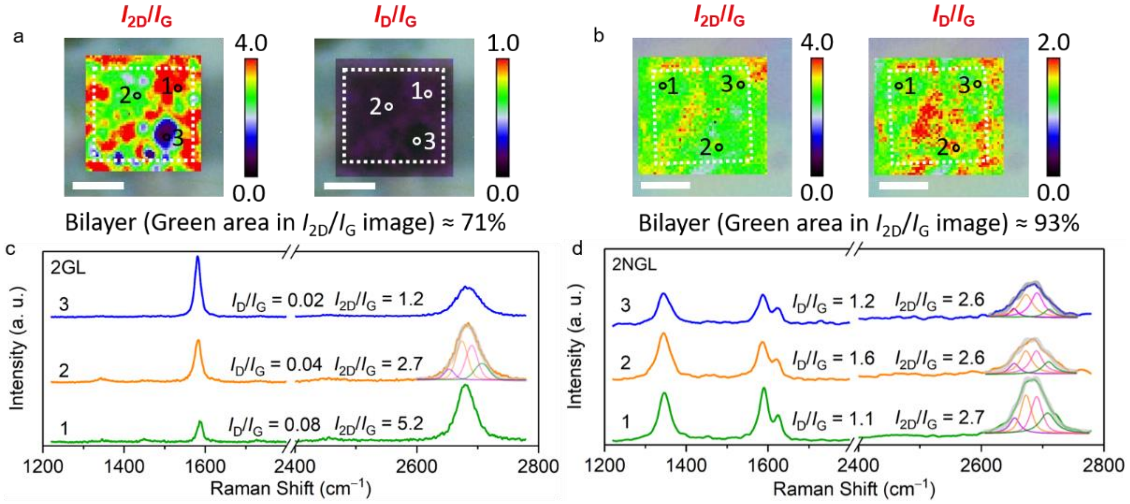

Supplementary Figure 8. Raman characterization of the 2GL and 2NGL. Raman maps of (a) 2GL and (b) 2NGL on the window area in Si_3N_4 chip. The dot square indicated the window area. Scale bar: (a, b) 5 μm. (c, d) The corresponding Raman spectra of the positions pointed in the maps. Lorentzian fitting analyses of the *2D* bands indicated four deconvoluted spectrum for bilayer graphene characteristics[15]. The "red" (spot 1 in I_{2D}/I_G image), "green" (spot 2 in I_{2D}/I_G image), and "blue" (spot 3 in I_{2D}/I_G image) area represented mono-, bi-, and tri-layer graphene, respectively. The calculated rates of bilayer area (green area in I_{2D}/I_G image) were 71% and 93% for the 2GL and 2NGL on the chip window region, respectively.

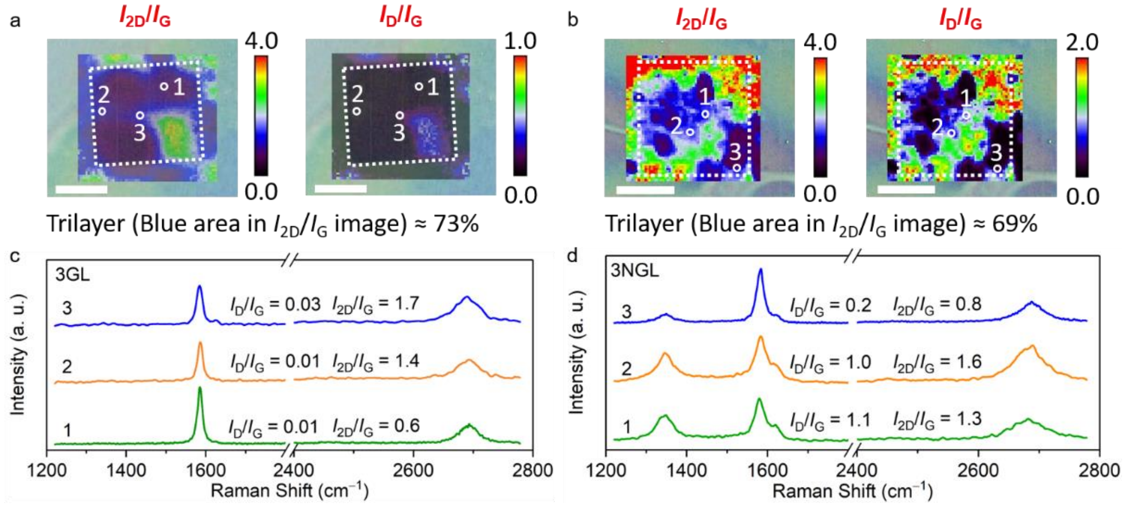

Supplementary Figure 9. Raman characterization of the 3GL and 3NGL. Raman maps of (a) 3GL and (b) 3NGL on the window area in Si_3N_4 chip. The dot square indicated the chip window area. Scale bar: (a, b) 5 μm. (c, d) The corresponding Raman spectra of the positions pointed in the maps. The calculated rates of trilayer area (blue area in I_{2D}/I_G image) were 73% and 69% for the 3GL and 3NGL on the chip window region, respectively.

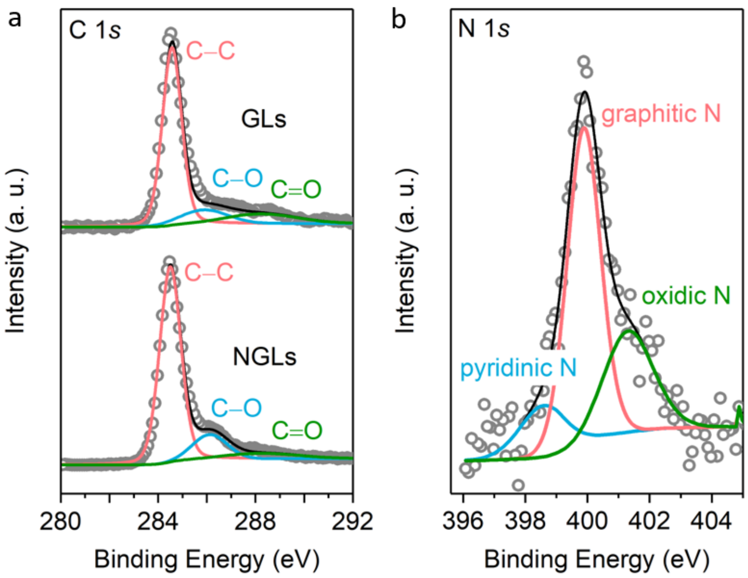

Supplementary Figure 10. High-resolution XPS C and N spectra of graphene. (a) C 1*s* spectrum of the GLs and NGLs. (b) N 1*s* spectrum of the NGLs.

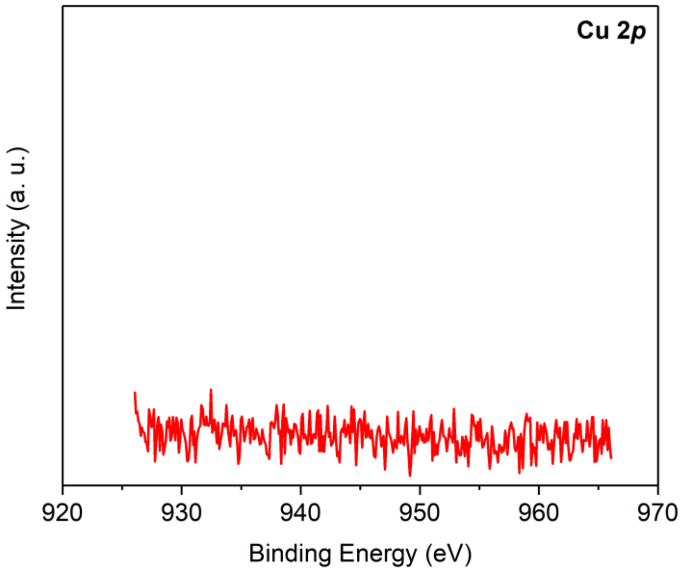

Supplementary Figure 11. Typical high-resolution XPS Cu spectrum of graphene. The residual Cu atomic concentration on the GLs and NGLs was less than 0.01 at%.

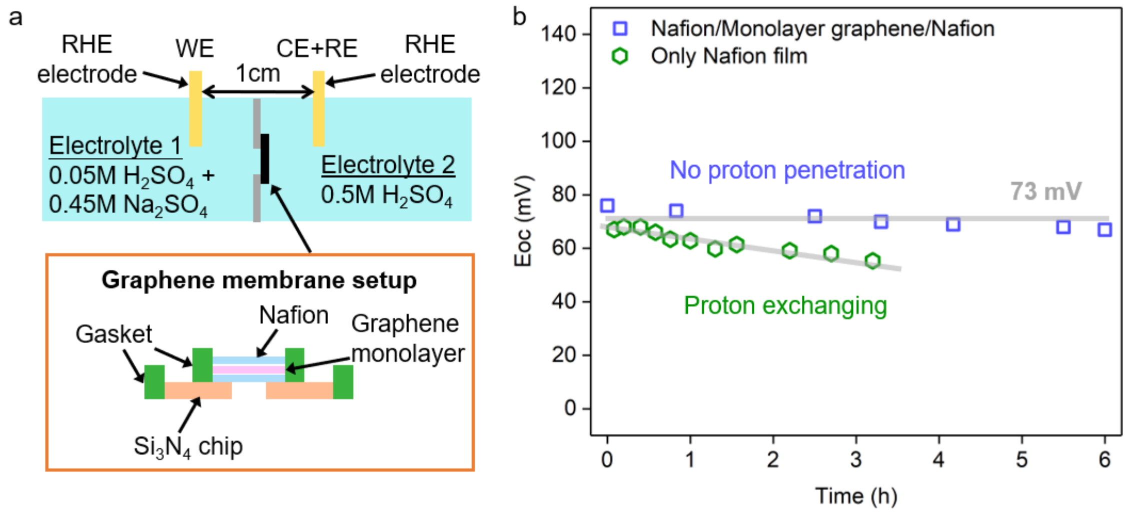

Supplementary Figure 12. Operational check for the H-type cell. (a) Configuration of experimental setting. The 1GL was used to separate two electrolytes with different pH values. Two reversible hydrogen electrodes (RHE) were used as cathode and anode in this two-electrode system. The measured pH values of electrolyte in cathode side and anode side were 1.74 and 0.50, respectively. (b) Measurement of open circuit potential (E_{oc}). In the monolayer graphene membrane case, the E_{oc} value between the separated chambers ranged from 76 to 67 mV (average value: 71 mV) in 6 h, which was very close to the value of 73 mV calculated by the Nernst equation (Calculation details were in Supplementary methods). In only Nafion membrane (sheet thickness: 170 μm) case, the E_{oc} values showed a declining trend, indicating that the protons penetrate through the Nafion sheet under the proton concentration difference.

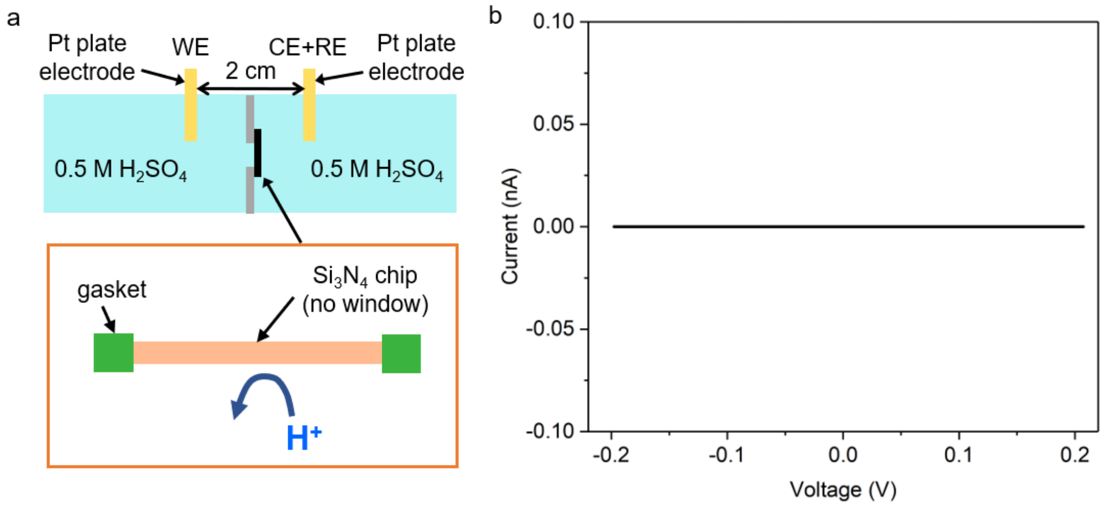

Supplementary Figure 13. *I*−*V* characteristics of a Si_3N_4 chip without the central window. (a) Configuration of the experimental setting without any separating membrane and (b) the *I*−*V* characteristics. This result confirmed that there are no apparent current and leakage of electrolytes.

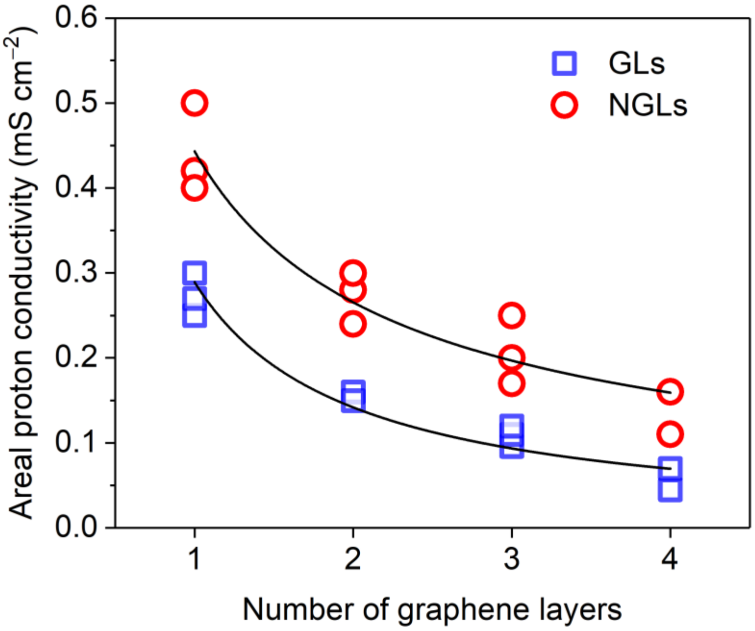

Supplementary Figure 14. Layer number dependences of the proton conductivity for the GLs and NGLs. Areal conductivity was calculated through equation $\sigma = S/A$, where S was the conductivity and A was the area of the window in Si_3N_4 chip. The conductivity $S = I/V$, where the proton current I and bias voltage V were collected from the $I-V$ characteristics (Fig. 3d). The proton conductivity exhibited a declining trend with the increasing of the number of graphene layers, which confirmed that the layer number of graphene significantly affects the resistance for proton penetration. The S value was 43 mS cm^{-2} when only Nafion set as the separating membrane.

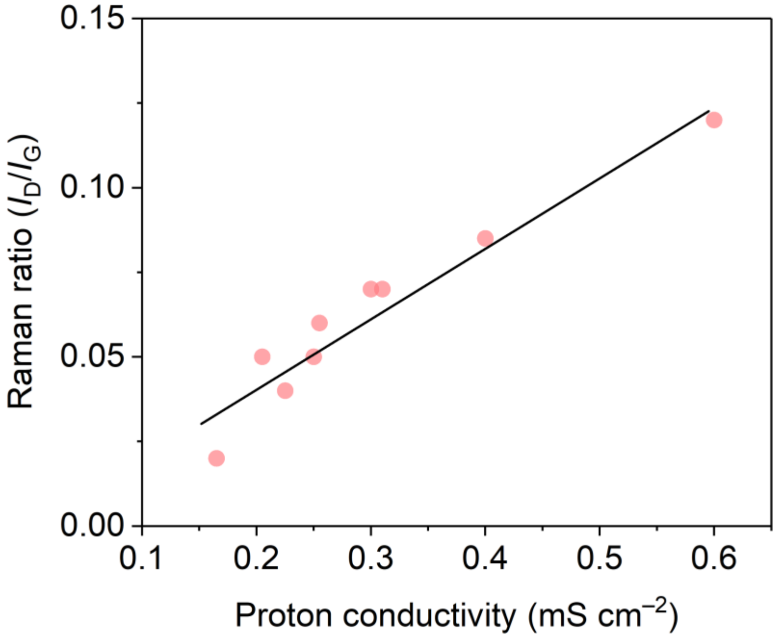

Supplementary Figure 15. Graphene defect dependences of the proton conductivity for monolayer GL. The Raman intensity ratio of *D* band to *G* band (I_D/I_G) represented the defect density of graphene. The proton conductivity through monolayer GL, ranged from 0.16 to 0.6 mS cm^{-2}, showed an increased trend with increasing the defect density (i.e., large I_D/I_G value).

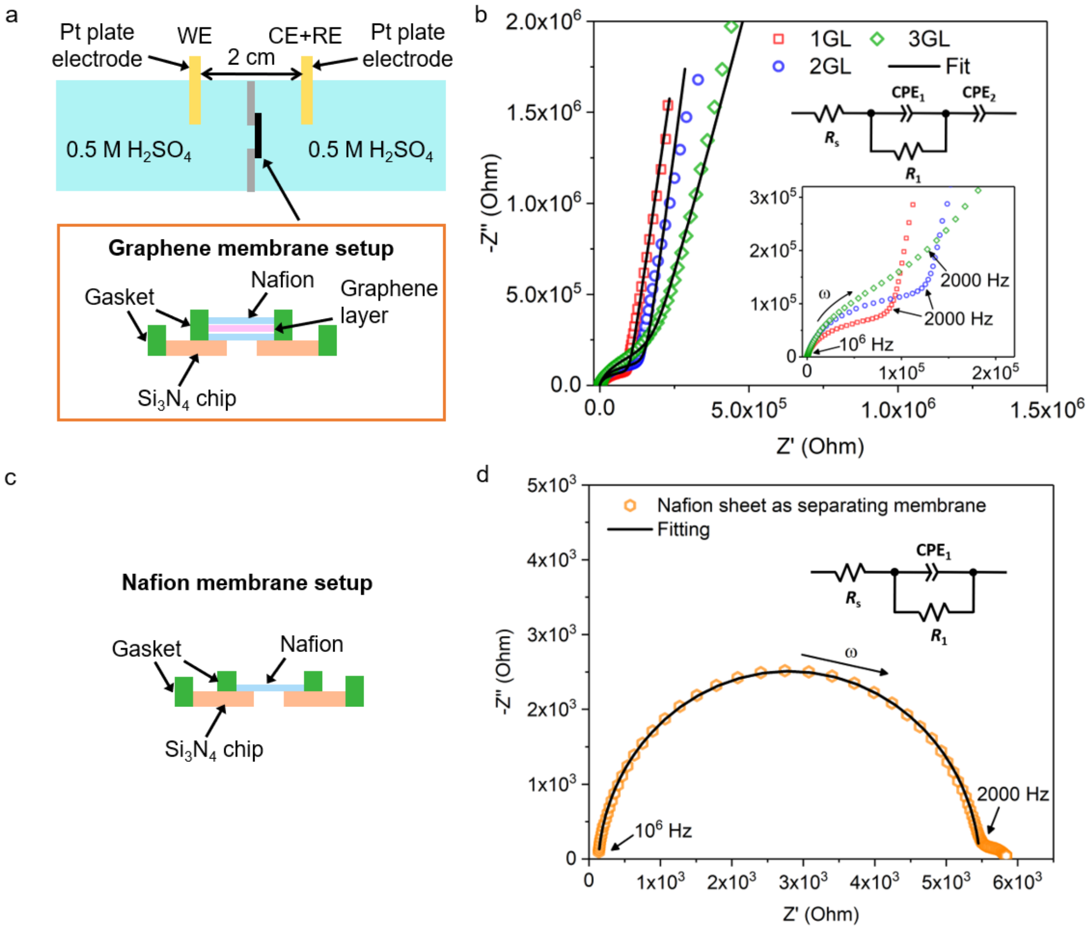

Supplementary Figure 16. Electrochemical impedance measurements. Configurations of the experimental H-type devices with (a) the graphene and (c) the Nafion as separators for electrochemical impedance measurements in a two-electrode system. Nyquist plots and equivalent circuits for (b) 1GL, 2GL, and 3GL and (d) Nafion at a cell voltage of 1.6 V. Insets of (b) and (d): the corresponding equivalent circuit models.

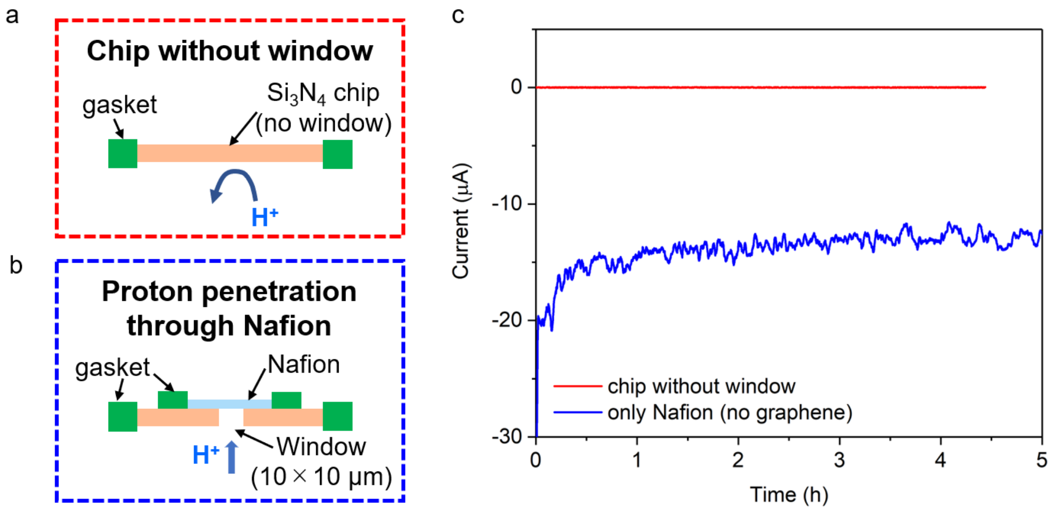

Supplementary Figure 17. Chronoamperometry (CA) measurements and setups. (a) A configuration of the Si_3N_4 chip without the central window and (b) the Si_3N_4 chip with only Nafion on the window area. (c) CA current at a working electrode potential of –20 mV vs. RHE in a three-electrode system. The configuration (a) yielded no apparent current, blocking drift/diffusion of proton. The configuration (b) yielded a large current (15 μA), which was several orders of magnitudes larger than those currents through graphene layers (Fig. 3e), revealing that the proton penetration through Nafion was much easier than that through graphene layers.

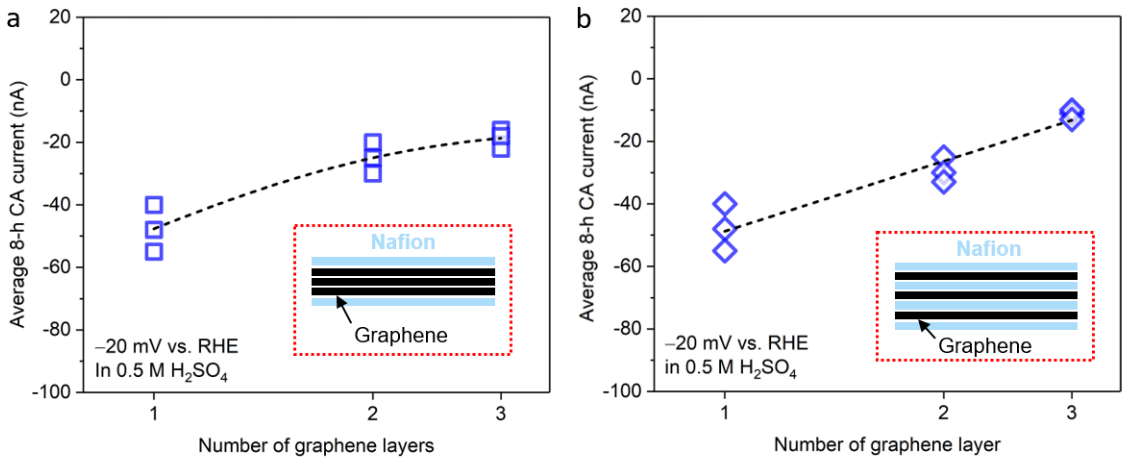

Supplementary Figure 18. Average proton currents of the 8h CA test from two kinds of stacking configurations in separating membranes. (a) The Nafion were only placed on the two outermost sides of graphene. (b) The Nafion were in each inside of adjacent graphene.

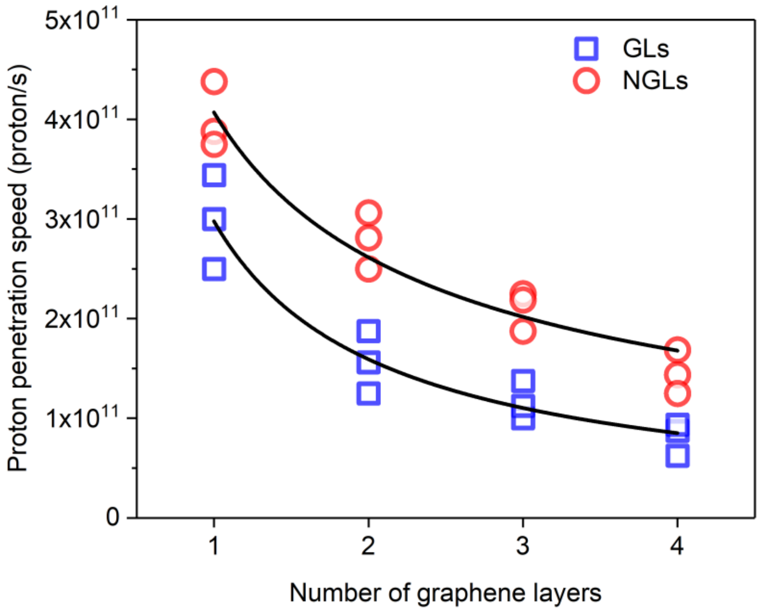

Supplementary Figure 19. Calculated speed of the proton penetration through graphene layers. The speed was calculated by the equation:

$$n = \frac{Q}{e} = \frac{I \times t}{e}$$

where n was the proton penetration speed, Q was the quantity of electric charge, I was the average current collected during 8 h CA testing, t was the penetration time, and e was the elementary charge (1.6×10^{-19} C).

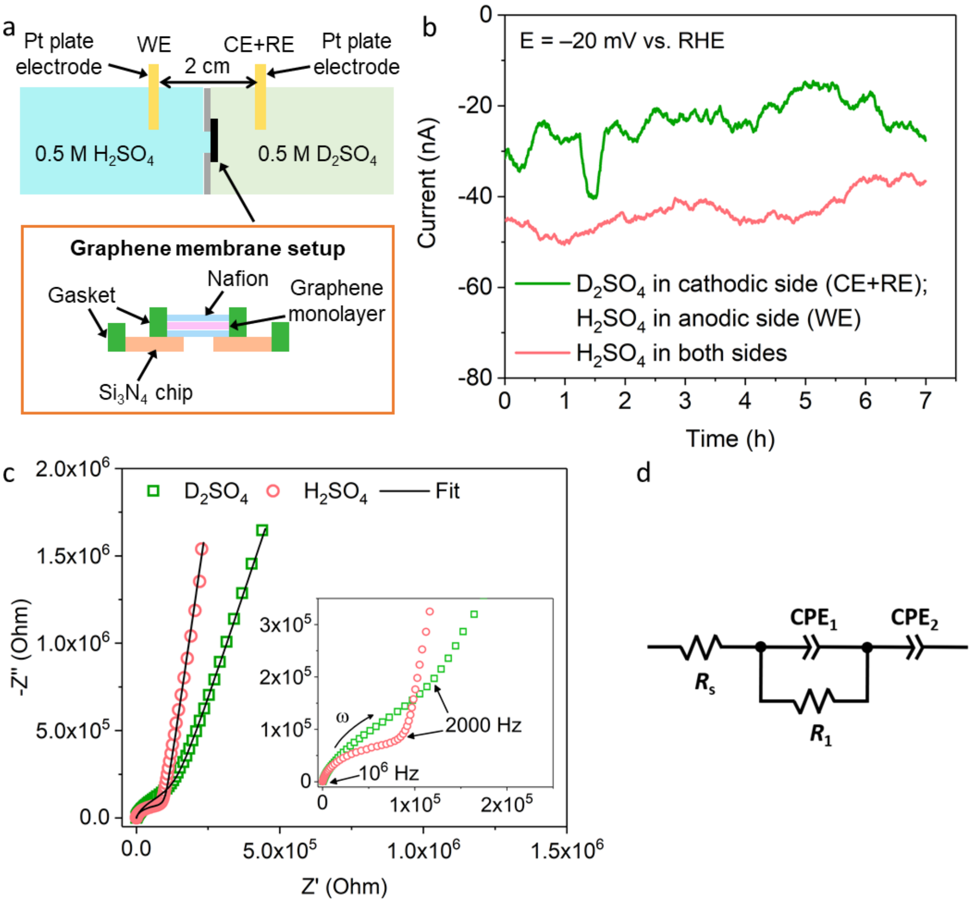

Supplementary Figure 20. Isotope experiments. (a) Configurations of the H-type devices with the 1GL as a separator. (b) CA measurements at a cathode potential of −20 mV vs. RHE in a three-electrode system in 0.5 M H_2SO_4 or 0.5 M D_2SO_4 electrolyte. (c) Nyquist plots in 0.5 M H_2SO_4 or 0.5 M D_2SO_2 electrolyte in a two-electrode system with (d) an equivalent circuit. Proton current was 1.4−1.7 times higher than that of the deuteron current. Electrochemical impedance measurements were performed at a cell voltage of 1.6 V with the frequency range from 10^6 to 100 Hz. In the expected circuit, R_1 values were 95.3 × 10^3 Ω (H_2SO_4 electrolyte) and 115.8 × 10^3 Ω (D_2SO_4 electrolyte).

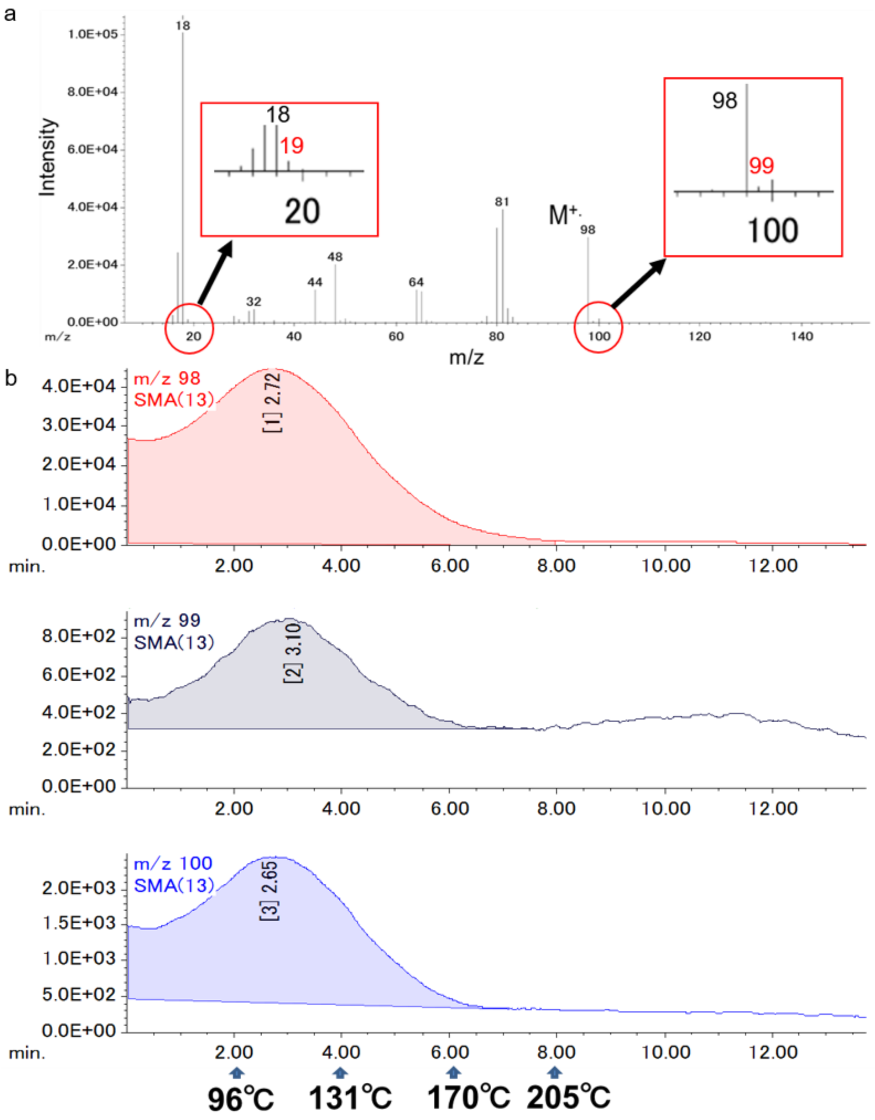

Supplementary Figure 21. Deuterium ion penetration through 1GL. (a) GC-MS chart for the detection of deuterium ions. The detections of $HD^{32}SO_4$ (m/z 99) and HDO (m/z 19) were highlighted in red. (b) Extracted ion chromatograms for the detection for deuterium ions (m/z 98, 99, and 100). Allows on the time axis represented temperatures inside the chamber.

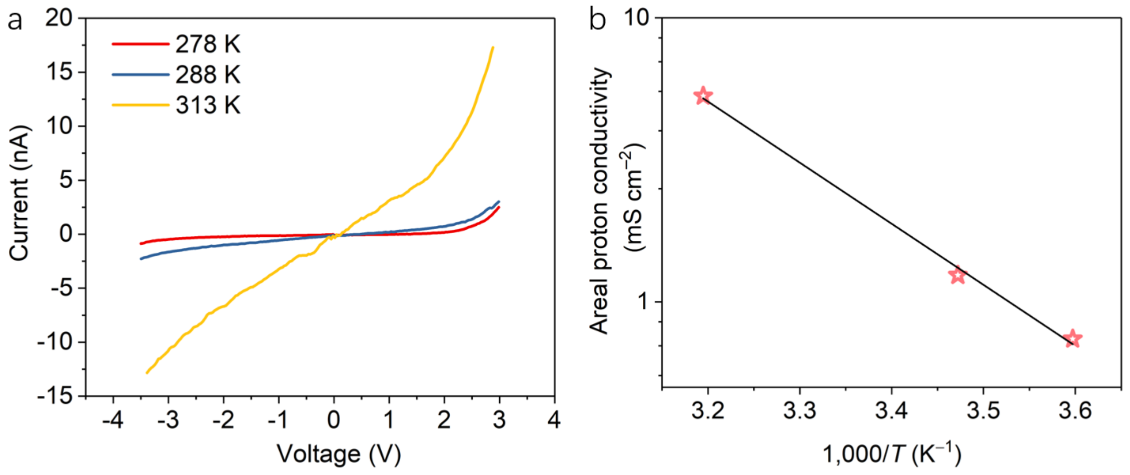

Supplementary Figure 22. Experimental activation energy of proton penetration through 6NGL with under the high cell voltage. (a) *I*–*V* characteristics of proton penetration through 6NGL at various temperature. (b) Temperature dependence of proton conductivity for 6NGL under high cell voltage (−3.5 to −3.0 V). Note that the 1GL, 1NGL, and 3NGL were easily broken at the high cell voltage beyond ± 2.0 V.

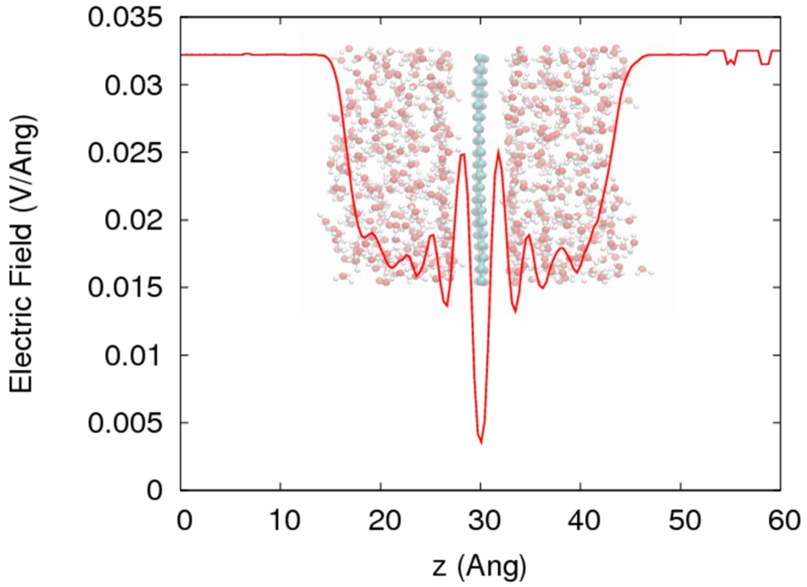

Supplementary Figure 23. Electric field behaviours in water/graphene/water model. We simulated where the effective electric field was applied in a vacuum/water/pure graphene/water/vacuum model under a 0.5×10^9 V/m (= 0.5 V/1 nm) electric field to the model. In the simulation, the maximum voltage drops significantly occurred at the interface between water layer and graphene surface and the voltage drop was equivalent to 0.22 V.

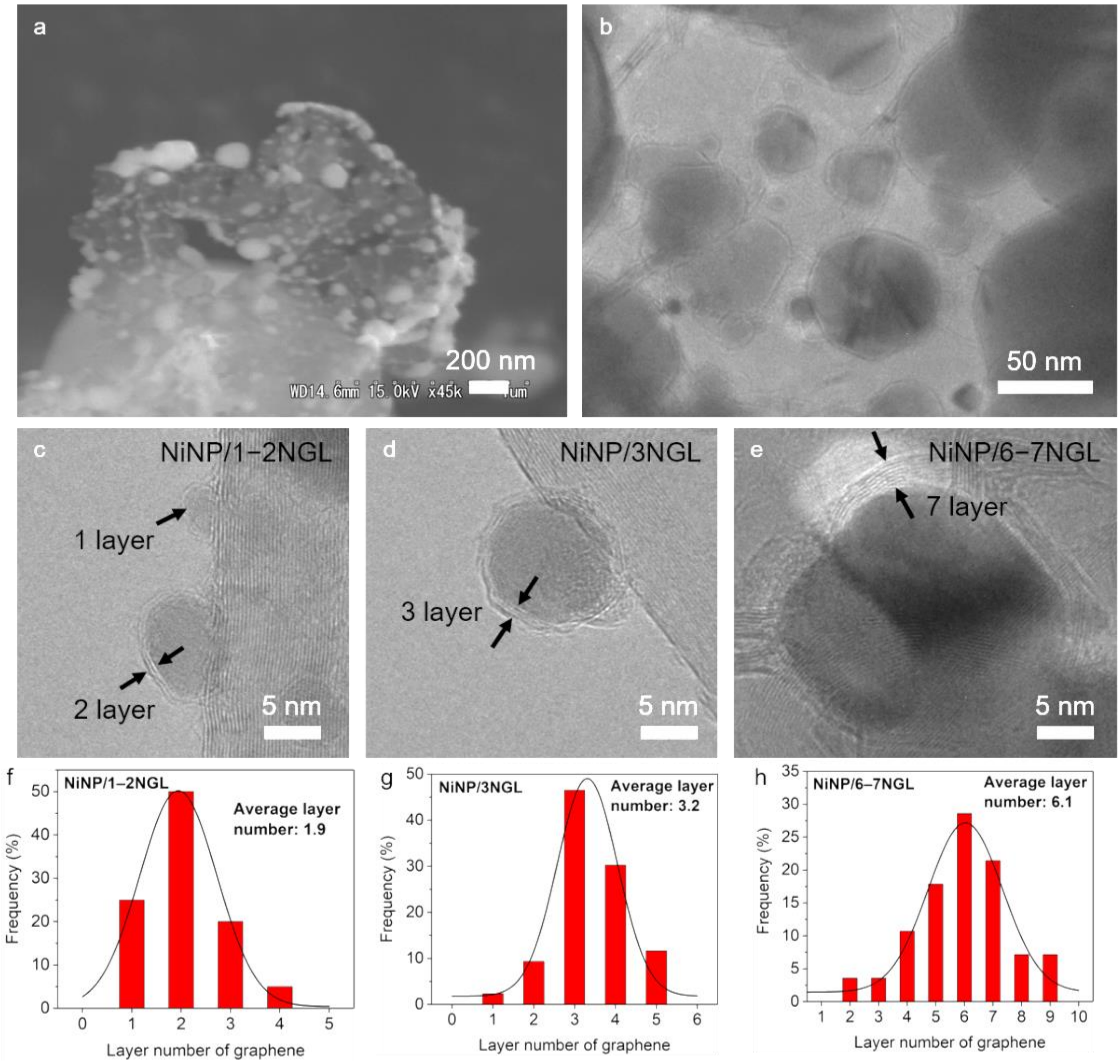

Supplementary Figure 24. Structural characterizations of N-doped graphene-covered Ni nanoparticles (NiNP/NGL). (a) Low-magnification SEM image and (b) TEM image. (c-e) HRTEM images of NiNP covered by controllable layer numbers of graphene. The frequency distribution histograms of the layer number of graphene in (f) NiNP/1−2NGL, (g) NiNP/3NGL, and (h) NiNP/6−7NGL samples, respectively.

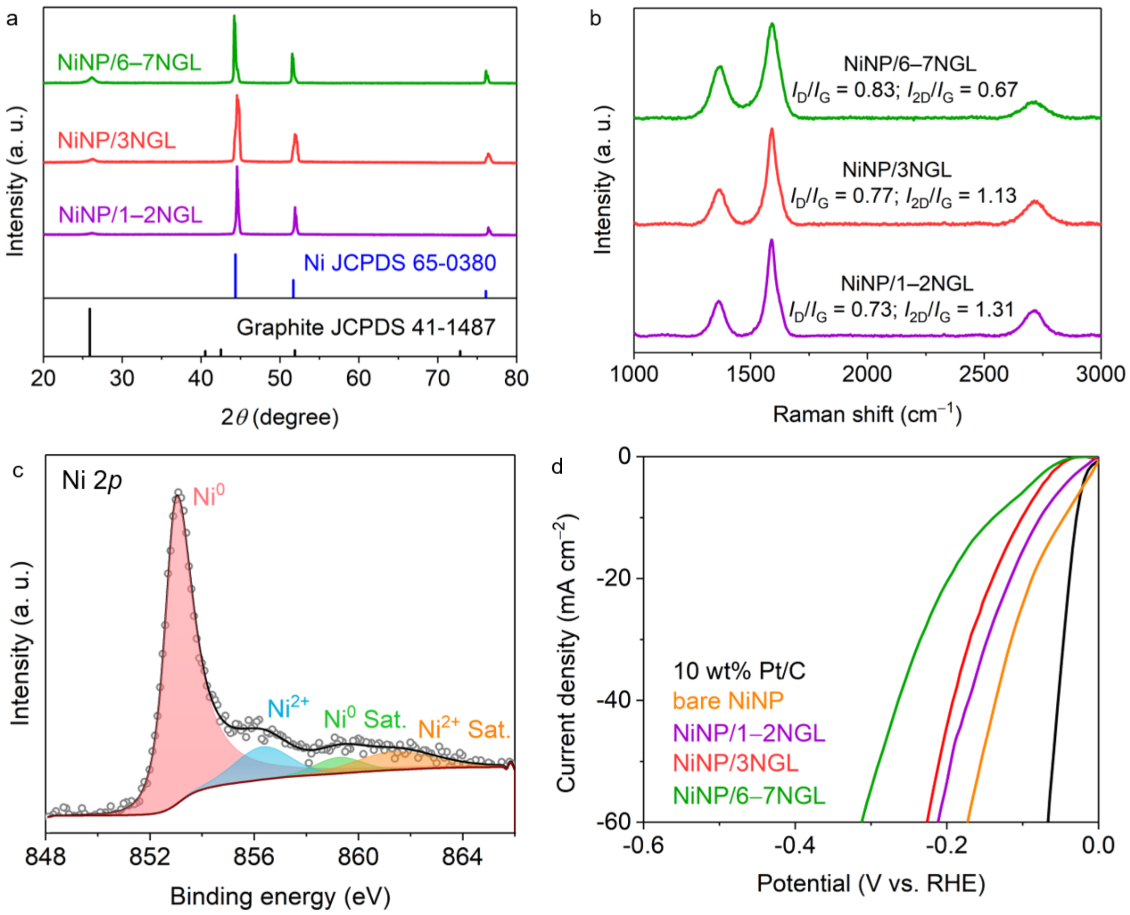

Supplementary Figure 25. Characterizations and HER activities of NiNP/NGL samples. (a) XRD patterns, (b) Raman spectra, (c) XPS Ni 2*p* spectrum, and (d) HER polarization curves of various NiNP/NGL samples.

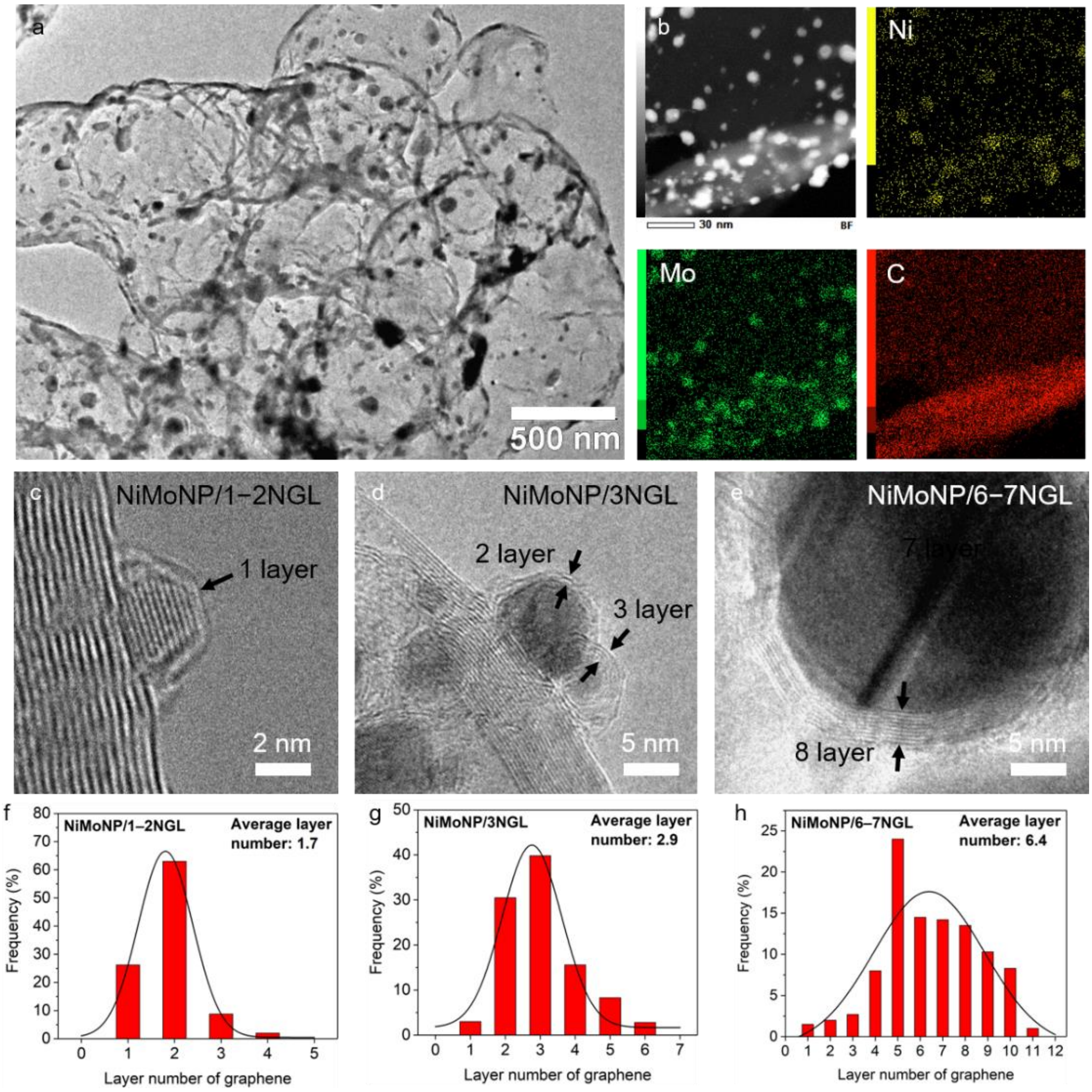

Supplementary Figure 26. Structural characterizations of N-doped graphene-covered NiMo nanoparticles (NiMoNP/NGL). (a) Low-magnification TEM image and (b) EDS map. Reproduced with permission from ref. 7. Copyright 2018 American Chemical Society. (c-e) HRTEM images of NiMoNP covered by controllable layer numbers of graphene. The frequency distribution histograms of the layer number of graphene in (f) NiMoNP/1−2NGL, (g) NiMoNP/3NGL, and (h) NiMoNP/6−7NGL samples, respectively.

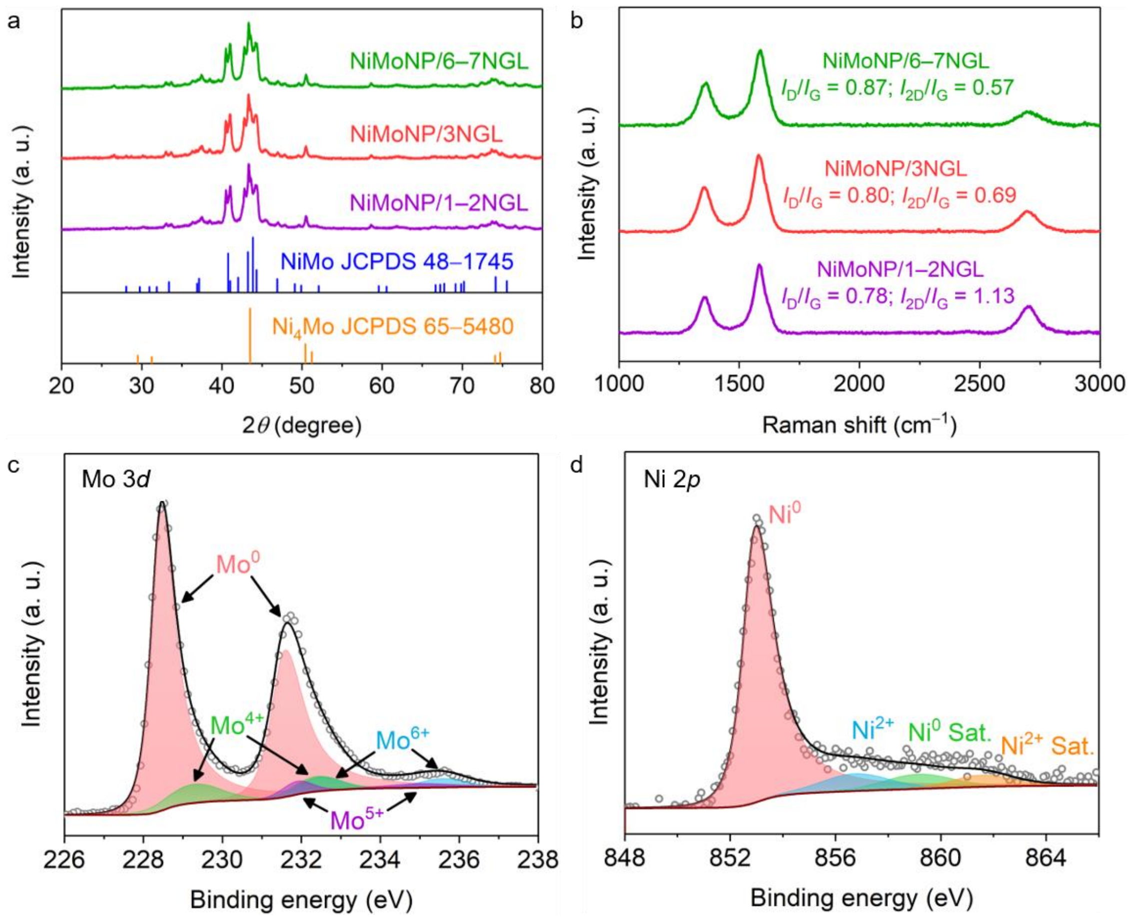

Supplementary Figure 27. Characterizations of NiMoNP/NGL samples. (a) XRD patterns, (b) Raman spectra, (c) XPS Mo 3*d* spectrum, and (d) Ni 2*p* spectrum of various NiMoNP/NGL samples.

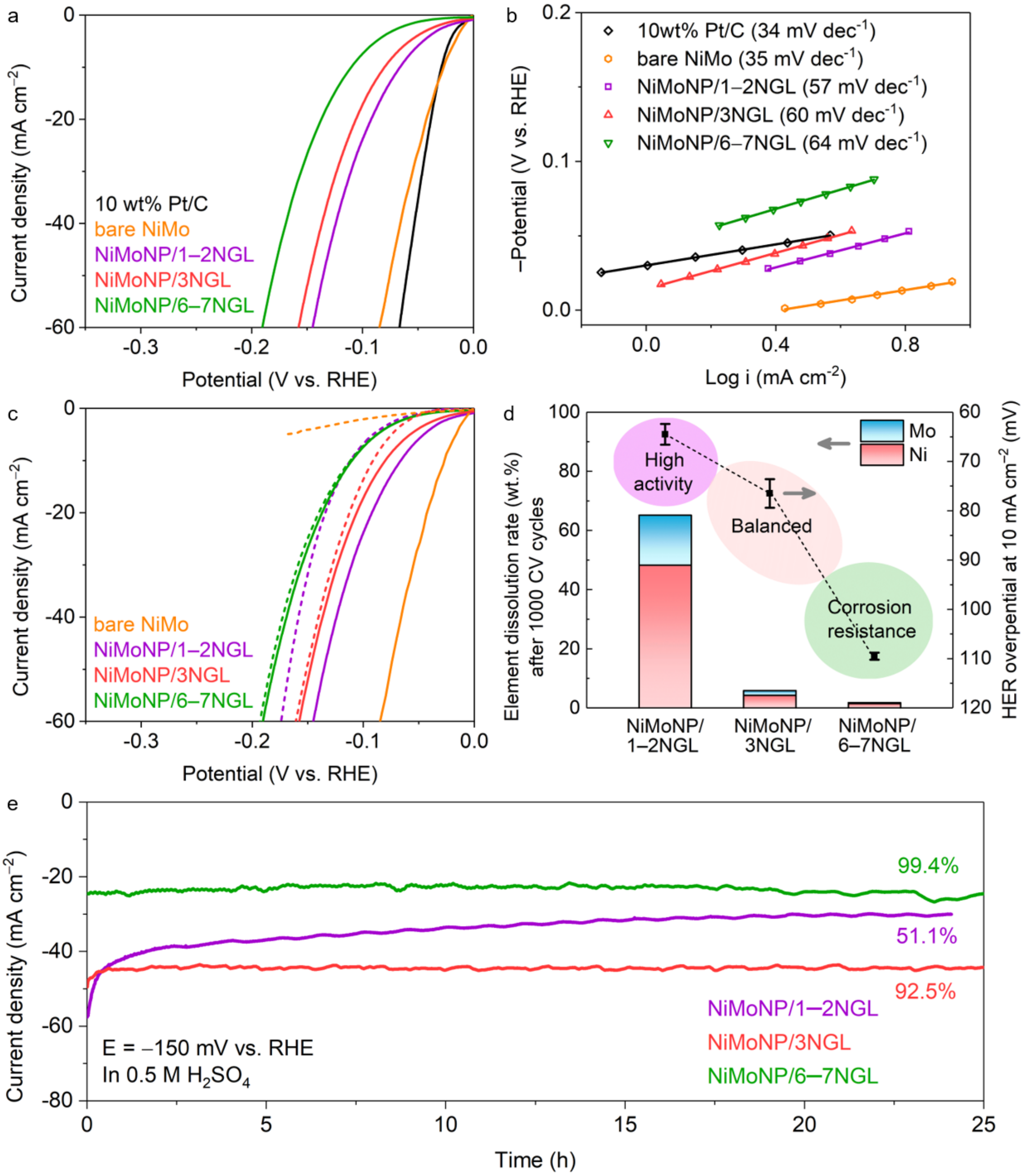

Supplementary Figure 28. HER performances of NiMoNP/NGL samples. (a) HER polarization curves and (b) Tafel plots of NiMoNP/NGL samples, bare NiMo alloy, and commercial 10 wt% Pt/C in 0.5 M H_2SO_4 electrolyte. (c) HER polarization curves before (solid curves) and after (dashed curves) 1000 CV cycling. (d) Ni and Mo dissolution rates in NiMoNP/NGL samples after 1000 CV cycling. The dissolution rates were average values of three individual experiments. Error bars show the fluctuations in the measured signals. (e) Chronoamperometry of NiMoNP/NGL samples. The loading amount of all catalysts: 5 mg.

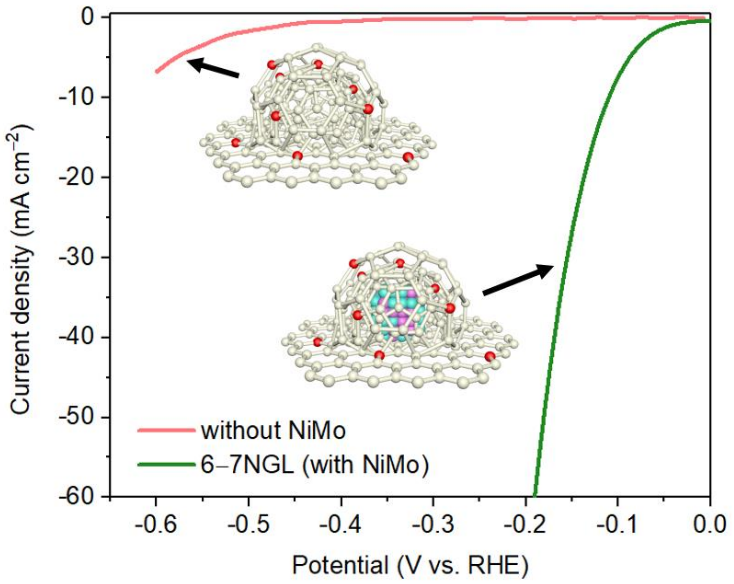

Supplementary Figure 29. HER polarization curves of the NiMoNP/6−7NGL samples with and without NiMoNP substrates. Colour code: carbon (white), nitrogen (red), nickel (cyan), and molybdenum (purple). The NiMoNP/6−7NGL sample without NiMoNP substrates showed a very low HER activity compared to the NiMoNP/6−7NGL sample with NiMo substrates. The 6−7NGL covered sample was a good target to verify the proton penetration effect on HER activity, because of two major reasons. One was the charge transfer effect from the underlying metal substrates to graphene covering layers was limited to three layers.[13] The 6−7NGL can totally exclude the charge transfer effect. The other reason was that the phenomenon of proton penetration through graphene layers was not observed beyond 10NGL (Fig. 3e). Thus, we concluded that the 6−7NGL was the best sample to investigate proton penetration effects on HER in absence of the charge transfer effect.

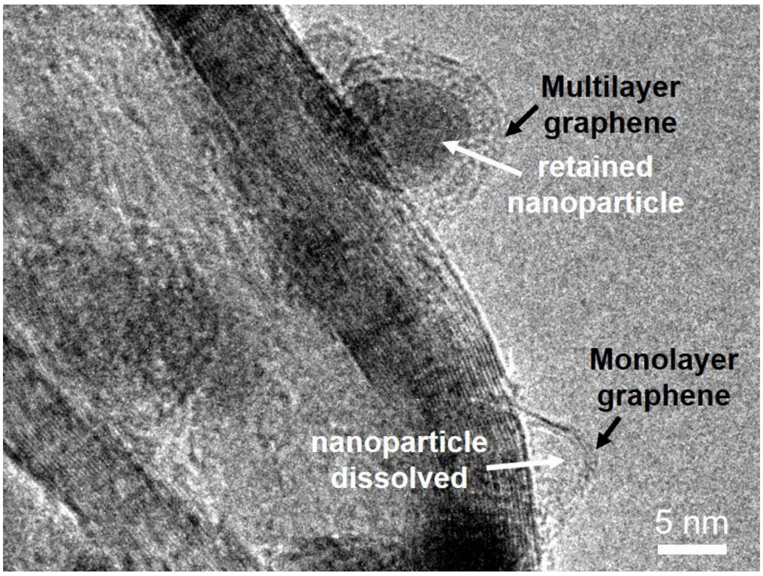

Supplementary Figure 30. TEM image of the NiMoNP/1−2NGL sample after 1000 CV testing cycles in 0.5 M H_2SO_4 electrolyte. The dissolution of the NiMoNP encapsulated by a monolayer graphene after 1000 CV testing cycles was clearly observed. However, another nanoparticle encapsulated by multilayer graphene retained after 1000 CV testing cycles. Reproduced with permission from ref. 7. Copyright 2018 American Chemical Society.

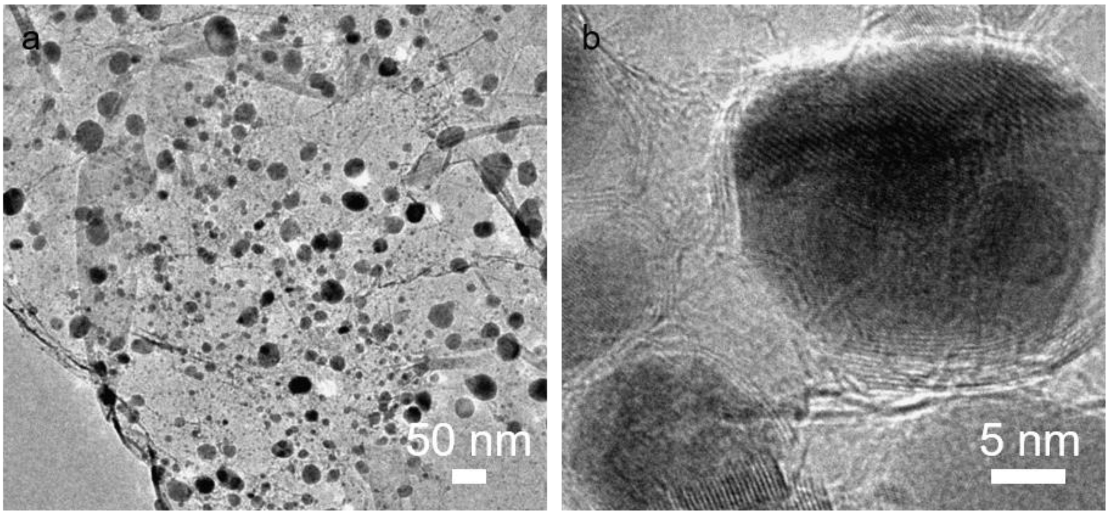

Supplementary Figure 31. TEM image of the NiMoNP/6−7NGL sample after 1000 CV testing cycles in 0.5 M H_2SO_4 electrolyte. (a) Overview and (b) Zoom-in images. No dissolution and morphology changes observed.

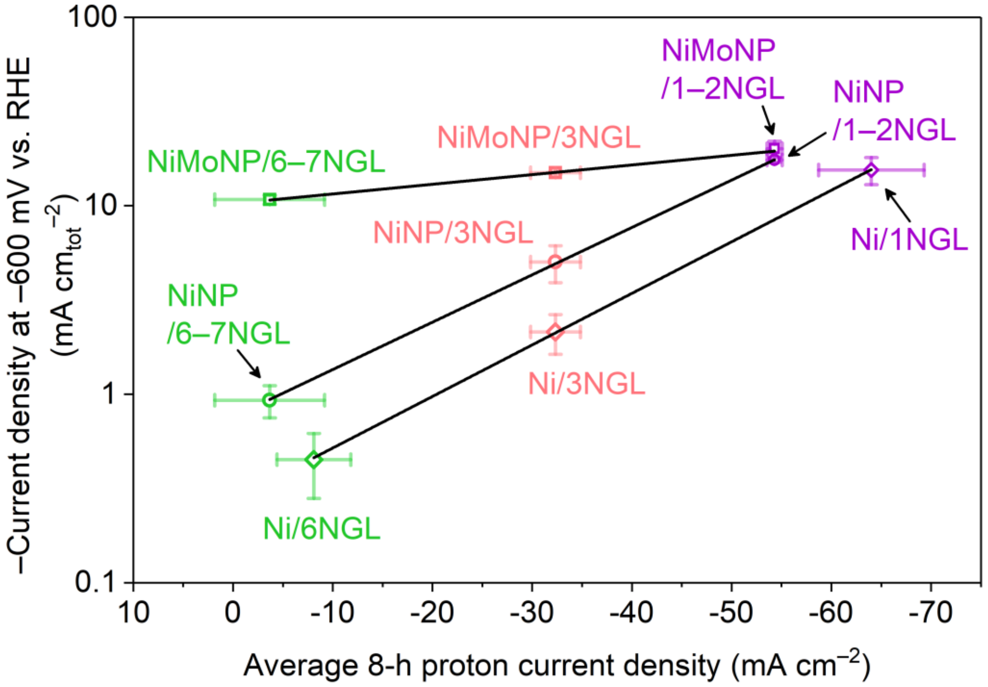

Supplementary Figure 32. Correlation between catalytic activity and proton penetration through the graphene layers in graphene-covered Ni sheet, NiMoNP and NiNP samples. The absolute current density normalized by the total surface area of the catalysts (i.e., the BET surface area for the nanoparticle samples) in the log scale plotted as a function of the average 8 h proton current density (normalized by the SiN chip window area). The black lines were the fits to the correlations. Error bars show the fluctuations in the measured signals.

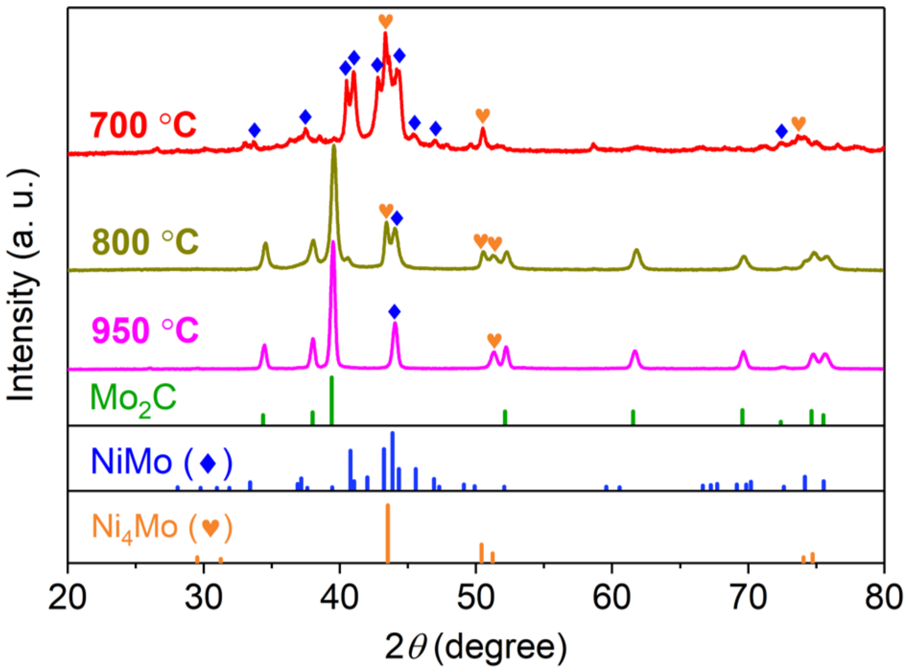

Supplementary Figure 33. Compositions of NiMoNP/NGL samples prepared at various carbonization temperatures ranged from 700 to 950 °C. Mo_2C was readily formed at a temperature higher than 700 °C during the graphene growth process in a CVD system.

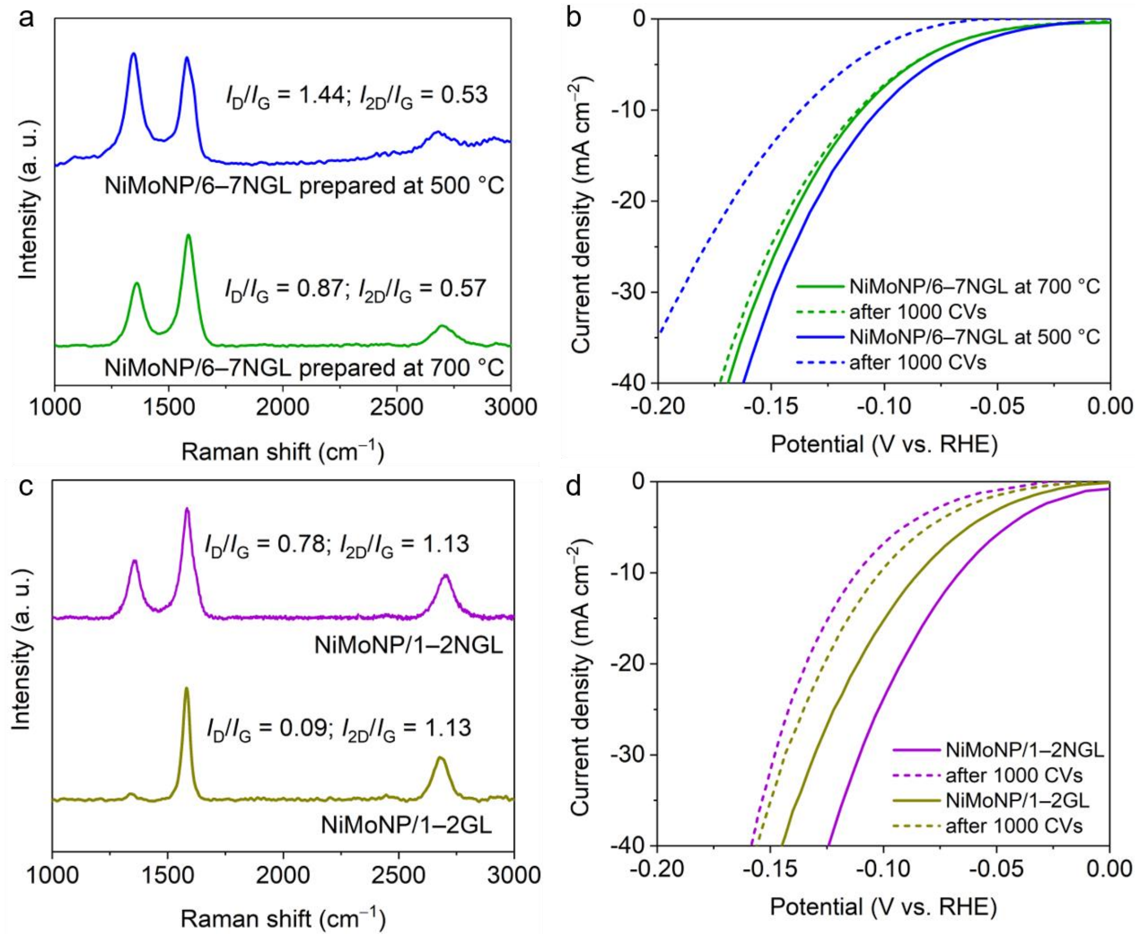

Supplementary Figure 34. Influences of graphene defects toward HER activities of graphene-covered NiMoNP samples. (a) Raman spectra and (b) HER performances of NiMoNP/6−7NGL samples prepared at 500 and 700 °C. (c) Raman spectra and (d) HER performances of NiMoNP/1−2NGL and NiMoNP/1−2GL samples. The loading amount of both catalysts: 5 mg.

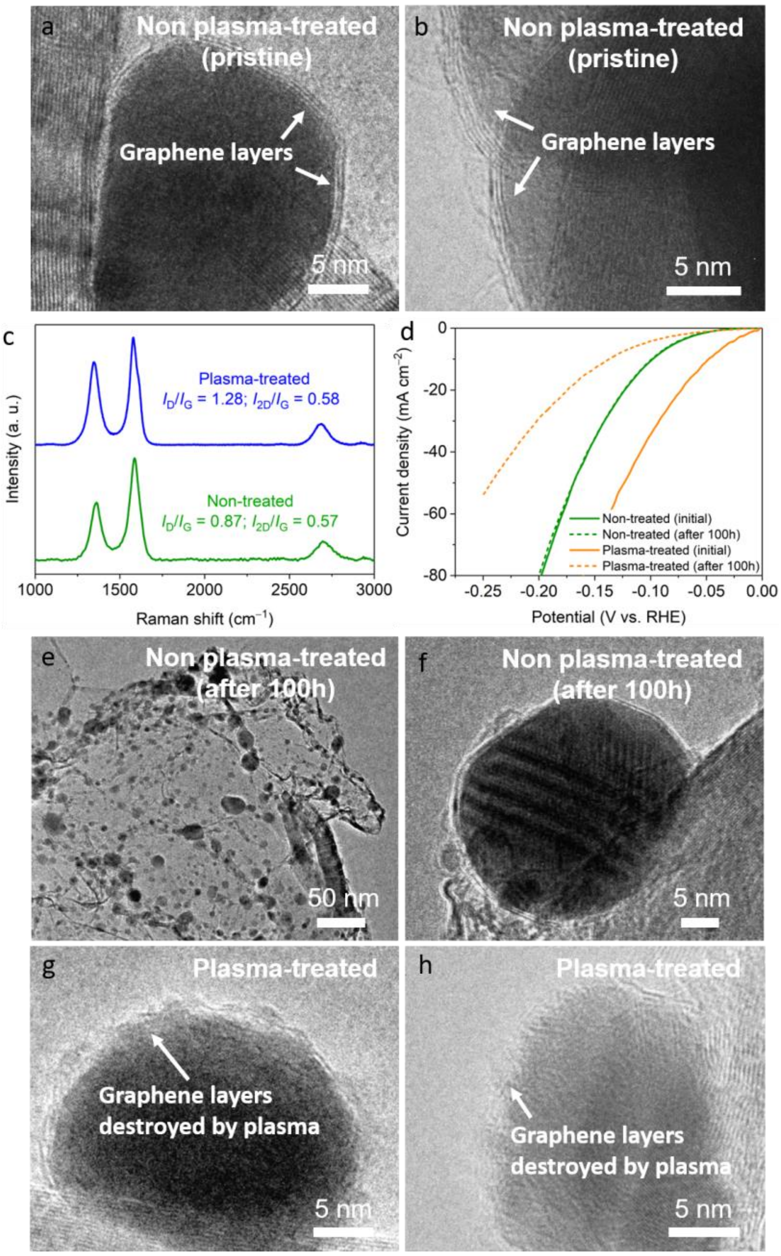

Supplementary Figure 35. Characterizations and HER activities of the NiMoNP/6-7 sample with and without oxygen plasma treatments. TEM images of the as-prepared/non-plasma-treated NiMoNP/6-7NGL sample (a, b) before and (e, f) after 100 h soaking in 0.5M H_2SO_4. (c) Raman spectra of non- and plasma-treated NiMoNP/6-7NGL samples. (d) HER polarization curves of the non- and plasma-treated NiMoNP/6-7NGL samples before and after 100 h soaking in 0.5M H_2SO_4. Dashed curves represented that the samples were measured after soaking in 0.5M H_2SO_4 for 100 h at 20 ºC. During the 100 h soaking, the samples were immersed in 0.5 M H_2SO_4 electrolyte. (g, h) TEM images of plasma-treated NiMoNP/6-7NGL samples.

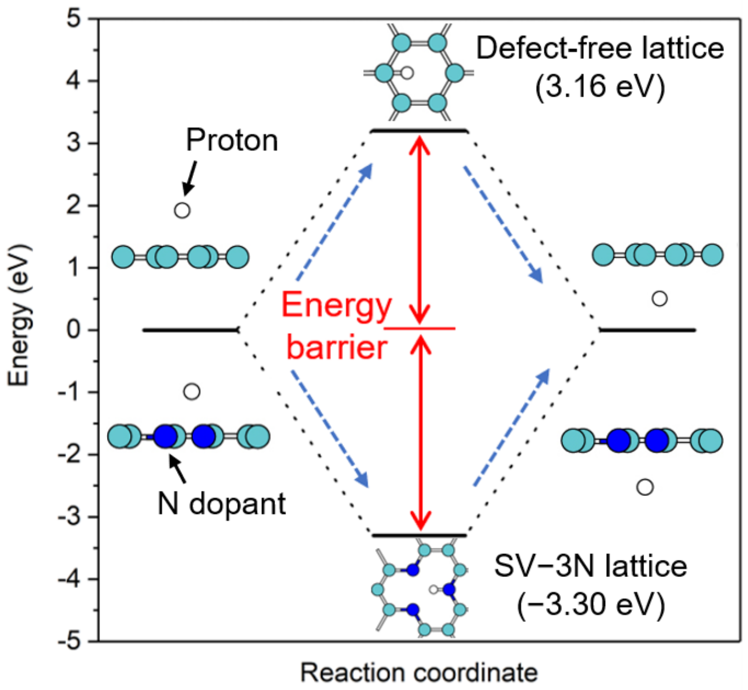

Supplementary Figure 36. Energy diagram of proton penetration through defect-free and SV-3N graphene lattices. The whole process that a proton penetrates through a graphene lattice can be divided into two steps: (1) the adsorption of the proton on the graphene lattice; (2) the desorption of the proton from the graphene lattice. The necessary energy needed to be overcome for proton penetration is called "energy barrier". The negative energy value for "SV-3N" lattice means that the proton energetically prefers to adsorb on the lattice rather than penetration, therefore an energy of 3.30 eV is needed for desorption. As shown in the energy diagram, the energy barriers for defect-free lattice and SV-3N lattice were 3.16 eV and 3.30 eV, respectively. In addition, the positive or negative value means that the shape of the potential energy surface is peak-shape or trough-shape. Colour code: carbon (cyan), nitrogen (blue), and proton (white).

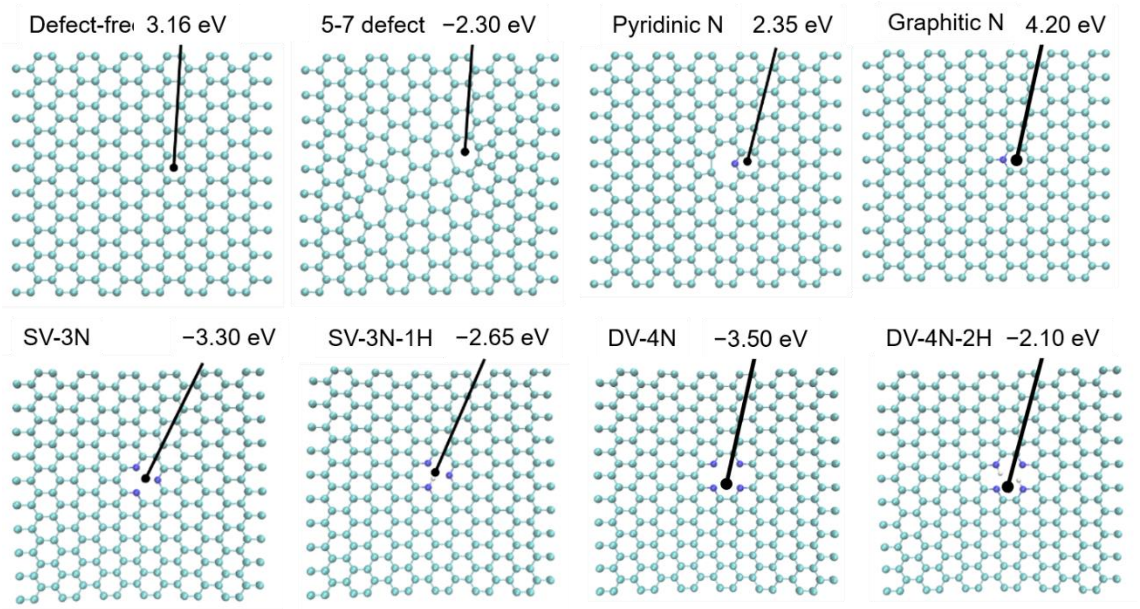

Supplementary Figure 37. Positions and energy barriers of proton penetration through different types of graphene lattice. The number represented the energy barrier for proton penetration. Colour code: carbon (cyan), nitrogen (blue), and proton (white).

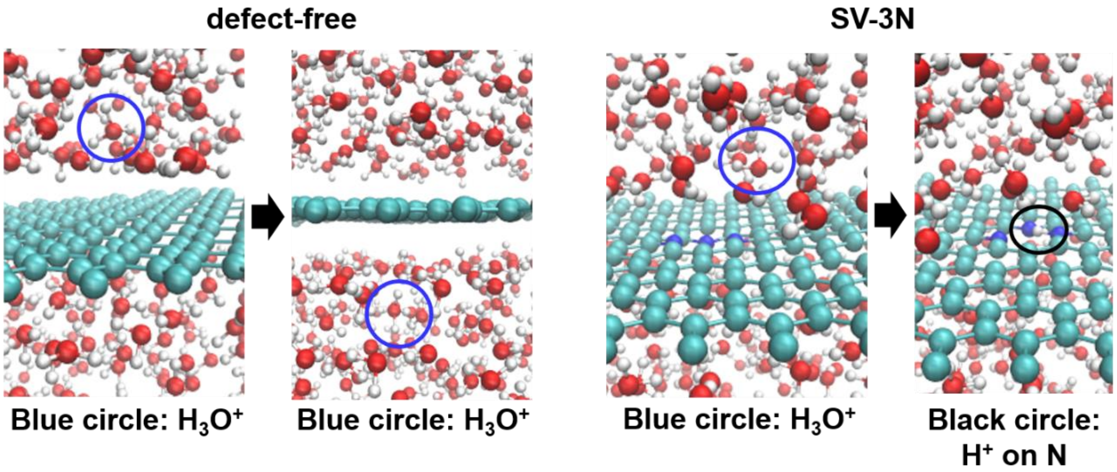

Supplementary Figure 38. Snapshots of proton penetration simulation of defect-free graphene and SV-3N graphene in presence of water molecules. The energy barriers of proton penetration through defect-free graphene (left) and SV-3N graphene lattice (right) in presence of water molecules were 2.97 and −1.93 eV. The blue and black circles indicated an H_3O^+ and a proton adsorbed on an N-dopant. Colour code: carbon (cyan), nitrogen (blue), oxygen (red) and hydrogen (white). Water molecules did not noticeably influence the energy barrier for proton penetration through the defect-free 1GL lattice; barrier reduction arises from the fact that the proton can more easily penetrated through the seven-membered carbon rings in the defect-containing graphene than the six-membered carbon rings in the defect-free graphene.

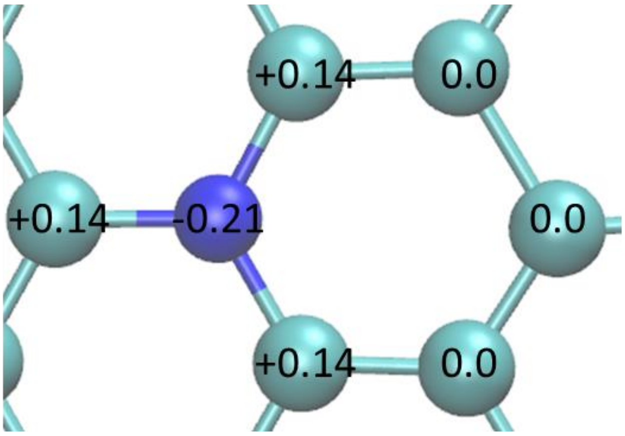

Supplementary Figure 39. Charge density distribution of N-doped graphene lattice. The numbers were the electric charges on C and N atoms. Colour code: carbon (cyan) and nitrogen (blue). The charges were in the unit of *e*.

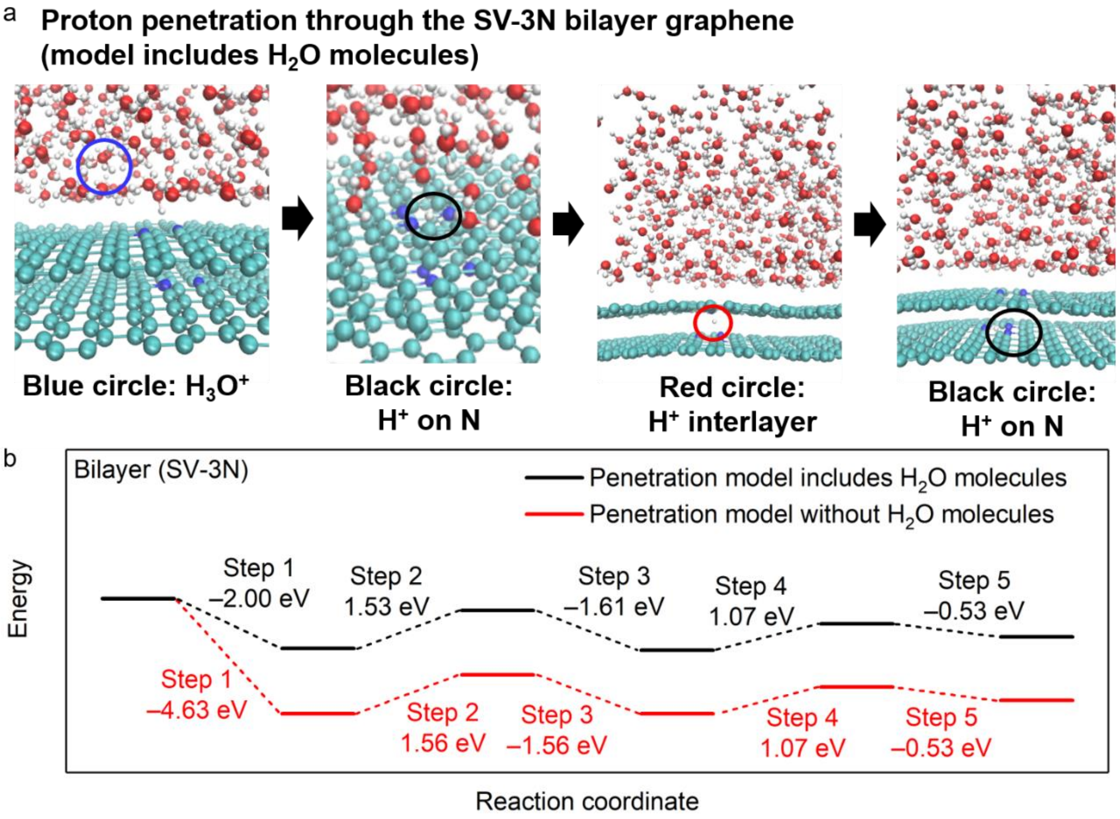

Supplementary Figure 40. Snapshots of the proton penetration simulation of SV-3N bilayer graphene in presence of water molecules. (a) The simulation models. This step corresponds to the steps 1−3 in Fig. 5c. The blue, red, and black circles indicated an H_3O^+, an intercalated proton, and a proton adsorbed on a N-dopant. Colour code: carbon (cyan), nitrogen (blue), oxygen (red) and hydrogen (white). (b) The energy diagrams of proton penetration through the SV-3N bilayer graphene in presence and absence of water molecules.

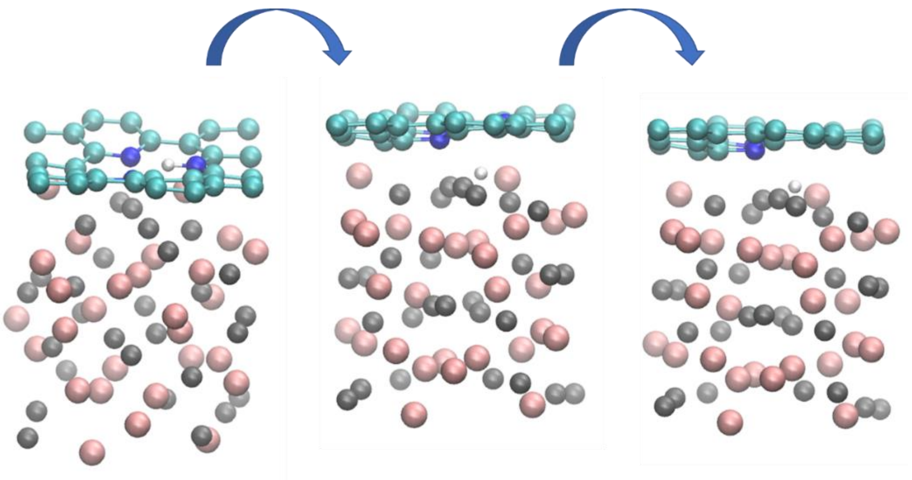

Supplementary Figure 41. Snapshots of the proton transfer from the graphene lattice to the NiMo surface. This step corresponds to the steps 4 and 5 in Fig. 5c. Colour code: carbon (cyan), nitrogen (blue), proton (white), nickel (grey), and molybdenum (pink).

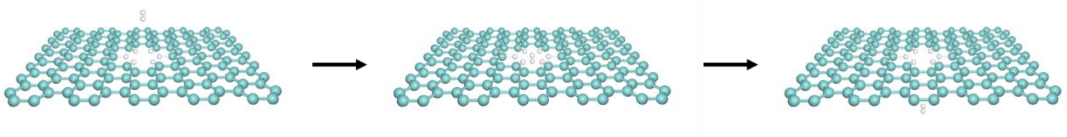

Supplementary Figure 42. H_2 penetration through the monolayer non-doped graphene lattice with a nanopore. Six hydrogen atoms adsorbed on the carbon atoms along the edge of the nanopore, in which the penetration energy barrier can be further reduced to 0.68 eV. Colour code: carbon (cyan) and proton (white).

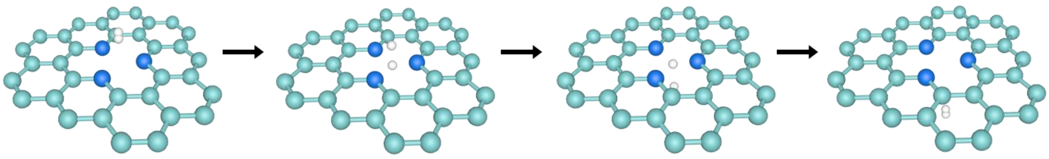

Supplementary Figure 43. H_2 penetration through the SV-3N graphene lattice by a decomposition−recombination process. This two-step sequence of decomposition and recombination largely reduced the overall energy barrier of H_2 penetration to 1.15 eV. Colour code: carbon (cyan), nitrogen (blue), and proton (white).

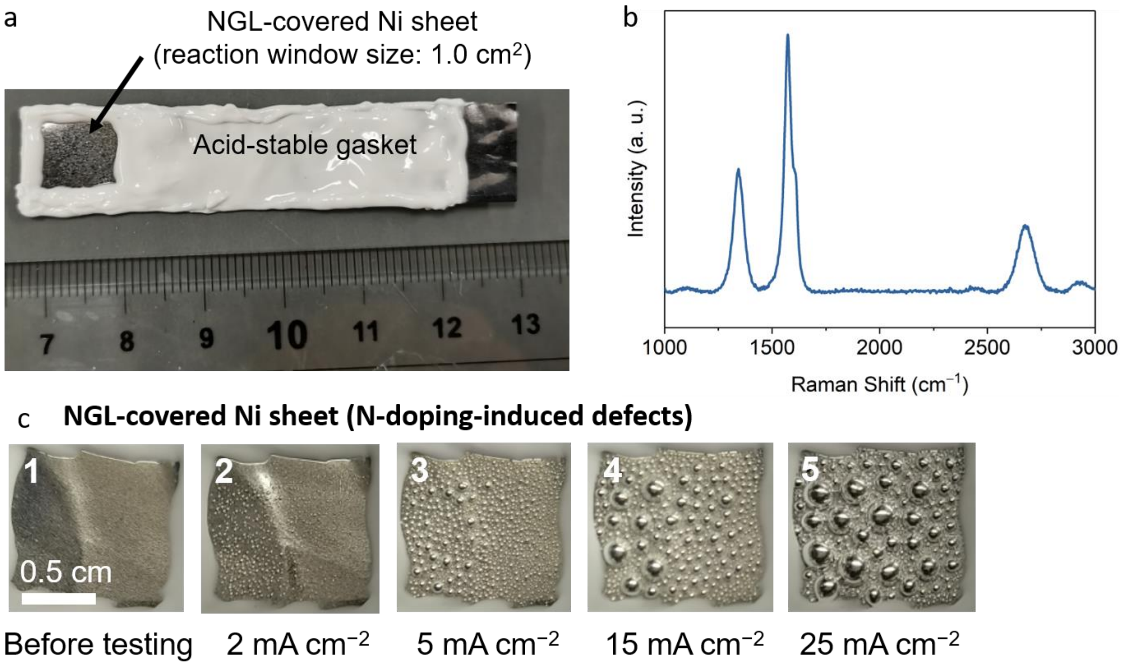

Supplementary Figure 44. Time dependence of hydrogen bubble formation on N-doped graphene (NGL)-covered Ni sheet. (a) Configuration of Ni sheet electrode covered by an insulator gasket. (b) Raman spectra of the N-doped graphene-covered Ni sheet. (c) Current density dependence of bubble formation on the NGL-covered Ni sheet.

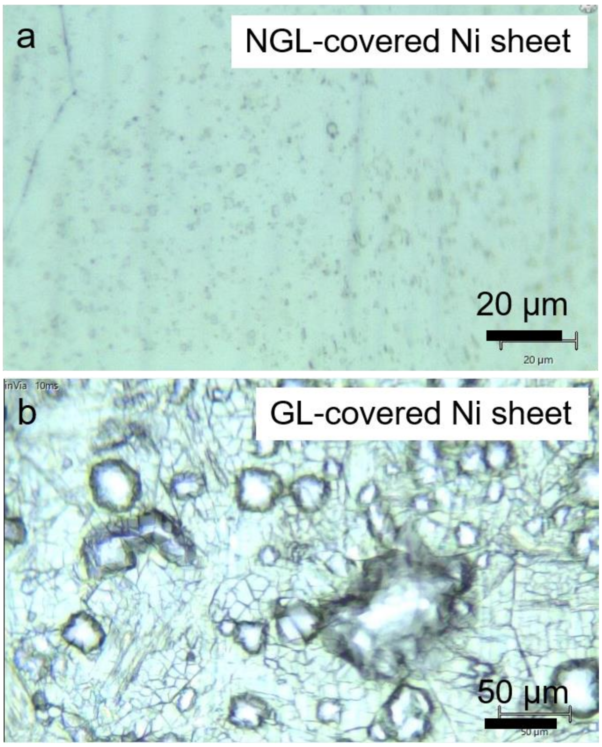

Supplementary Figure 45. H_2 Bubble formation of (a) NGL- and (b) GL-covered Ni sheets during HER process. The linear scan was carried out in the range from 0.0 to −0.2 V vs. RHE with a slow scan speed of 0.01 mV/s. The bubbles formed in the interface between Ni and GL, which were encapsulated by GL. In contrast, no encapsulated H_2 bubbles were observed in the NGL-covered Ni sheet.

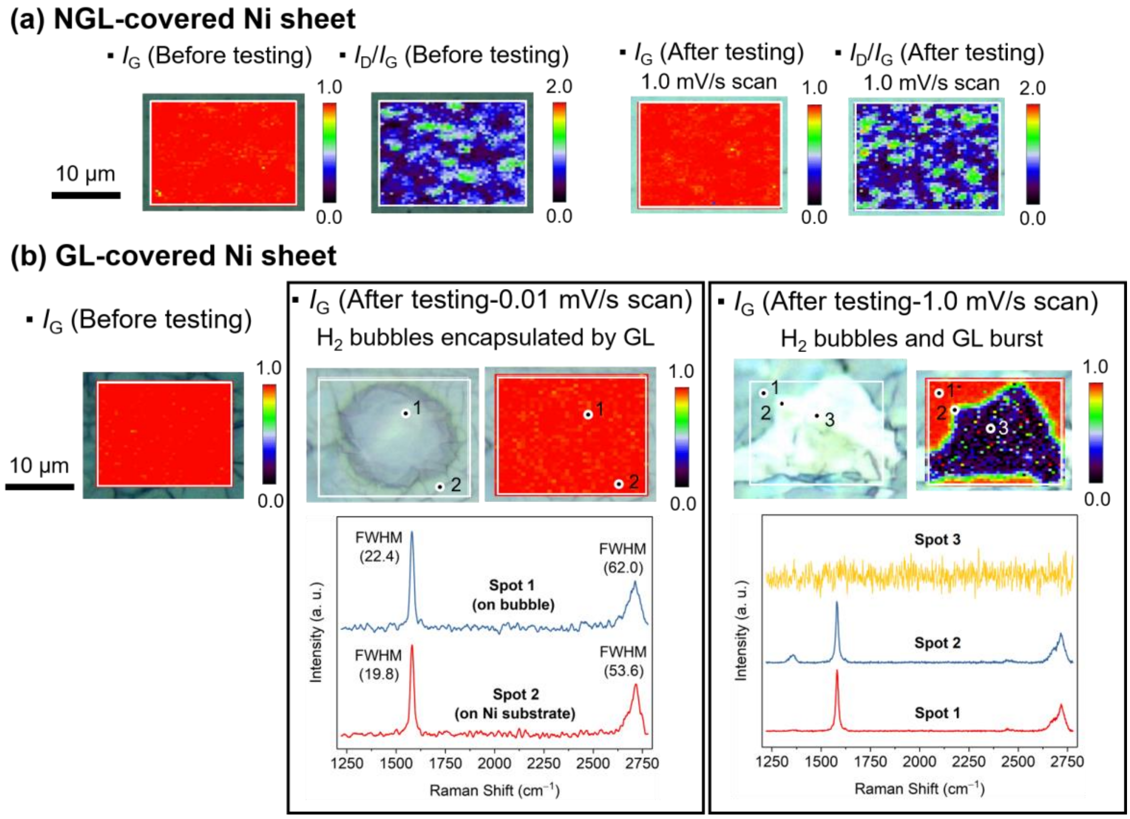

Supplementary Figure 46. Raman spectrum and mapping images of (a) NGL- and (b) GL-covered Ni sheets before and after testing in 0.5 M H_2SO_4 electrolyte. For the I_G mapping results, the *G* band intensity of all Raman spectra was normalized to 1. The full width at half maximum (FWHM) values of *G* and *2D* bands of the GLs on encapsulated H_2 bubble (Spot 1) and Ni substrate (Spot 2) were presented.

Supplementary Table 1. The rates of "red", "yellow" (spot 2), and "blue" (spot 1) areas in I_{2D}/I_G Raman map (Supplementary Fig. 5) of 1GL.

**Area（in I_{2D}/I_G image）**	**Layer Number**	**Rate (%)**
Red	Monolayer	98.1
Yellow (spot 2) and Blue (spot 1)	Bi- or tri-layer	1.9

Supplementary Table 2. Comparison of HER overpotentials at 10 mA cm^{-2} (η_{10}) of the bare graphene layers and the graphene-covered Cu and Ni sheets.

**Catalyst**	**η_{10} value (mV vs. RHE)**
Bare 3NGL	1684
Bare 6NGL	1341
Cu/3NGL	1078
Cu/6NGL	1254
Ni/3NGL	786
Ni/3GL	916
Ni/6NGL	942

Supplementary Table 3. Comparison of proton conductivity values for proton penetration through monolayer non-doped graphene.

Proton conductivity and conditions	Experimental energy barrier (eV)	Reference
0.16-0.6 mS cm^{-2} at 20 °C	0.95 ± 0.03	This work
~2 mS cm^{-2} at 21−23 °C	0.78 ± 0.03	Nature, 516, 227-230 (2014)
26.3 mS cm^{-2} at 30 °C	0.50 ± 0.05	Electrochim. Acta, 296, 1 (2019)
125 mS cm^{-2} at 60 °C	0.50 ± 0.05	Electrochim. Acta, 296, 1 (2019)
29 mS cm^{-2} at 30 °C	Not reported	J. Am. Chem. Soc., 140, 1743 (2018)
~4 mS cm^{-2} at room temperature	Not reported	ACS Nano, 13, 12109 (2019)

Supplementary Table 4. Electrical resistances related to the proton penetration through graphene with various layer numbers.

Separating membrane	Impedance R_1 value (Ω)
1GL	95.3×10^3
2GL	147.3×10^3
3GL	158.1×10^3
Nafion	5.3×10^3

Supplementary Table 5. Experimental values of energy barrier of proton penetration through various types of graphene.

Graphene sample	Energy barrier (eV)	Reference
1GL	0.95 ± 0.03	This work
2GL	1.76 ± 0.04	This work
1NGL	0.87 ± 0.03	This work
3NGL	1.17 ± 0.02	This work
6NGL	1.42 ± 0.02	This work
1GL	0.78 ± 0.03	Nature, 516, 227 (2014)

Supplementary Table 6. Detection of deuterium ions in m/z 98−100 after the 8 h CA testing.

Sample	*m/z*	Area	Observed abundance	Theoretical abundance from NIST database
trial 1	98	21879217	1.0000	1.0000
	99	255814	0.0117	0.0096
	100	916193	0.0419	0.0530
trial 2	98	48385210	1.0000	1.0000
	99	882144	0.0182	0.0096
	100	2369586	0.0490	0.0530

Note: Potential molecules were considered as m/z 98 for $H_2{}^{32}SO_4$, m/z 99 for $HD^{32}SO_4$ or $H_2{}^{33}SO_4$ and m/z 100 for $D_2{}^{32}SO_4$ or $H_2{}^{34}SO_4$. Considering the increased value of m/z 99, $HD^{32}SO_4$ molecules were detected in the cathode chamber after the 8 h CA testing. The theoretical abundance values were referred from NIST database.

Supplementary Table 7. Detection of deuterium ions in m/z 18−19 after the 8 h CA testing.

Sample	*m/z*	Area	Observed abundance	Theoretical abundance
trial 1	18	160727312	1.0000	1.0000
	19	1334031	0.0083	0.0006

Note: Potential molecules were considered as m/z 18 for H_2O and m/z 19 for HDO or $H_2{}^{17}O$. Considering the increased value of m/z 19, HDO molecules were detected in the cathode camber after the 8 h CA testing. The theoretical abundance values were referred from NIST database.

Supplementary Table 8. HER performances of the graphene-covered NiMoNP, bare NiMo alloy, and commercial Pt/C catalysts.

**Catalysts**	**η_{10} (mV)**	**Tafel slope (mV/dec)**	**η_{10} increase after 1000 CVs**	**Ni/Mo dissolution after 1000 CVs**
Pt/C	71	34	–	–
Bare NiMo alloy	27	35	800%	–
NiMoNP/1–2GL	84	61	20.2%	48.3 at%/16.9 at%
NiMoNP/1–2NGL	64	57	70.3%	26.6 at%/11.1 at%
NiMoNP/3NGL	80	60	13.7%	4.2 at%/1.6 at%
NiMoNP/6–7NGL	110	64	0.90%	1.5 at%/0.3 at%

Supplementary Table 9. DFT-calculated values of the energy barrier for the H atom penetration through the non-doped graphene with various methods.

**DFT methods**	**Energy Barrier (eV)**
BLYP-DZVP-D3	3.84
BLYP-MOLOPT-DZVP-D3	4.01
BLYP-MOLOPT-TZV2P-D3	4.06
PBE-DZVP	3.88
PBE-DZVP-D3	3.83

Supplementary Table 10. DFT-calculated values of the energy barrier for the proton penetration through the non-doped graphene in published literatures.

Graphene layer number	Energy barrier (eV)	Reference
Monolayer (no defect)	3.16	This work
Monolayer (no defect)	2.8	Nanoscale, 10, 5350 (2018)
Monolayer (no defect)	3.9	Nat Commun, 6, 6539 (2015)
Monolayer (no defect)	1.56	2D Mater., 3, 025004 (2016)
Monolayer (no defect)	1.25−1.40	Nature, 516, 227 (2014)
Monolayer (no defect)	1.17	New J. Phys. 12, 125012 (2010)
Monolayer (no defect)	1.41	Phys. Chem. Chem. Phys. 15, 16132–16137 (2013)

Supplementary Table 11. DFT-calculated values of the energy barrier for a molecular hydrogen penetration through monolayer graphene in published literatures.

Type of defects on monolayer graphene	Energy barrier (eV)	Penetration mechanism	Reference
Non-doped graphene (5-7 defect)	5.4	Direct penetration	This work
Non-doped graphene (5-8-5 defect)	4.6	Direct penetration	This work
Non-doped graphene (no defect)	4.8	H_2 decomposition−H atom penetration−recombination	This work
Non-doped graphene (SV-3N defect)	1.15	H_2 decomposition−H atom penetration−recombination	This work
Non-doped graphene (with a pore diameter of 3.5 Å)	0.68	Direct penetration	This work
Non-doped graphene (with a pore diameter of 2.5−3.0 Å)	0.025−0.22	Direct penetration	Nano Lett., 9, 4019, 2009
Non-doped graphene (with an angstrom-sized pore)	1.12	Direct penetration	Nature Nanotech, 10, 785 (2015)
Non-doped graphene (with a pore diameter of 7.4 Å)	0.54	Direct penetration	Carbon, 54, 359 (2013)
Non-doped graphene (with a pore diameter of 3.5 Å)	0.30−0.41	Direct penetration	J. Phys. Chem. C, 118, 19172 (2014)
Non-doped graphene (with a pore diameter of 5.8 Å)	0.09−0.25	Direct penetration	J. Phys. Chem. C, 118, 19172 (2014)
Non-doped graphene (with a pore diameter of 3.6 Å)	0.12	Direct penetration	Surface Science, 607, 153 (2013)
Non-doped graphene (with a pore diameter of 3.7 Å)	0.28−0.58	Direct penetration	Phys. Chem. Chem. Phys., 14, 13292 (2012)
N-doped graphene (with a pore diameter of 3.7 Å)	-0.17− -1.66 (kcal/mol)	Direct penetration	Phys. Chem. Chem. Phys., 14, 13292 (2012)

References

1. Chen, G. et al. Effect of calcination temperatures on the electrochemical performances of nickel oxide/reduction graphene oxide (NiO/RGO) composites synthesized by hydrothermal method. *J Phys Chem Solids* **98**, 209–219 (2016).

2. Kresse, G. & Hafner, J. Norm-conserving and ultrasoft pseudopotentials for first-row and transition elements. *J. Phys. Condens. Matter* **6**, 8245 (1994).

3. Blöchl PE. Projector augmented-wave method. *Phys. Rev. B* **50**, 17953 (1994).

4. Perdew, J. P., Burke, K. & Ernzerhof, M. Generalized gradient approximation made simple. *Phys. Rev. Lett.* **77**, 3865 (1996).

5. Grimme, S., Antony, J., Ehrlich, S. & Krieg, H. A consistent and accurate ab initio parametrization of density functional dispersion correction (DFT-D) for the 94 elements H-Pu. *J. Chem. Phys.* **132**, 154104 (2010).

6. Grimme, S., Ehrlich, S. & Goerigk L. Effect of the damping function in dispersion corrected density functional theory. *J. Comput. Chem.* **32**, 1456–1465 (2011).

7. Hu, K. et al. Graphene layer encapsulation of non-noble metal nanoparticles as acid-stable hydrogen evolution catalysts. *ACS Energy Lett.* **3**, 1539–1544 (2018).

8. Shoemaker, C. & Shoemaker, D. The crystal structure of the δ phase Mo–Ni. *Acta Crystallogr.* **16**, 997–1009 (1963).

9. Wang, Y. et al. Structural stability of Ni–Mo compounds from first-principles calculations. *Scr. Mater.* **52**, 17–20 (2005).

10. Ohto, T., Tada, H. & Nagata, Y. Structure and dynamics of water at water–graphene and water–hexagonal boron-nitride sheet interfaces revealed by ab initio sum-frequency generation spectroscopy. *Phys. Chem. Chem. Phys.* **20**, 12979–12985 (2018).

11. Shi, L., Xu, A., Chen, G. & Zhao, T. Theoretical understanding of mechanisms of proton exchange membranes made of 2D crystals with ultrahigh selectivity. *The J. Phys. Chem. Lett.* **8**, 4354–4361 (2017).

12. Soler, J. M. et al. The SIESTA method for ab initio order-N materials simulation. *J. Phys. Condens. Matter* **14**, 2745 (2002).

13. Deng, J., Ren, P., Deng, D. & Bao, X. Enhanced electron penetration through an ultrathin graphene layer for highly efficient catalysis of the hydrogen evolution reaction. *Angew. Chem. Int. Ed.* **54**, 2100–2104 (2015).

14. Sun, P. et al. Limits on gas impermeability of graphene. *Nature* **579**, 229–232 (2020).

15. Malard, L., Pimenta, M. A., Dresselhaus, G. & Dresselhaus, M. Raman spectroscopy in graphene. *Phys. Rep.* **473**, 51–87 (2009).
