# Supplementary material for: Catalytic activity of graphene-covered non-noble metals governed by proton penetration in electrochemical hydrogen evolution reaction
Source: Nat Commun. 2021 Jan 8;12:203. doi: 10.1038/s41467-020-20503-7 (PMC7794435; doi:10.1038/s41467-020-20503-7)
Supplement: Supplementary file 2 — Peer Review File [file 41467_2020_20503_MOESM2_ESM.pdf]

Reviewers' comments:

Reviewer #1 (Remarks to the Author):

This manuscript presents a proton penetration effect on graphene-encapsulated non-precious-metal catalyst for HER. The authors deal with the number of encapsulating graphene layers and the degree of defects for chemical corrosion resistance and HER catalytic activity via the experimental and modeling studies. This reviewer does not believe the current manuscript contain enough experimental evidences and significant contents to be considered for publication in Nat. Commun..

My major criticisms to the manuscript are given in below:

Focusing on the competition part (activated-surface of graphene vs. proton-penetration process). To address this issue, the authors investigated the HER activities of the 6-7 N-doped layers graphene with or without the NiMo substrates.
(a) How to prepare the 6-7 NGL sample without NiMo substrates?
(b) Why chose 6-7 NGL samples for this comparison?
(c) From the cited reference [13, Angew. Chem. Int. Ed. 2015, 54, 2100] in this manuscript, the electron penetration effect of the encapsulated metal cores on the graphene layers will decline when the graphene shell consists of more than three layers. Therefore, for the 6-7NGL sample, activation of the outmost graphene layer is quite difficult. In addition, the inner metal cores (such as NiMo here) should affect the activity via the electron transfer effect to activate the outmost graphene layer.

This manuscript focused on the proton penetration effect via the systematic experimental and modeling studies. However, the authors seemed to ignore the role of the NiMo cores except for the DFT calculations. If the proton can penetrate through the graphene layers to the NiMo metal surface and then generate hydrogen, the diffusion of the generated hydrogen under the cover between graphene layer and metal surface is a vital issue that need to be carefully considered.

Is this proton penetration effect universal for the other non-noble-metal cores? Since MoxC species are easily formed during the carbonization process. The valance state of NiMo metal cores should be presented.

For the preparation of the graphene-encapsulated NiMo samples, the authors chose 700 oC as the carbonization temperature. Since lower degree of graphene and higher density of defect using the lower carbonization temperature. Varying the carbonization temperature may be a way to verify the proton penetration effect of density of defects.

Reviewer #2 (Remarks to the Author):

Authors already reported graphene encapsulated NiMo nanoparticles for hydrogen evolution reaction (ACS Energy Letters 3, 1539-1544 (2018).) and oxygen evolution reaction (ACS Catal. 2020, 10, 1, 792-799). Now they use the same system and try to claim HER activity is determined by proton transfer through graphene layers, unfortunately without direct evidences. And the present work does not provide new insights about graphene encapsulated metal catalysts. Moreover current results could not fully support the conclusion due to lack of appropriate experiments. So I do not recommend its publication in the journal.

1 Energy barriers for proton transfer estimated by DFT calculations are 3.16 eV for perfect graphene and about 2.35 eV even for monolayer N-doped graphene. If it is true, how about the energy barrier for 6-7 layer N-doped graphene. It will be over 10 eV!!! based on the pioneering work (Nature 516, 227-230 (2014)) showing proton transfer barrier is linear change with layer number. That is a huge

energy barrier. In this situation, hydrogen evolution would take place on graphene surface NOT uncovered metal surface, which is OPPOSITE to the conclusion from experiments. So authors are encouraged to recalculate those energy barriers by adding factors, such as chemical enviorment, and also compare with proton transfer activation barriers experimentally estimated by Arrhenius plots.

2 In Figure 4d, authors plotted proton transfer resistance verse HER overpotentials on different samples, which is the key data to correlate the relation between HER catalytic activity and proton penetration through the graphene layers. However, they used different graphene materials in these experiments. CVD graphene grown on copper was transferred using Fe(NO3)3 solution to etch off copper substrate, and the resultant samples were used to investigate proton transfer resistance, while CVD Graphene covered NiMo samples were employed as HER catalysts. These two samples are quite differences in defects and morphologies, which will give a misleading relation or conclusion. So authors needs to redesign their experiments to find out direct evidences showing HER activity is determined by proton transfer.

3 The unit for current in Figure 4a is not correct. And HER activity on Pt needs to remeasure.

Reviewer #3 (Remarks to the Author):

In this work, Hu et al report two sets of measurements. First, they measure the HER activity of a non-noble metal catalyst coated with few-layered graphene. The number of graphene layers on the catalyst can be controlled with reasonable accuracy. Second, they measure proton transport through stacks of these graphene layers using micro-fabricated suspended graphene/Nafion devices. The number of graphene layers can be controlled exactly. Both the catalytic activity and the proton transport results were, to a large extend, measured in previous works. The novelty of this work is that the authors demonstrate that the activity of the catalyst as a function of graphene layers correlates perfectly with the proton conductivity through these layers. This is strong experimental evidence showing that the HER activity of the graphene-coated catalysts is governed by proton transport. This represents a shift in how these catalysts are understood and it came as a surprise. All previous works – including some by the authors themselves (ref. 8) – suggested that the HER on graphene coated catalysts took place on the surface of the graphene coating. The work has been carefully done and the manuscript is well written.

The main concern with the present work is the exit of H2 molecules from the graphene-coated catalyst. The authors propose two exit mechanisms. One is that these molecules diffuse through nanometre (<8 A) pores in the lattice. Since the catalysts in this work are normally only a couple nanometres across, if such pores were present, a large proportion of their catalysts would not be covered by graphene. This would seem to contradict their finding that the graphene protects it against corrosion. Furthermore, their OCV measurements imply that their membranes are nearly perfectly selective between protons and anions in their electrolyte solution (L. Mogg et al. Nat. Comms. 4243, 2019). This is not possible if nanopores are present. The second proposed mechanism is that the H2 molecules split into two H atoms, diffuse through vacancies and recombine after transferring through the graphene coating. While this seems plausible, the authors should explain why this process does not limit their HER activity. This second recombination, which takes place without a catalyst, could be expected to be slower than the one on their catalyst. In any case, this discussion should be in the main text, rather than in Supplementary.

One minor comment.
The authors report isotope experiments, but do not compare their results with their proton data. This would be of interest to the community.

If the authors can address these comments, I would like to recommend for publication.

Reviewers' comments:

Reviewer #1 (Remarks to the Author):

This manuscript presents a proton penetration effect on graphene-encapsulated non-precious-metal catalyst for HER. The authors deal with the number of encapsulating graphene layers and the degree of defects for chemical corrosion resistance and HER catalytic activity via the experimental and modeling studies. This reviewer does not believe the current manuscript contain enough experimental evidences and significant contents to be considered for publication in Nat. Commun.

Reply: We appreciate reviewer's fruitful comments and spending time to improve our manuscript. We redesigned the experiments and theoretical approaches and drastically changed the contents to address your concerns.

My major criticisms to the manuscript are given in below:

Focusing on the competition part (activated-surface of graphene vs. proton-penetration process). To address this issue, the authors investigated the HER activities of the 6-7 N-doped layers graphene with or without the NiMo substrates.

(a) How to prepare the 6-7 NGL sample without NiMo substrates?

Reply: We performed a standard chemical vapor deposition (CVD) method to grow graphene on porous NiMo alloy substrates. After the CVD process, the NiMo substrates with their surface covered by graphene layers was etched by using the 2.0 M HNO_3 solution mixed with isopropanol (volume ratio of HNO_3 to IPA is 4:1) at 80 °C. After etching, the 6−7 NGL was isolated. More details were in the revised supplementary information.

(b) Why chose 6-7 NGL samples for this comparison?

Reply: The reason why we chose 6−7NGL samples was to exclude electron charge transfer effect from the encapsulated alloy cores to graphene layers. For 1−3 graphene layers covering case, Deng

et al (Angew. Chem. Int. Ed. 2015, 54, 2100–2104) reported that the electron could be transferred from CoNi alloy substrate to outermost graphene layers (three layers at most), which plays an important role in the electrochemical hydrogen evolution. On the other hands, for over 10 graphene layers covering, proton penetration phenomenon was not observed in our experimental results (**Figure 3e**). Based on these observations, we concluded that the 6−7 graphene layer is the best sample to investigate proton penetration effects on HER in absence of the charge transfer effect. Indeed, we observed that the NiMo nanoparticles covered by 6−7 N-doped graphene layers (NiMoNP/6−7NGL) showed much higher HER activity than the bare 6−7NGL sample without NiMo (**Figure R1**), which can be attributed to the proton penetration effect. We added this point to the revised supplementary information and **Figure R1** as **Figure S26**.

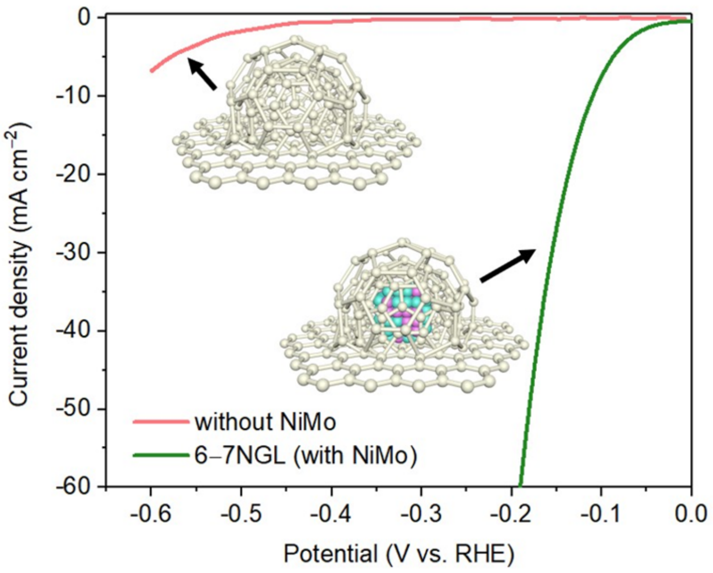

Figure R1. HER activity of the NiMoNP/6−7NGL sample and the bare 6−7NGL sample without NiMo.

(c) From the cited reference [13, Angew. Chem. Int. Ed. 2015, 54, 2100] in this manuscript, the electron penetration effect of the encapsulated metal cores on the graphene layers will decline when

the graphene shell consists of more than three layers. Therefore, for the 6-7NGL sample, activation of the outmost graphene layer is quite difficult. In addition, the inner metal cores (such as NiMo here) should affect the activity via the electron transfer effect to activate the outmost graphene layer.

Reply: We appreciate the reviewer's comments based on his/her deep understanding. To address this point, we redesigned the experiments to examine whether the charge transfer effect, which was ascribed to be a major source of the HER in the reference [13, Angew. Chem. Int. Ed. 2015, 54, 2100.], governs the HER activities of the graphene-covered non-noble-metal catalysts. The data is given in **Figure R2**. The results show that the proton penetration instead of the charge transfer is the main source for the HER currents. Furthermore, we clarified that, through the proton penetration, HER on the graphene-covered metal surface occurs predominantly, while the HER on the outmost graphene layer is rather minor. The details of our finding based on the newly performed measurements are described below.

N-doped graphene-covered Cu sheets (Cu as HER-less-active catalyst) and Ni sheets (Ni as HER-highly-active catalyst) were prepared for comparisons (**Figure R2a**, preparation details in supplementary information). Firstly, cyclic voltammetry (CV) of graphene-covered Cu sheets with 0NGL (i.e., bare Cu without graphene), 3NGL, and 6NGL were tested in 0.5 M H_2SO_4 electrolyte (**Figure R2b**). The Cu/3NGL showed 36.0% lower overpotential (η_{10}) at the current density 10 mA cm^{-2}, normalized by the geometric area of the electrode, than that of the bare 3NGL (i.e., without Cu sheets), while the Cu/6NGL showed only 6.5% than that of the bare 6NGL. In the reference [13, Angew. Chem. Int. Ed. 2015, 54, 2100.], the charge transfer from the metal substrate to the outermost graphene layers (around 3rd graphene layer) occurs so that we successfully observed the reported charge transfer effect in the graphene-covered Cu system, which is also a good agreement with the reviewer's statement "*Therefore, for the 6-7NGL sample, activation of the outmost graphene layer is quite difficult*". Next, CV of graphene-covered Ni sheets with 0NGL (i.e., bare Ni without graphene), 3NGL, and 6NGL were similarly tested (**Figure R2c**). If HER occurs on

graphene surface and the charge transfer effect mentioned in the reference [13, Angew. Chem. Int. Ed. 2015, 54, 2100.] is the sole determining factor in HER activities, the Ni system should show similar behavior with the Cu system regardless of the HER activity of the metal substrates (i.e., Ni: HER-highly-active metal; Cu: HER-less-active metal). However, we found that the Ni system exhibited completely different behavior compared with the Cu system. A significant improvement of the HER overpotential (η_{10}) of the Ni/3NGL in comparison to that of the bare 3NGL (from 1.68 to 0.78 V vs. RHE) was observed, and a similar difference was also exhibited between bare 6NGL and Ni/6NGL (from 1.34 to 0.94 V vs. RHE). These large overpotential differences cannot be solely explained by the charge transfer effect (**Figure R2d**), and the HER processes do not totally occur on the outermost graphene. To further confirm the finding that HER occurs on the metal surface covered by graphene layers, we applied a high electrode potential to the Ni/3GL and observed the generation of H_2 bubbles at the interface between Ni sheet and graphene layers (**Figures R2e**). Raman spectra at the spot marked by a red star evidenced that the generation of the bubbles occurred between the interface and there were no obvious breaks on the graphene layers (inset of **Figure R2f**). Thus, these differences of the overpotentials observed in the Cu and Ni systems (**Table R1**) suggest another HER mechanism, such as proton penetration, plays an important role in catalytic reaction. We added these discussions to the revised manuscript and **Figure R2** as **Figure 2**. In addition, we investigated the charge transfer effect on five N-doped graphene layers stacked NiMo alloy system by DFT calculations (ACS Energy Letters 2018, 3, 1539.). The charge transfer on the 5th graphene layer was only +0.014 e and it almost had no influence toward the charge distribution of the outermost graphene layer (**Figure R3**). Therefore, we assumed that there should be another effect, except the charge transfer, that determines the HER activity of the graphene-covered non-noble-metal catalysts. We added this point to the revised introduction.

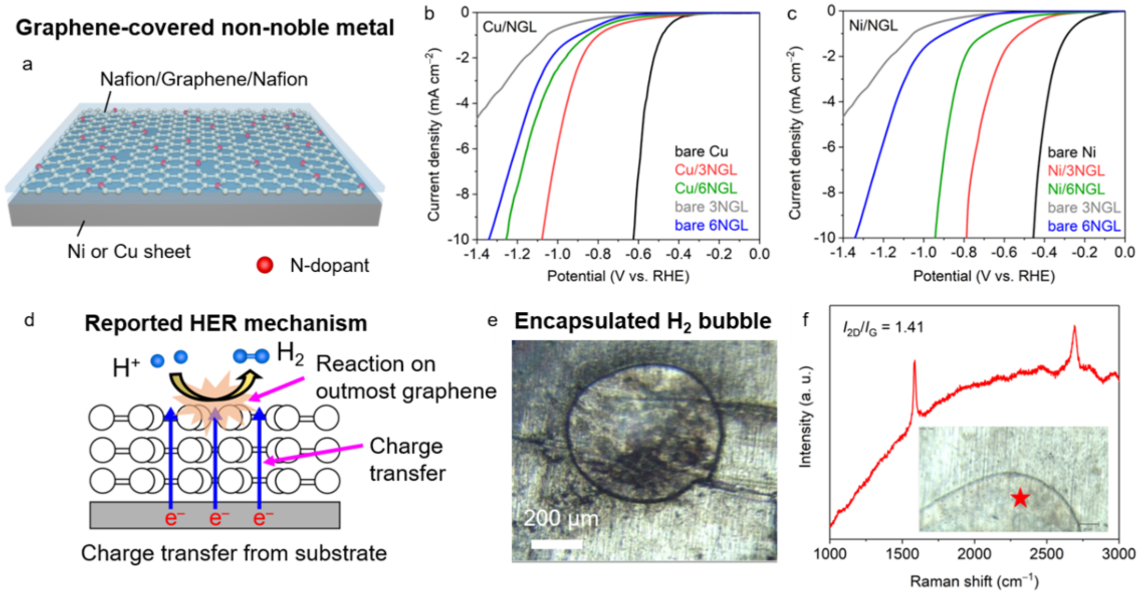

Figure R2. (a) Schematic diagram of the graphene-covered Ni or Cu sheet. HER polarization curves of the graphene-covered (b) Cu and (c) Ni sheets. (d) Reported HER mechanism of the graphene-covered metal/alloy catalysts by the charge transfer. (e) Optical photo of an encapsulated H_2 bubble between the surface of Ni sheet and the graphene layers. (f) Raman spectrum of the graphene layers on the bubble. Inset: the Raman measurement was obtained at the location indicated by the red star.

Table R1. Comparison of the η_{10} values of the graphene-covered Ni and Cu sheets.

Catalyst	η_{10} value (mV vs. RHE)
Bare 3NGL	1684
Bare 6NGL	1341
Cu/3NGL	1078
Cu/6NGL	1254
Ni/3NGL	786
Ni/3GL	916
Ni/6NGL	942

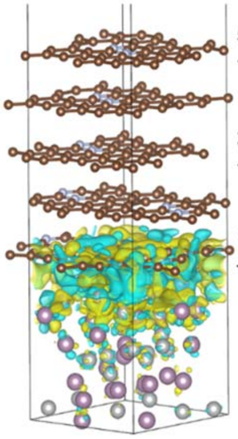

Figure R3. Charge density difference for NiMo(100) covered by five N-doped graphene layers (NGLs), with total charges of the first, third, and fifth NGLs on NiMo(100). Yellow and cyan areas represent electron accumulation and depletion.

This manuscript focused on the proton penetration effect via the systematic experimental and modeling studies. However, the authors seemed to ignore the role of the NiMo cores except for the DFT calculations. If the proton can penetrate through the graphene layers to the NiMo metal surface and then generate hydrogen, the diffusion of the generated hydrogen under the cover between graphene layer and metal surface is a vital issue that need to be carefully considered.

Reply: We agree with the reviewer that the diffusion of generated hydrogen is crucial. In the prior manuscript, we made discussions on the generated H_2 ejection through graphene layers in **Figures S38** and **S39**, while such discussions have been placed in the main text through revision of the manuscript. Moreover, graphene which grew on the curved substrates (e.g., nanoparticles or nanoporous structures) demonstrated the high defect density (Adv. Mater., 2016, 28, 10644−10651.; Angew. Chem. Int. Ed. 2018, 57, 13302.) and the large defects such as 7- or 8- membered rings could promote the ejection of generated hydrogen molecules. We added the discussions and the references to the revised manuscript.

Added discussion summary

The ejection of generated hydrogen molecules prefers to occur through nanopores (**Table S9**). Indeed, defect-free graphene layer confines the generated hydrogen molecules at the interface between Ni sheet and graphene layers (**Figure R2e**). In addition, the high density of structural defects is geometrically required to make the graphene lattice fit to the nanoparticle morphology, which plays an important role in ejecting hydrogen molecules through the defects on the curved graphene (refs. 27 and 28 in the revised manuscript).

Is this proton penetration effect universal for the other non-noble-metal cores? Since MoxC species are easily formed during the carbonization process. The valance state of NiMo metal cores should be presented.

Reply: We appreciate the important comments. To address the concern that "Is this proton penetration effect universal for the other non-noble-metal cores?", we further investigated HER activities of graphene-covered Ni sheet and Ni nanoparticles (NiNP) (see supplementary method and supplementary discussion 3, **Figures S21** and **S22**) in comparison to the graphene-covered NiMo nanoparticles (NiMoNP). We aim at revealing the influence of morphology (sheet vs. nanoparticle) and metal component (metal vs. alloy) toward the universality of proton penetration effect. The proton current ability (i.e., average 8-h CA proton current) and the overpotential ($\eta_{10\text{-tot}}$) at the current density 10 mA cm_{tot}^{-2}, normalized by the total surface area (i.e., the BET surface area of the catalyst), was plotted to fairly compare the intrinsic HER activity of various catalysts (**Figure R4**). We also found the linear correlation between catalytic activity and proton penetration through graphene layers in graphene-covered Ni sheets and NiNP/NiMoNP samples. These results reveal that the catalytic activity of the graphene-covered non-noble-metal catalysts is governed by proton penetration through the graphene layers, regardless of substrate morphology (sheet and nanoparticle) or composition (metal and alloy). We added this discussion and figure to the revised manuscript.

We investigated the formation of MoxC species in the graphene-covered NiMoNP samples. By turning the CVD temperature at 700 °C, we confirmed the valance state of NiMo metal cores by XRD and XPS measurements and no Mo_2C species was detected (**Figure R5**). Therefore, we concluded the metallic state of NiMo through these experiments. We added the **Figure R4a** and related discussions to the revised manuscript. **Figures R4b and R5** and related discussions were added to the revised supplementary information.

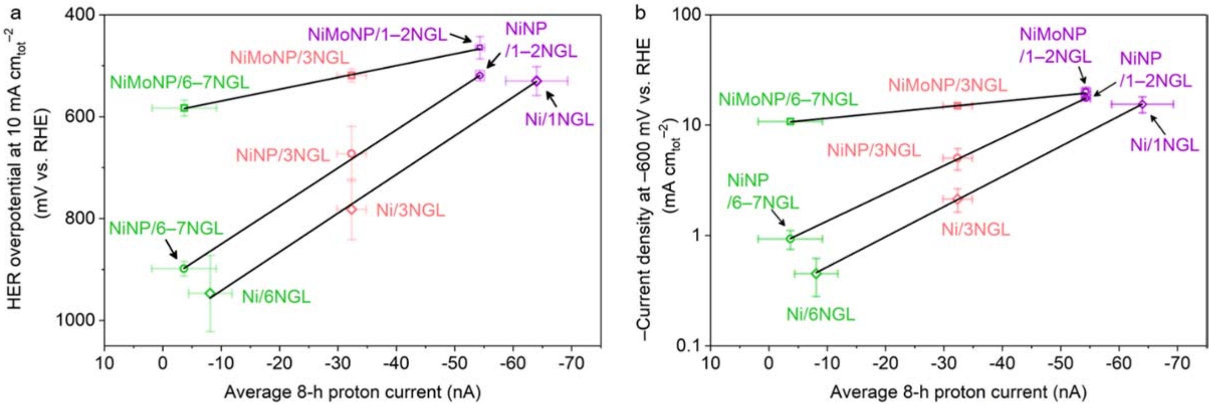

Figure R4. Correlation between catalytic activity and proton penetration through the graphene layers in the graphene-covered Ni sheet and NiMoNP/NiNP catalysts. (a) HER overpotential at the 10 mA cm_{tot}^{-2} plotted as a function of the proton current. (b) The current density at a cathodic potential of −600 mV vs. RHE in log scale plotted as a function of the proton current. The black lines are the fits to the correlations.

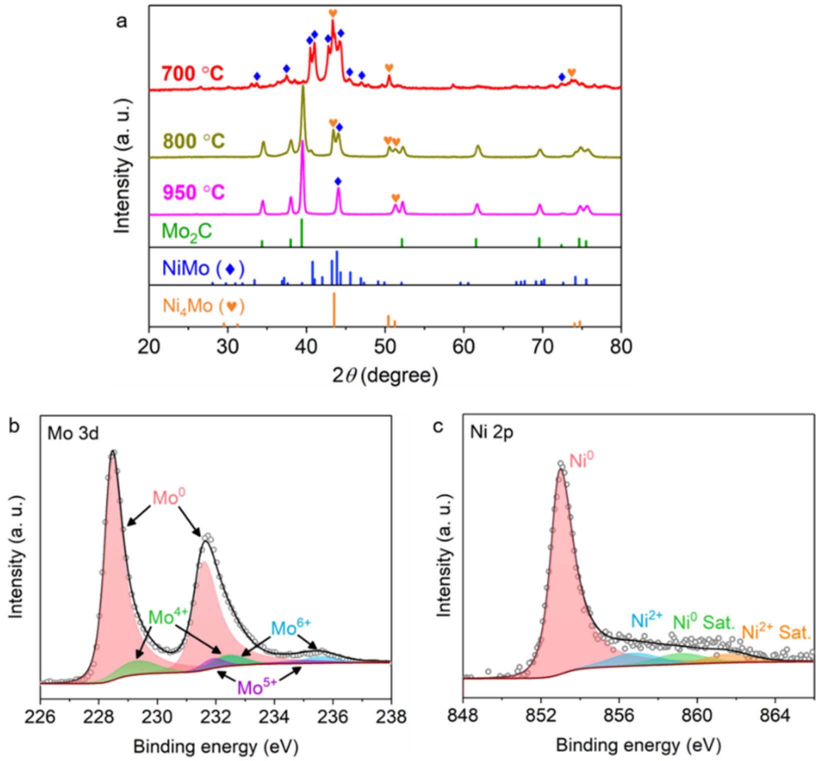

Figure R5. (a) XRD patterns of graphene-encapsulated NiMoNP samples prepared at different carbonization temperatures ranged from 700 to 950 °C. MoC_2 formation were confirmed at CVD temperature of 800 °C and 950 °C. XPS spectra of the NiMoNP sample prepared at 700 °C: (b) Mo 3d and (c) Ni 2p.

For the preparation of the graphene-encapsulated NiMo samples, the authors chose 700 ^{o}C as the carbonization temperature. Since lower degree of graphene and higher density of defect using the lower carbonization temperature. Varying the carbonization temperature may be a way to verify the proton penetration effect of density of defects.

Reply: We agree with this comment that the carbonization temperature plays an important role in the defect density of graphene, and the temperature variation is a good way to study the proton penetration effect of density of defects. As the discussion above (**Figure R5**), the Mo_2C readily formed as a predominant component at a carbonization temperature above 700 °C (i.e., 800 °C and 950 °C) although the lower density of defects on graphene were achieved. In other words, the

contribution of HER-active Mo_2C spices cannot be excluded from proton penetration experiments at the samples prepared at 800 °C and 950 °C. Therefore, we synthesized the sample with a higher defect density at a lower carbonization temperature of 500 °C. The higher density of defects (I_D/I_G = 1.44) on N-doped graphene was observed compared to the counterpart that prepared at 700 °C (I_D/I_G = 0.87) (**Figure R6a**). The HER activity of NiMoNP/6−7NGL prepared at 500 °C showed an improved initial HER activity (**Figure R6b**), owing to the high density of structural defects. However, the poor stability of the NiMoNP/6−7NGL prepared at 500 °C was also observed. This means that the higher defect density enhances both proton penetration and dissolution of NiMo through large defects on graphene, reducing the catalyst lifetime. Thus, the balance of defects plays an important role in HER performances (activity and catalyst lifetime). We added this discussion and **Figure R6** to the revised supplementary information.

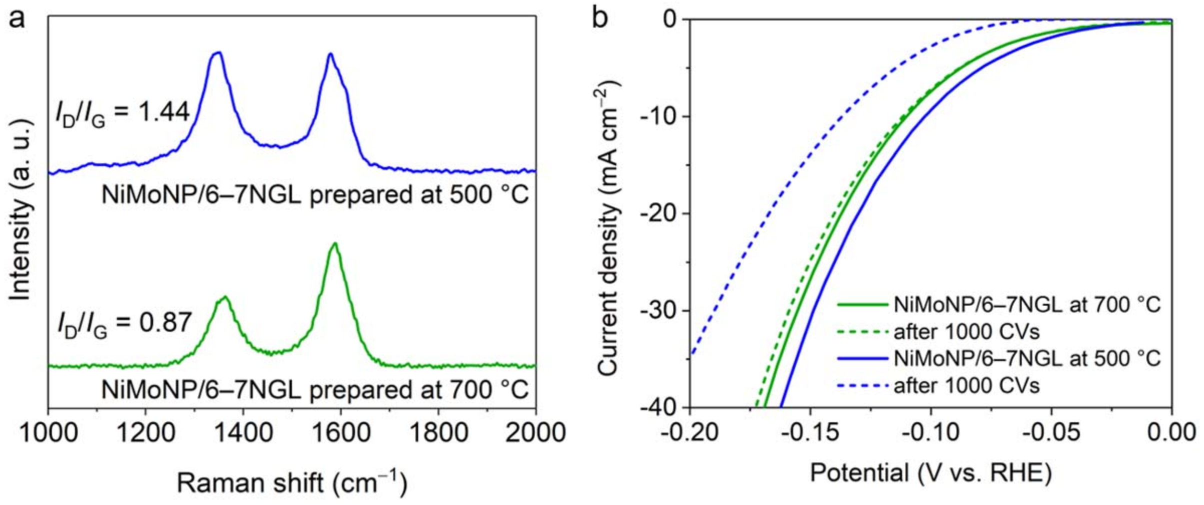

Figure R6. (a) Raman spectra and (b) HER polarization curves of NiMoNP/6−7NGL samples prepared at 500 and 700 °C, respectively. The current density was normalized by the electrode surface area.

Reviewer #2 (Remarks to the Author):

Authors already reported graphene encapsulated NiMo nanoparticles for hydrogen evolution reaction (ACS Energy Letters 3, 1539-1544 (2018).) and oxygen evolution reaction (ACS Catal. 2020, 10, 1, 792-799). Now they use the same system and try to claim HER activity is determined by proton transfer through graphene layers, unfortunately without direct evidences. And the present work does not provide new insights about graphene encapsulated metal catalysts. Moreover current results could not fully support the conclusion due to lack of appropriate experiments. So I do not recommend its publication in the journal.

Reply: We appreciate reviewer's fruitful comments. The reviewer's criticism contains two points – one is no direct evidence for HER mechanism and the other is no new insight into the catalysis. For the first point, we redesigned the experiments and theoretical approaches and drastically changed the manuscript for demonstrating the novelty that reveals the fundamental HER mechanism of graphene-covered non-noble-metal catalysts. For the second point, we respectfully disagree with the reviewer. In fact, the catalytic reaction arises from the charge transfer or proton penetration is under debate (reference #13: Angew. Chem. Int. Ed. 2015, 54, 2100.). Here, by combining the proton penetration experiments, electrochemical measurements of graphene-covered metal/alloy catalysts, and DFT calculations, we draw the universal relation between the proton penetration and the HER activity. Amazingly, the universal relation is applicable to non-noble-metal catalysts with different morphology and different compositions.

1 Energy barriers for proton transfer estimated by DFT calculations are 3.16 eV for perfect graphene and about 2.35 eV even for monolayer N-doped graphene. If it is true, how about the energy barrier for 6-7 layer N-doped graphene. It will be over 10 eV!!! based on the pioneering work (Nature 516, 227-230 (2014)) showing proton transfer barrier is linear change with layer number. That is a huge energy barrier. In this situation, hydrogen evolution would take place on

graphene surface NOT uncovered metal surface, which is OPPOSITE to the conclusion from experiments. So authors are encouraged to recalculate those energy barriers by adding factors, such as chemical environment, and also compare with proton transfer activation barriers experimentally estimated by Arrhenius plots.

Reply: We respectfully disagree with the reviewer's concerns. We experimentally estimated the energy barrier (activation energy) of proton penetration through monolayer, trilayer, and hexalayer of N-doped graphene (abbreviated as 1NGL, 3NGL, and 6NGL). The activation barrier of proton penetration was obtained by measuring Arrhenius-type plots as following the reviewer suggested reference (Nature 516, 227-230 (2014)). The proton conductivities show an Arrhenius-type behaviour, exp $(-E/k_BT)$, with the temperature ranged from 273 to 328 K (**Figure R6**), where k_B is the Boltzmann constant. The energy barrier E were calculated as 0.87, 1.17, and 1.42 eV for the 1NGL, 3NGL, and 6NGL, respectively. Experimental results indicate that the activation energy does not simply follow a linear change with the layer number of graphene. In addition, the reference (Nature 516, 227-230 (2014)) does not mention that the barrier linearly changes with the layer number. We added these results, discussions, and **Figure R7** to the revised manuscript.

Moreover, we recalculated the energy barriers of proton penetration through graphene with H_2O molecules as following the reviewer's comment. We found that the energy barrier of N-doped graphene (the SV-3N) lattice decrease from 3.30 to 1.93 eV at the presence of H_2O molecules. Furthermore, the energy diagram for proton penetration through a bilayer SV-3N graphene lattice (**Figure 5c**) showed that the energy barrier for the interlayer proton hopping is smaller than that for proton penetration through monolayer N-doped graphene. The reported energy barrier and our energy barrier were summarized in **Table R2**. Although the apparent activation energy by DFT calculation does not matched with the experimental activation energy, this mismatching is also explained in the reviewer suggested reference (Page 2, Nature 516, 227-230 (2014)). We added the new DFT calculation results in the revised manuscript and the comparison table in the revised

supplementary information.

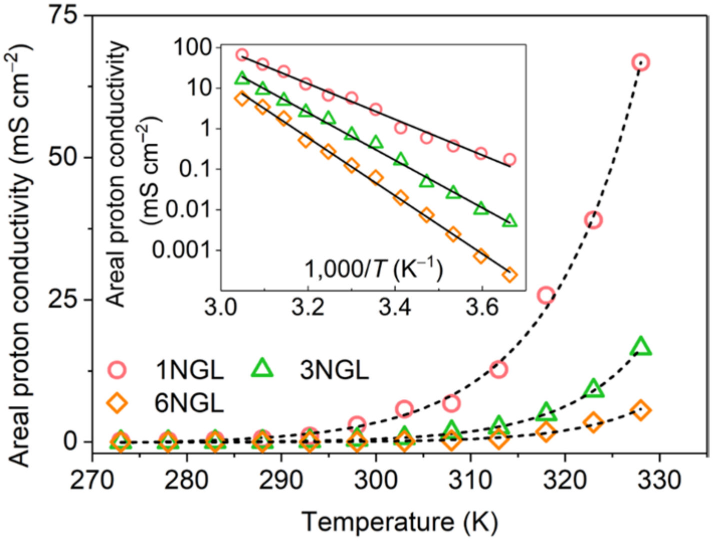

Figure R7. Temperature dependences of proton conductivity for 1NGL, 3NGL, and 6NGL. Inset: the log(σ) as a function of T^{-1} and the black lines present the linear fitting to estimate the activation energy.

Table R2. Comparison of the activation energy E values of proton penetration in this work and prior literature.

	This work	Nature, 516, 227-230 (2014)
Experimentally measured E	0.87 eV (1NGL)<br>1.17 eV (3NGL)<br>1.42 eV (6NGL)	0.78 eV<br>(non-doped graphene monolayer)
DFT-calculated E	1.93 eV(1NGL)	1.25-1.40 eV<br>(non-doped graphene monolayer)

Note: no E value of proton penetration through graphene more than monolayer has been published in Nature, 516, 227-230 (2014).

2 In Figure 4d, authors plotted proton transfer resistance verse HER overpotentials on different samples, which is the key data to correlate the relation between HER catalytic activity and proton

penetration through the graphene layers. However, they used different graphene materials in these experiments. CVD graphene grown on copper was transferred using Fe(NO3)3 solution to etch off copper substrate, and the resultant samples were used to investigate proton transfer resistance, while CVD Graphene covered NiMo samples were employed as HER catalysts. These two samples are quite differences in defects and morphologies, which will give a misleading relation or conclusion. So authors needs to redesign their experiments to find out direct evidences showing HER activity is determined by proton transfer.

Reply: We appreciate the core question to improve our manuscript. We totally agreed that the difference between "transferred CVD graphene for proton penetration measurements" and "CVD graphene-encapsulated NiMo samples for HER measurements", and thus we re-performed the experiments by newly prepared transferred CVD graphene-covered Ni sheets. The transferred CVD graphene-covered Ni sheets were prepared based on the same graphene-transfer method used in the proton penetration experiments (see detailed preparation in the revised supplementary information). N-doped graphene monolayer, trilayer, and hexalayer were pasted on the Ni sheets (abbreviated as Ni/1NGL, Ni/3NGL, and Ni/6NGL, respectively) and their HER performances were measured by using a three-electrode system in 0.5M H_2SO_4 electrolyte. The HER activity shows a declining trend with the increase of layer number of graphene (**Figure R8a**). The bare Ni sheet needed an overpotential (η_{10}) of 455 mV vs. RHE at the current density of 10 mA cm^{-2}, normalized by the geometric area of the electrode, while η_{10} values of 515, 786, and 942 mV vs. RHE were recorded for the Ni/1NGL, Ni/3NGL, and Ni/6NGL, respectively. We clearly observed a linear correlation between the catalytic activity (i.e., η_{10} value) of graphene-covered Ni sheets and proton penetration ability (i.e., average 8-h CA current) (**Figure R8b**). As such, by using the same preparation method and quality of graphene sheets, we concluded out the "direct evidences showing HER activity is determined by proton transfer".

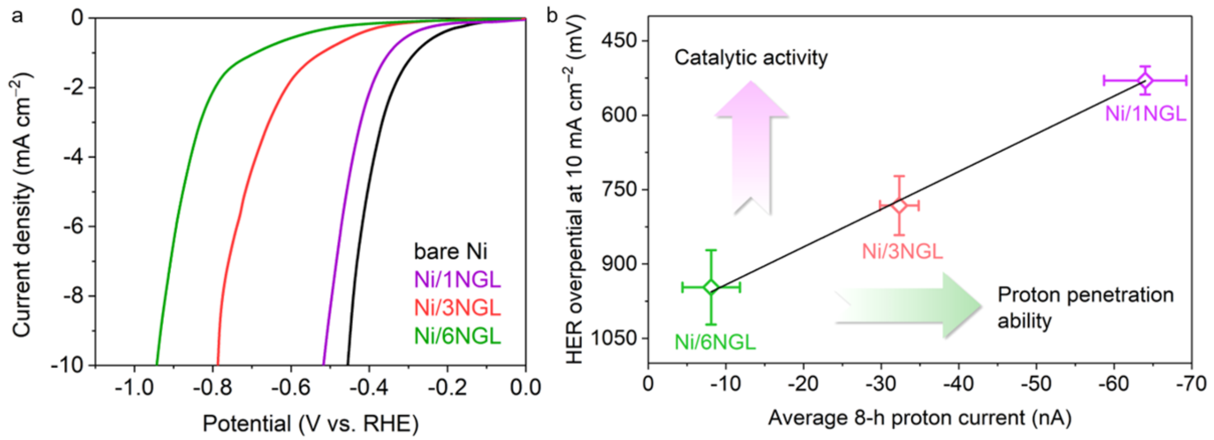

Figure R8. (a) HER polarization curves of bare Ni, Ni/1NGL, Ni/3NGL, and Ni/6NGL. (b) Correlation between the catalytic activity of the graphene-covered Ni sheets and the proton penetration through the graphene layers.

3 The unit for current in Figure 4a is not correct. And HER activity on Pt needs to remeasure.

Reply: We confirmed the unit used in **Figure 4a** in the original submission (**Figure 3d** in the revised manuscript) is reasonable. In fact, the same unit was used in the different literatures (for example, inset of **Figure 1**, Nature 516, 227-230 (2014).).

For the second point, we are sorry, but we could not understand why and for which Pt sample the HER activity should be re-measured. We suspect that the reviewer is concerned about the quality of our data. To check the consistency of our data with other's data, we measured the *I*−*V* curves for monolayer graphene. As shown in **Figure R9**, the remeasured *I*−*V* characteristic keeps almost the same with the prior data, which reproduced the reported *I*−*V* characteristic (Nature 516, 227-230 (2014)).

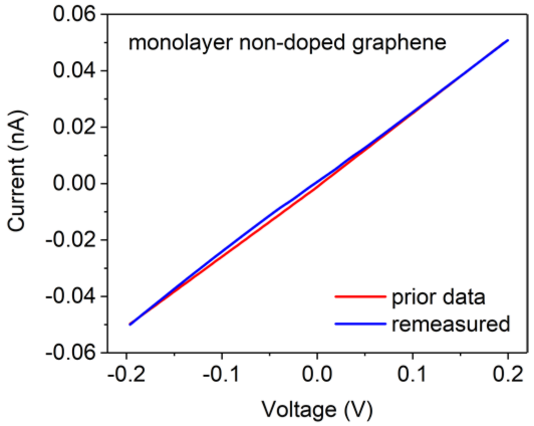

Figure R9. Remeasurement of I−V curves in the H-type cell with monolayer graphene membrane.

Reviewer #3 (Remarks to the Author):

In this work, Hu et al report two sets of measurements. First, they measure the HER activity of a non-noble metal catalyst covered with few-layered graphene. The number of graphene layers on the catalyst can be controlled with reasonable accuracy. Second, they measure proton transport through stacks of these graphene layers using micro-fabricated suspended graphene/Nafion devices. The number of graphene layers can be controlled exactly. Both the catalytic activity and the proton transport results were, to a large extend, measured in previous works. The novelty of this work is that the authors demonstrate that the activity of the catalyst as a function of graphene layers correlates perfectly with the proton conductivity through these layers. This is strong experimental evidence showing that the HER activity of the graphene-covered catalysts is governed by proton transport. This represents a shift in how these catalysts are understood and it came as a surprise. All previous works –including some by the authors themselves (ref. 8) – suggested that the HER on graphene covered catalysts took place on the surface of the graphene coating. The work has been carefully done and the manuscript is well written.

Reply: We appreciate the reviewer's deep understanding on our manuscript. We revised the manuscript to address your concerns.

The main concern with the present work is the exit of H2 molecules from the graphene-covered catalyst. The authors propose two exit mechanisms. One is that these molecules diffuse through nanometre (<8 A) pores in the lattice. Since the catalysts in this work are normally only a couple nanometres across, if such pores were present, a large proportion of their catalysts would not be covered by graphene. This would seem to contradict their finding that the graphene protects it against corrosion. Furthermore, their OCV measurements imply that their membranes are nearly perfectly selective between protons and anions in their electrolyte solution (L. Mogg et al. Nat. Comms. 4243, 2019). This is not possible if nanopores are present. The second proposed mechanism is that the H2 molecules split into two H atoms, diffuse through vacancies and recombine after transferring through the graphene coating. While this seems plausible, the authors should explain why this process does not limit their HER activity. This second recombination, which takes place without a catalyst, could be expected to be slower than the one on their catalyst. In any case, this discussion should be in the main text, rather than in Supplementary.

Reply: We thank and agree with the reviewer's comment. We investigated the size effect of graphene nanopores on their energy barrier values of proton penetration. In the Table S9 in supplementary information, the smallest pore, whose diameter is 3.5 Å, demonstrated a low energy barrier of 0.12–0.68 eV for the ejection of hydrogen molecules. In the case of graphene growth on nanoparticles or nanostructures, there were large-sized defects (2.0–4.0 Å) on highly curved graphene lattices (**Figure R10**, *Adv. Mater.,* **2016**, 28, 10644. and *Angew. Chem. Int. Ed.* **2018**, 57, 13302.). Thus, the generated hydrogen molecules can be ejected through such defects and the large nanopores (<8 Å) are not necessary. Therefore, our proposed mechanism is consistent with the defect-free graphene membrane (L. Mogg et al. Nat. Comms. 4243, 2019). We added these discussions to the revised manuscript and cited this important reference (L. Mogg et al. Nat. Comms. 4243, 2019) as reference 15.

For the second proposed mechanism, we agree with the reviewers' concern and rearranged the discussions. We evaluated the energy barrier and the order from high energy barrier to low energy barrier of hydrogen molecule penetration as follow.

Perfect graphene lattice, 5-7 and 5-8 defects >> split and recombination through N-doped graphene lattice > nanopores in Table S9 (no split)

Therefore, it is a dominant pathway that hydrogen molecules penetrate without the split and recombination. We moved the second proposed mechanism and these related discussions to the revised supporting information.

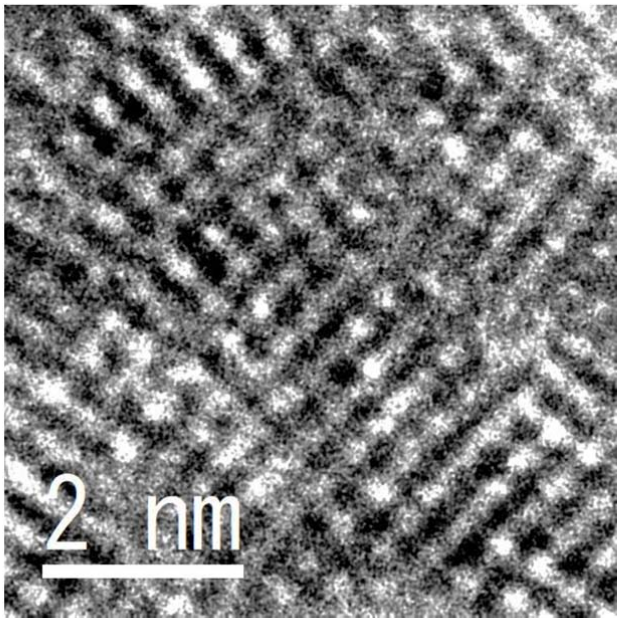

Figure R10. Direct observation of defects on the highly curved graphene lattice reproduced from *Angew. Chem. Int. Ed.* **2018**, 57, 13302.

One minor comment.

The authors report isotope experiments, but do not compare their results with their proton data. This would be of interest to the community.

Reply: We added the CA measurement and discussion about isotope experiments in supplementary

information according to the reviewer's suggestion. In short, the proton current of H^+ was 1.4-1.7 times higher than the proton current of D^+, which means the isotope effect surely appears as shown in **Figure R11**. We added these discussions and **Figure R11** to the revised manuscript and the supporting information.

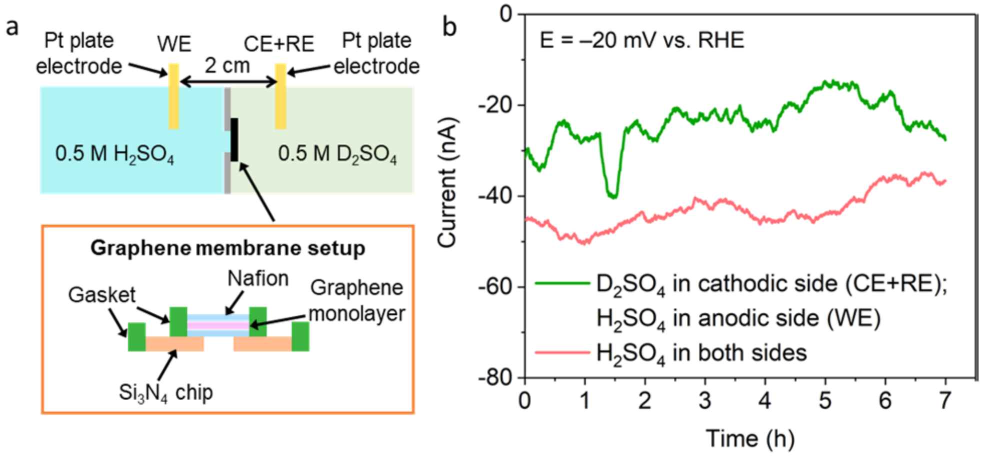

Figure R11. Proton currents collected by CA at a cathode potential of −20 mV vs. RHE through monolayer graphene in H_2SO_4 or D_2SO_4 electrolyte.

If the authors can address these comments, I would like to recommend for publication.

Reply: We appreciate the reviewer's deep understanding on our manuscript.

Reviewer #1 (Remarks to the Author):

The revised materials have addressed the concerns of the reviewer. However, presently two issues are still inconvincible.

The first concern is the penetration of proton through the multilayer graphene shell. The energy barriers of proton penetration for 6-7 layer N-doped graphene should be a very large value, in other words, the amount of proton that reached the surface of encapsulated metal is insufficient for the large and continuous reaction current, this speculate is also verified by the average proton current (nA) in Fig. 4. While for the present electrochemical data and the synthesis of 3D porous graphene parts, this would seem that the metal is not completely encapsulated within the graphene layers, since acid can easily etched the metal substrate.

The second concern is the diffusion of the generated H2 molecules under the cover between graphene layer and metal surface. Especially for the multi-layer (6-7 layer) N-doped graphene covered metal case, graphene nanopores with low energy barriers can be the dominant pathway for the ejection of H2 molecules. While bigger H2 bubble would not pass through the nanopores (<8 Å), thus impeding the HER reaction.

In a word, the encapsulating graphene layers are not complete with a large proportion of metal surface would not be covered. The conclusion of proton penetration would not be fully supported due to lack of appropriate model chosen in this work.

Reviewer #2 (Remarks to the Author):

I have read through the response to reviewers' comments and the revised manuscript. Authors run more experiments and added more data in the revision, but unfortunately did not address reviewers' concerns. So I do not recommend its publication in the journal.

1 Authors try to claim that for graphene covered metal catalysts, hydrogen evolution takes place at metal surface instead of graphene surface, which were challenge by two reviewers. They proposed a picture: protons transport through graphene coating, approach to metal surface and discharge to form hydrogen molecular, which then diffuse into bulk solution through nanopores or defects in graphene coating. If it is true, why this protective graphene coating allows hydrogen molecular to pass through but reject acidic solution?
Moreover, I still have a hard time believing that protons preferentially overcome a huge activation barrier (1.42 eV for 6NGL as claimed by authors) to accept electrons from metal catalysts rather than easily receive electrons on graphene surface (electrical resistance is a small number for 6NL). Why?

2 I do not think authors read the reference (Nature 516, 227-230 (2014)) carefully or understand it well. In the reference, proton transport activation barriers did linearly increase with the layer number (0.3 eV for monolayer hBN, and 0.6 eV for bilayer hBN), which is quite different from this work. In addition, proton conductance measured for pristine monolayer graphene in this work is over 10 times lower than the reference.

3 Author claimed that they could not understand why and for which Pt sample the HER activity should be re-measure. In fact, there is only one data about Pt (figure 1c) in the previous submission. To date, Pt or Pt based materials are still the best HER catalysts. In that figure, HER activity on Pt is much lower than bare NiMo and even 1-2 NGL in 0.5 M H2SO4 solution. Is that reasonable?

Reviewer #3 (Remarks to the Author):

In their reviewed manuscript, Hu et al report extensive new data sets. Two central points are addressed.

First, they present HER measurements on Cu and Ni foils encapsulated with CVD graphene. They find that these electrodes display the previously found correlation between catalytic activity and proton conductivity with the number of graphene layers. The key difference now is that both experiments use graphene grown with the same technique. This clears any reasonable doubt about the validity of the correlation. The new experiments strongly suggest the validity of their results beyond the specific metals tested in the study, which will broaden the impact of the work.

The second point is the exit of H_2 molecules from the encapsulated catalysts. The authors address this with DFT calculations that show that the energy barrier for H_2 exit through defects is consistent with their results. Indirectly, they also address this point experimentally. Indeed, in metal foils encapsulated with CVD graphene, the generated H_2 molecules are trapped between graphene and the metal due to the relatively low defect density in CVD graphene. (This experiment also serves as a visual demonstration that protons react between the metal layer and graphene.) This contrasts with curved graphene shells, which are known to possess a larger defect density and in which such gas trapping was not observed.

In my opinion, the authors have addressed the central concerns raised in the previous round of review. I would like to recommend this work for publication.

Reviewers' comments:

Reviewer #1 (Remarks to the Author):

The revised materials have addressed the concerns of the reviewer. However, presently two issues are still inconvincible.

Reply: We appreciate reviewer's fruitful comments and spending time to improve our manuscript again. We revised the manuscript to address your concerns.

The first concern is the penetration of proton through the multilayer graphene shell. The energy barriers of proton penetration for 6-7 layer N-doped graphene should be a very large value, in other words, the amount of proton that reached the surface of encapsulated metal is insufficient for the large and continuous reaction current, this speculate is also verified by the average proton current (nA) in Fig. 4.

Reply: We observed the proton current of 10 nA, which corresponds to 10 mA/cm^2 as the window area of the SiN chip used in this study is 100 μm^2, providing 10 nA/100 μm^2=10 mA/cm^2. We believe that the current of 10 mA/cm^2, which is commonly used as a benchmark of electrochemistry, is not too small to detect the continuous reaction current (as is shown in Small Methods 2018, 2, 1800168.).

Action: We changed the unit of the proton current from nA to mA/cm^2 in **Figure 4** to avoid the readers' confusion.

While for the present electrochemical data and the synthesis of 3D porous graphene parts, this would seem that the metal is not completely encapsulated within the graphene layers, since acid can easily etched the metal substrate.

Reply: Thank you for the detailed comment. We agree that the reviewer's concern about the incomplete encapsulation of the metal surface is valid. Therefore, we carried out the additional measurement to confirm that the metal surface is completely encapsulated by following an acid-

treatment method reported in Angew. Chem. Int. Ed. 2015, 54, 2100 (Prof. Xinhe Bao group).

In short, we measured and compared the HER polarization curves of NiMoNP/6-7NGL samples with and without soaking in 0.5M H_2SO_4 electrolyte for 100 h at 20 ºC. The as-prepared NiMoNP/6-7NGL sample (**Figures R1a−c**) showed the very similar HER activity before and after soaking in acid for 100 h (**Figure R1d**), and no obvious degradation was observed (**Figures R1e**, **f**), which confirmed the complete encapsulation. On the other hand, the incompletely encapsulated NiMoNP/6-7NGL sample prepared through a 30 sec oxygen plasma-treatment (**Figures R1g, h**) showed an irradiated morphology with high defect density (I_D/I_G ratio: from 0.87 to 1.28) and then demonstrated a totally different electrochemical behavior: the soaking induced the drastic reduction of the HER current (**Figure R1d**). If the encapsulation of the metal surface is not completed, one can see the similar drastic reduction of the HER current, which is not the case in our samples. These demonstrate that our samples are completely encapsulated by the graphene layers.

Action: We added these points in revised supplementary information (**Figure R1** as **Figure S35,** discussions as Supplementary discussion 6).

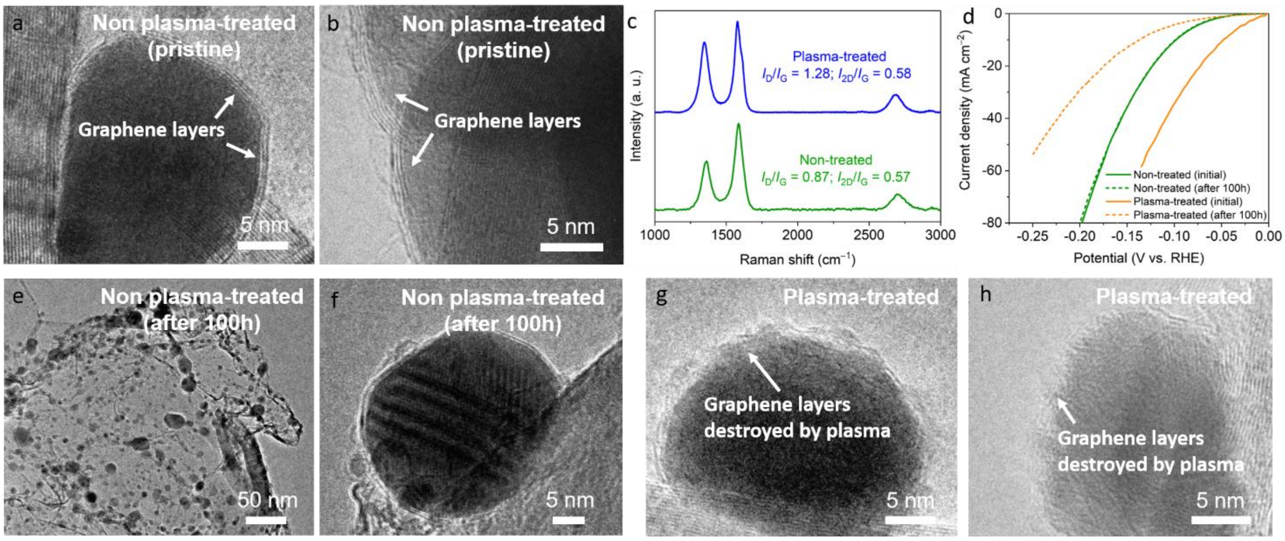

Figure R1. Characterizations and HER activities of the NiMoNP/6-7 sample with and without oxygen plasma treatments. TEM images of the as-prepared/non-plasma-treated NiMoNP/6-7NGL sample (a, b) before and (e, f) after 100-h soaking in 0.5M H_2SO_4. (c) Raman spectra of non- and plasma-treated NiMoNP/6-7NGL samples. (d) HER polarization curves of the non- and plasma-treated NiMoNP/6-7NGL samples before and after 100-h soaking in 0.5M H_2SO_4. Dashed curves represent that the samples were measured after soaking in 0.5M H_2SO_4 for 100 h at 20 ºC. During the 100-h soaking, the samples were immersed in 0.5 M H_2SO_4 electrolyte. (g, h) TEM images of plasma-treated NiMoNP/6-7NGL sample.

The second concern is the diffusion of the generated H_2 molecules under the cover between graphene layer and metal surface. Especially for the multi-layer (6-7 layer) N-doped graphene covered metal case, graphene nanopores with low energy barriers can be the dominant pathway for the ejection of H_2 molecules. While bigger H_2 bubble would not pass through the nanopores (<8 Å), thus impeding the HER reaction.

In a word, the encapsulating graphene layers are not complete with a large proportion of metal surface would not be covered. The conclusion of proton penetration would not be fully supported due to lack of appropriate model chosen in this work.

Reply: Thank you for the useful comment. The reviewer claimed that if the metal surface is completely covered by the graphene, a bigger H_2 bubble cannot pass the nanopores, impeding the HER reaction. Since this does not happen in our system, the reviewer thinks that the metal surface is insufficiently covered.

First of all, we believe that the controlled measurement with an acid treatment mentioned above convinces the reviewer that our metal surface is fully covered by the graphene. A remaining question is thus how a big bubble with its radius of > 1nm can pass the nanopores of the N-doped graphene if it is formed in-between the N-doped layers and/or in-between the N-doped layer and metal surface. Through manual operation of suction by a pipette, we confirmed that the generated H_2 bubbles (**Figure R2**) were attached on the N-doped graphene (6-7 layer), and we could not find the bubbles in-between NGL and Ni surface after tossing all removable bubbles (**Figure R3a**). This is in stark contrast with the GL (6-7 layer) case, where one can clearly see the bubble encapsulation was observed only at the low potential range from 0.0 to −0.2 V (vs. RHE) with a slow scan speed (0.01 mV/s) (**Figure R3b**).

The bubble encapsulation formation in the GL-covered sample and the absence in the NGL-covered sample further affect the graphene structure after the HER reaction. We observed that the Raman mapping of G band intensity and I_D/I_G intensity ratio before and after reaction did not show any significant change for the NGL-covered Ni sheet (**Figure R4a**), while the GL-covered Ni sheet showed the crack of graphene (**Figure R4b**). These differences can be attributed to the balance between the permeation of molecular hydrogen through graphene layers dominated by defects/nanopores and the generation speed of molecular hydrogen dominated by experimental parameters (i.e. scan speed and potential range).

Recently, the study of the Prof. Geim group attributed the H_2 permeation through a graphene layer to the ripples and defects inducing a local curvature as catalytically active sites (Nature, 579, 2020, 229.). However, with this mechanism, the H_2 permeation speed through GL seems very slow,

consistent with our observations that the HER process was impeded by bubbles for the GL encapsulation. On the other hands, in our NGL system, H_2 permeation can be accelerated in the presence of small nanopore induced by N-dopants, which also have low activation energies for H_2 permeation (**Table S11**). Therefore, one can conclude that H_2 can be ejected from the NGL layer by the faster permeation of the molecular hydrogen through the defects/nanopores in NGL, not impeding the HER process.

Action: We added this discussion in the revised manuscript (Page 11−12) and supplementary information (**Figure R2/R3/R4** as **Figure S44/S45/S46**, and discussions as Supplementary discussion 8)

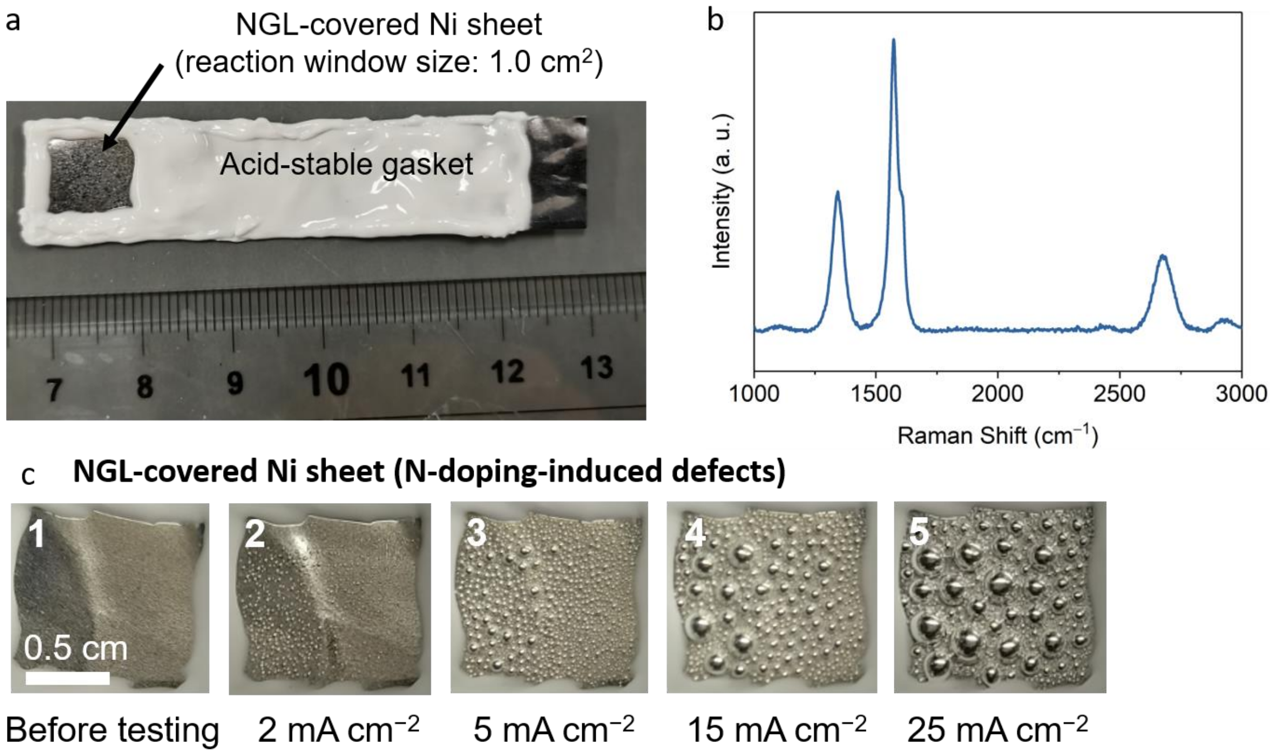

Figure R2. Hydrogen bubble formation on N-doped graphene (NGL)-covered Ni sheet. (a) Configuration of Ni sheet electrode covered by an insulator gasket. (b) Raman spectra of the N-doped graphene-covered Ni sheet. (c) Current density dependence of bubble formation on NGL-covered Ni sheet. Scan speed: 1.0 mV/s, potential range from 0.0 to −0.9 V vs. RHE, linear sweep mode).

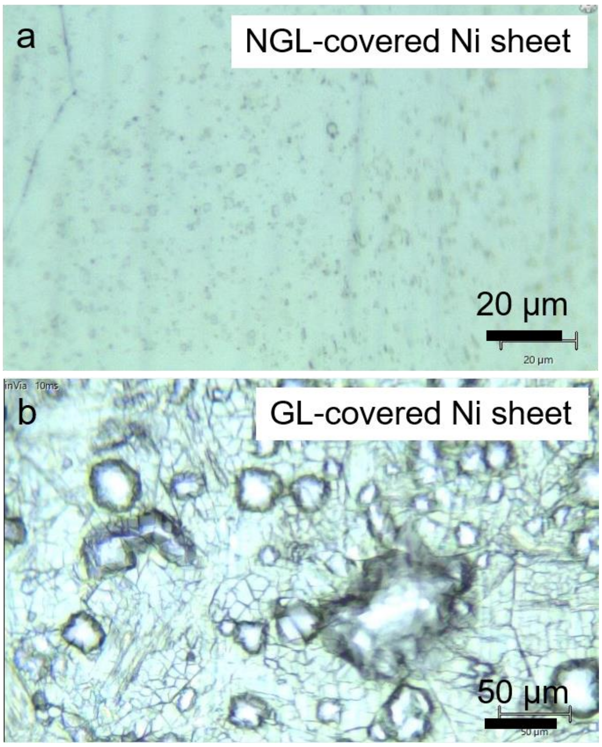

Figure R3. H_2 bubble formation of (a) NGL- and (b) GL-covered Ni sheets during HER process. The linear scan was carried out in the range from 0.0 to −0.2 V vs. RHE with a slow scan speed of 0.01 mV/s. The bubbles formed in the interface between Ni and GL, which were encapsulated by GL. In contrast, no encapsulated H_2 bubbles were observed in the NGL-covered Ni sheet.

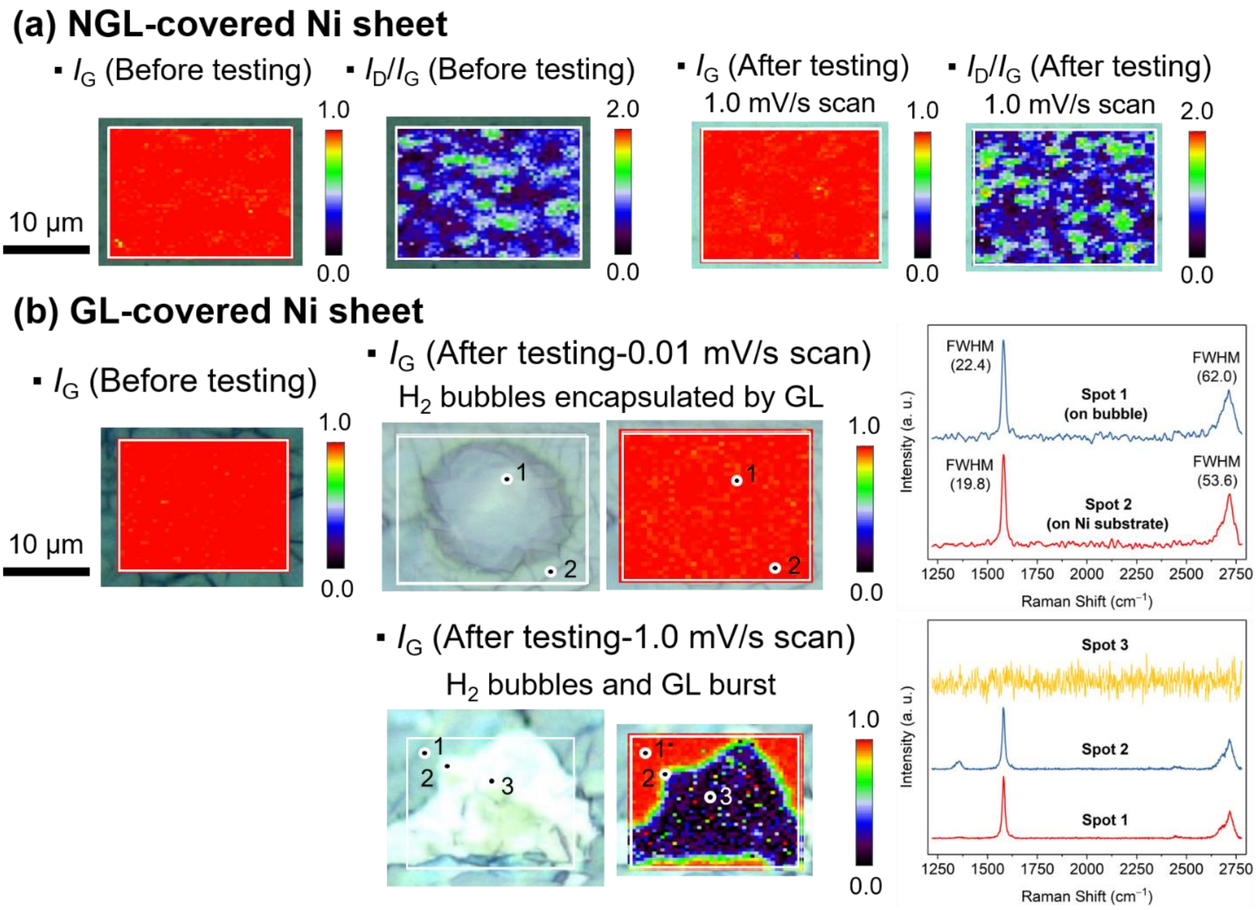

Figure R4. Raman spectrum and mapping images of (a) NGL- and (b) GL-covered Ni sheets before and after testing in 0.5 M H_2SO_4 electrolyte. Scan speed: 0.01 mV/s for a slow scan and 1.00 mV/s for a fast scan on the GL sample; 1.00 mV/s for scan on the NGL sample. For the I_G mapping results, the G band intensity of all Raman spectra was normalized to 1. The full width at half maximum (FWHM) values of G and 2D bands of the GLs on encapsulated H_2 bubble (Spot 1) and Ni substrate (Spot 2) were presented.

Reviewer #2 (Remarks to the Author):

I have read through the response to reviewers' comments and the revised manuscript. Authors run more experiments and added more data in the revision, but unfortunately did not address reviewers' concerns. So I do not recommend its publication in the journal.

Reply: We appreciate reviewer's fruitful comments, which are helpful to further improve our manuscript. We revised the manuscript by following the reviewer's concerns.

1 Authors try to claim that for graphene covered metal catalysts, hydrogen evolution takes place at metal surface instead of graphene surface, which were challenge by two reviewers. They proposed a picture: protons transport through graphene coating, approach to metal surface and discharge to form hydrogen molecular, which then diffuse into bulk solution through nanopores or defects in graphene coating. If it is true, why this protective graphene coating allows hydrogen molecular to pass through but reject acidic solution?

Reply: We agreed with the important questions which should be mentioned in the manuscript. The ions in an acidic solution is hydrated by 5-6 water molecules in average and is tightly bound to surrounding water (Science, 2010, 328, 1006-1009). Rearranging the solvation structure of ions and surrounding water molecules requires additional energy, prohibiting the ions to penetrate the graphene. On the other hand, proton transfer in water can be achieved through proton hopping process, without drastic rearrangement of the surrounding water molecules. Moreover, charge state of molecular hydrogen is neutral so that it is easier to pass through in comparison to the cation surrounded with hydrated water molecules. In summary, it is more favorable for the charge-neutral molecular hydrogen to pass through graphene layers instead of the large-sized hydrated proton, which explains that the graphene coating prefers to allow the pass of molecular hydrogen rather than protons.

Action: We added this discussion in the revised manuscript (Page 11).

Moreover, I still have a hard time believing that protons preferentially overcome a huge activation barrier (1.42 eV for 6NGL as claimed by authors) to accept electrons from metal catalysts rather than easily receive electrons on graphene surface (electrical resistance is a small number for 6NL). Why?

Reply: We agree with the reviewer that 1.42 eV activation energy is too high to overcome only with thermal energy. However, in our study, we used an applied electric field by a potentiostat, which drives the electrochemical reaction. To study the influence of the externally applied electric field on the local field in the vicinity of the graphene layers, we newly carried out the vacuum/water/graphene/water/vacuum system under a 0.5×10^9 V/m (= 0.5 V/1 nm) electric field (**Figure R5**). In the simulation, the maximum voltage dropped significantly at the interface between water layer and graphene surface and the voltage drop was equivalent to 0.22 V. This result is consistent with the notion that the dielectric constant near the bilayer graphene is extremely low (Nano Lett. 2013, 13, 3, 898.), causing a voltage drop at the layers.

To highlight the importance of the applied electric field for dividing the electrochemical reaction, we experimentally changed the applied voltage and observed the variation of the activation energy for the proton penetration. The 6NGL was employed as separating membranes with some similar experimental conditions in **Figure 3d** and the range of applied voltage was expanded to −3.5 to +3.0 V (**Figure R6**). We found that the *I*−*V* curve showed a non-linear behavior and estimated the "apparent" activation energy from −3.5 to −3.0 V as 0.43 ± 0.02eV. This "apparent" activation energy was changed by the electric field and becomes much lower than that (1.42 ± 0.02eV) from −0.2 to 0.0 V (i.e., at absence of the electric field). This can be explained by the fact that protons overcome the activation energy with the aid of applied electric filed. In the nanoparticle morphology, the voltage drop could increase when morphology changes to the nanoparticle shape.

Moreover, the reason why protons receive electrons in the interface not on graphene surface is their overpotential of HER. In Figure 2, the bare 3NGL and 6NGL showed much higher overpotential to receive electrons for producing H_2 than Ni/3NGL and Ni/6NGL. This indicates that

bare NGL actually can provide electrons but need high overpotential due to the nature of catalytic activity. Therefore, it is energetically preferable that the proton received electrons in the interface.

Action: We added this discussion in the revised manuscript (Page 8) and supplementary information (**Figure R5, R6** as **Figure S23, S22**).

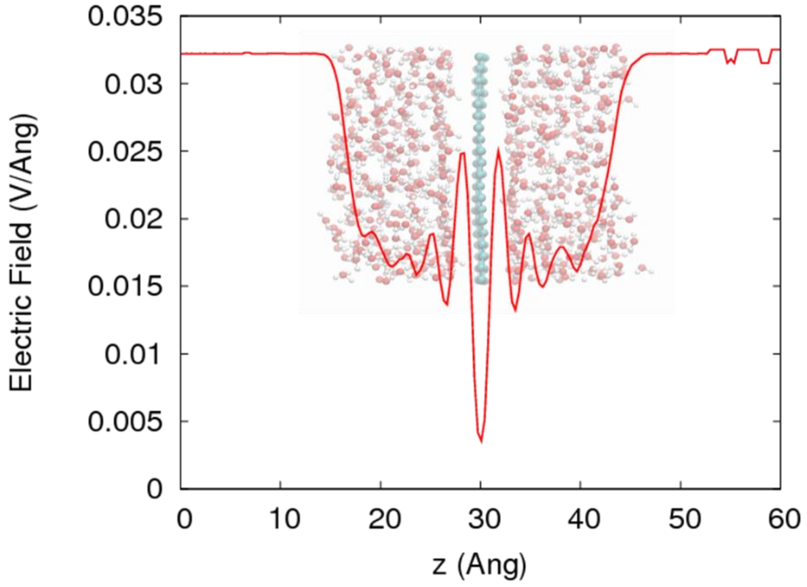

Figure R5. Electric field behaviors in water/graphene/water model.

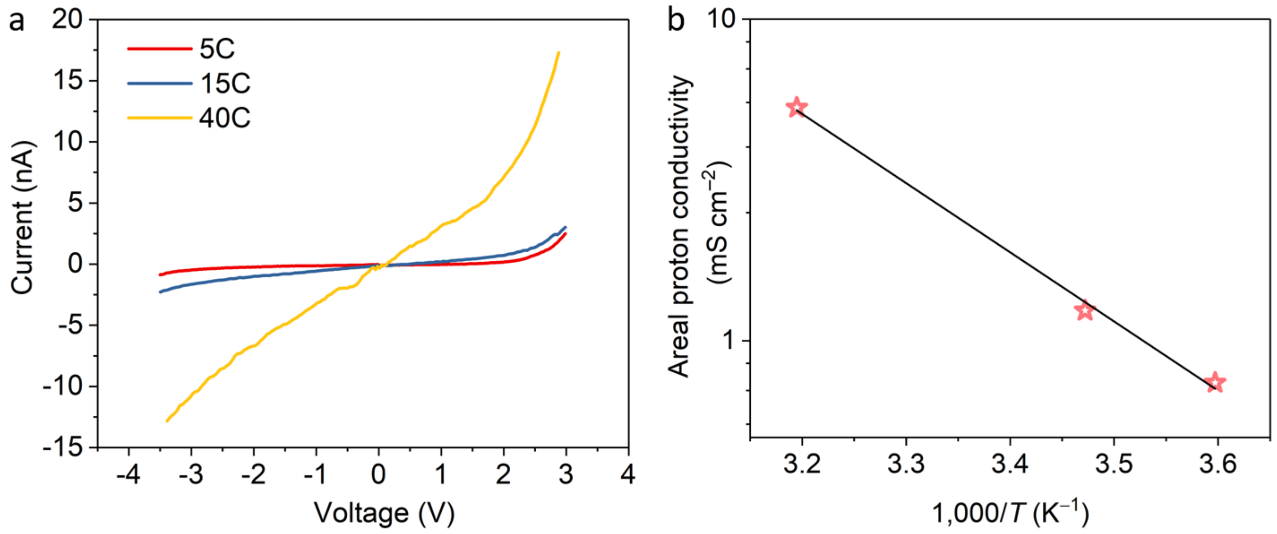

Figure R6. Experimental "apparent" activation energy of proton penetration through 6NGL at the wide range of cell voltage. (a) *I*−*V* characteristics of proton penetration through 6NGL at various temperatures. (b) Temperature dependence of proton conductivity for 6NGL under high cell voltage (−3.5 ~ −3.0 V).

2 I do not think authors read the reference (Nature 516, 227-230 (2014)) carefully or understand it well. In the reference, proton transport activation barriers did linearly increase with the layer number (0.3 eV for monolayer hBN, and 0.6 eV for bilayer hBN), which is quite different from this work. In addition, proton conductance measured for pristine monolayer graphene in this work is over 10 times lower than the reference.

Reply: To address the comment of the activation energy as a function of layer number, we measured the activation energy barrier of proton penetration through bilayer non-doped graphene (defect-less) (**Table R1**), since the activation energy of hBN sheet (defect-less or defect-free) is proportional to the layer number. We found that the activation energy for bilayer non-doped graphene (2GL) (1.76 ± 0.04 eV) is also proportional to the layer number (**Figure R7**). Thus, the linear increase of the activation energy barrier seems to appear only in the defect-less or defect-free 2D materials, such as non-doped graphene or hBN. In a sharp contrast, the activation energy barriers for N-doped graphene

does not follow the linear relationship (0.87 ± 0.03 eV for 1NGL; 1.17 ± 0.02 eV for 3NGL; 1.42 ± 0.02 eV for 6NGL), because the doping-induced defects/nanopores in graphene lattices accelerates proton penetration. Our data demonstrates that the defects play a critical role of tuning activation energy, and the defect-free or defect-less graphene and hBN case is different with defective N-doped graphene case.

Moreover, we did literature searches for the proton conductivity values estimated by proton penetration experiments through monolayer graphene. As shown in **Table R2**, the experimentally measured proton conductivity values varied based on the device setting, testing methods, temperatures, pH values, etc. from different researchers. Importantly, it is reported that the proton conductivity strongly varies due to contributions from the unavoidable defects in CVD graphene (Concluded by ACS Nano, 2019, 13, 12109-12119, Page 12116). Thus, we investigated the defect amount (i.e. I_D/I_G) dependence of proton conductivity on monolayer graphene (**Figure R8**). We found that the proton conductivity varied from 0.16 to 0.6 mS cm^{-2}. Thus, the proton conductivity must be tuned by the number of defects in CVD graphene used in each research. Therefore, our results are in the acceptable range.

Action: We added this discussion in the revised manuscript (Page 7; **Figure R7** as revised **Figure 3g**) and supplementary information (**Figure R8** as **Figure S15**; **Table R1, R2** as **Table S5, S3**).

Table R1. Experimental values of energy barrier of proton penetration through various types of graphene.

Graphene sample	Energy barrier (eV)	Reference
1GL	0.95 ± 0.03	This work
2GL	1.76 ± 0.04	This work
1NGL	0.87 ± 0.03	This work
3NGL	1.17 ± 0.02	This work
6NGL	1.42 ± 0.02	This work
1GL	0.78 ± 0.03	Nature, 516, 227 (2014)

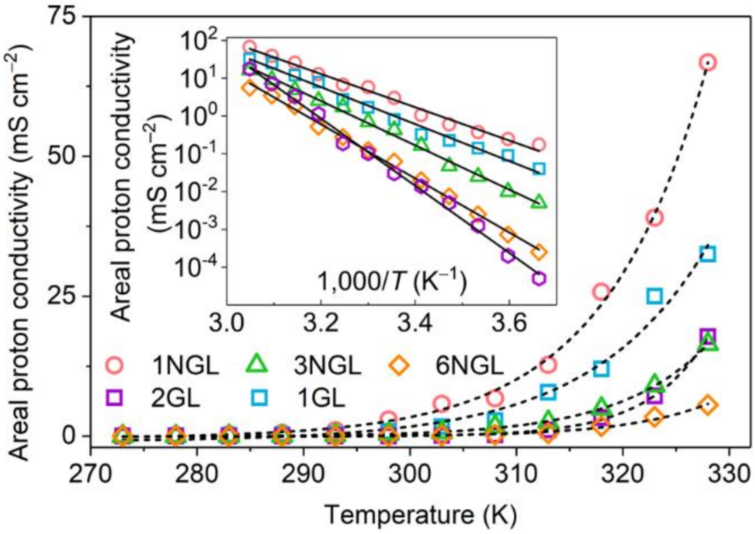

Figure R7. Temperature dependences of proton conductivity for 1GL, 2GL, 1NGL, 3NGL, and 6NGL. Inset: log σ as a function of T^{-1}. The solid and dashed curves are the fits to the experimental results.

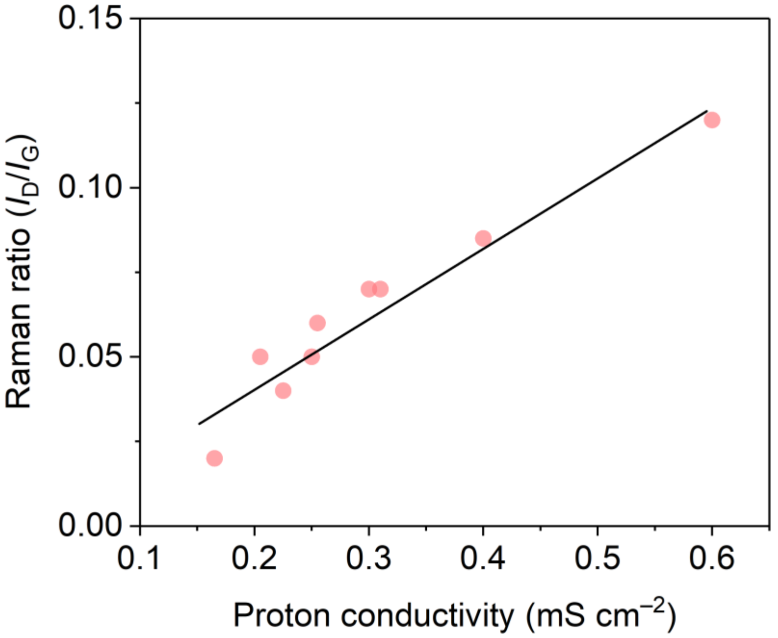

Figure R8. Defect amount dependence of proton conductivity on monolayer graphene.

Table R2. Comparison of proton conductivity values for proton penetration through monolayer graphene.

Proton conductivity and conditions	Experimental energy barrier (eV)	Reference
0.16-0.6 mS cm^{-2} at 20 °C	0.95 ± 0.03	This work
~2 mS cm^{-2} at 21–23 °C	0.78 ± 0.03	Nature, 516, 227-230 (2014)
26.3 mS cm^{-2} at 30 °C	0.50 ± 0.05	Electrochim. Acta, 296, 1 (2019)
125 mS cm^{-2} at 60 °C	0.50 ± 0.05	Electrochim. Acta, 296, 1 (2019)
29 mS cm^{-2} at 30 °C	Not reported	J. Am. Chem. Soc., 140, 1743 (2018)
~4 mS cm^{-2} at room temperature	Not reported	ACS Nano, 13, 12109 (2019)

3 Author claimed that they could not understand why and for which Pt sample the HER activity should be re-measure. In fact, there is only one data about Pt (figure 1c) in the previous submission. To date, Pt or Pt based materials are still the best HER catalysts. In that figure, HER activity on Pt is much lower than bare NiMo and even 1-2 NGL in 0.5 M H_2SO_4 solution. Is that reasonable?

Reply: We are sorry to misunderstand the previous comment. We remeasured Pt/C catalyst (newly purchased and well activated) by CV (**Figures R9** and **R10**), which is a reasonable value compared with reported literatures. Moreover, we do not insist that our non-noble-metal catalyst exceeds Pt and Pt-based catalyst in the manuscript.

Action: We replaced the CV data in the revised supplementary information (**Figures S25d** and **S28a**).

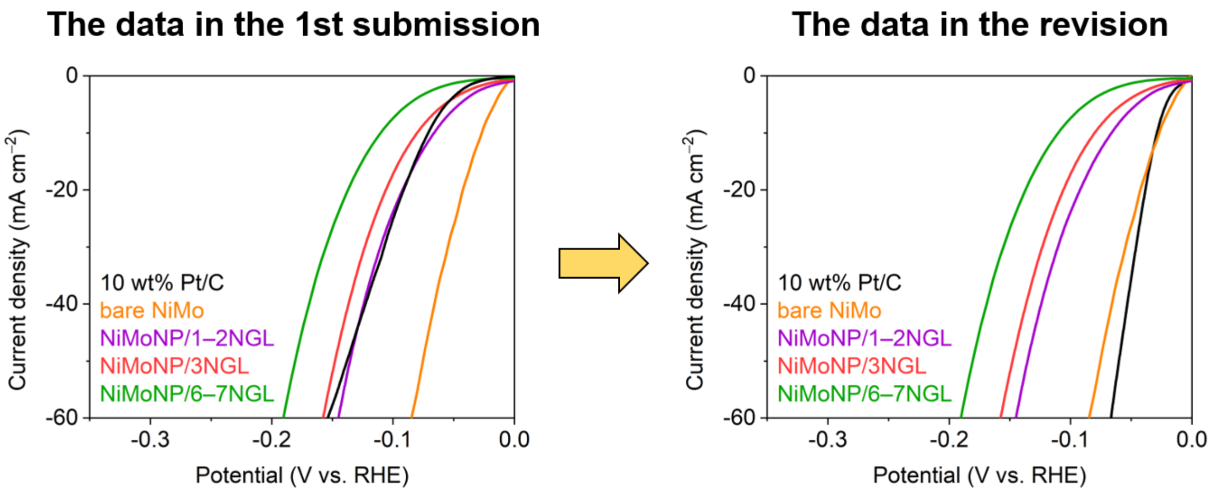

Figure R9. HER polarization curves with remeasured date of Pt/C catalysts.

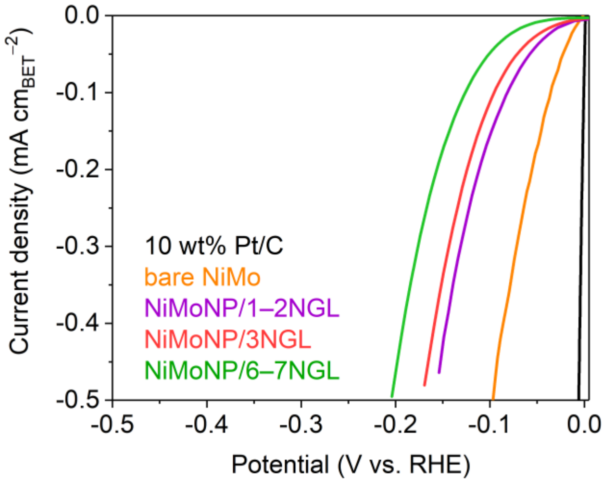

Figure R10. BET-surface-are-normalized HER polarization curves.

Reviewer #3 (Remarks to the Author):

In their reviewed manuscript, Hu et al report extensive new data sets. Two central points are addressed.

Reply: We appreciate the reviewer's deep understanding on our manuscript. We revised the manuscript to address your concerns.

First, they present HER measurements on Cu and Ni foils encapsulated with CVD graphene. They find that these electrodes display the previously found correlation between catalytic activity and proton conductivity with the number of graphene layers. The key difference now is that both experiments use graphene grown with the same technique. This clears any reasonable doubt about the validity of the correlation. The new experiments strongly suggest the validity of their results beyond the specific metals tested in the study, which will broaden the impact of the work.

Reply: We appreciate all reviewers' professional and fruitful comments on our manuscript.

The second point is the exit of H_2 molecules from the encapsulated catalysts. The authors address this with DFT calculations that show that the energy barrier for H_2 exit through defects is consistent with their results. Indirectly, they also address this point experimentally. Indeed, in metal foils encapsulated with CVD graphene, the generated H_2 molecules are trapped between graphene and the metal due to the relatively low defect density in CVD graphene. (This experiment also serves as a visual demonstration that protons react between the metal layer and graphene.) This contrasts with curved graphene shells, which are known to possess a larger defect density and in which such gas trapping was not observed.

Reply: We appreciate the reviewer's deep understanding on our manuscript. This is the one we insist here.

In my opinion, the authors have addressed the central concerns raised in the previous round of review.

I would like to recommend this work for publication.

Reply: Thanks!

Reviewer #2 (Remarks to the Author):

It could be accepted.

Reviewers' comments:

Reviewer #2 (Remarks to the Author):

It could be accepted.

Reply: We appreciate the reviewer’s deep understanding on our manuscript.
